# Supplementary material for: Spatially resolved reaction–diffusion modeling reveals effects of intracellular spatial heterogeneity on yeast galactose network dynamics
Source: PLoS Comput Biol. 2026 Oct 5;22(10):e1014811. doi: 10.1371/journal.pcbi.1014811 (PMC13705588; doi:10.1371/journal.pcbi.1014811)
Supplement: Supplementary file 1 [file pcbi.1014811.s001.pdf]

Appendix for Spatial Heterogeneity Alters the Dynamics of the Yeast Galactose Switch: Insights from 4D RDME–ODE Hybrid Simulations

Tianyu Wu[1,2], Marie-Christin Spindler[3,2], Abner T. Apsley[5], Emmy Earnest[4], Zane R. Thornburg[5,6], Julia Mahamid[3,7,2*], Zaida Luthey-Schulten[8,2,1*]

1 Center for Biophysics and Quantitative Biology, University of Illinois at Urbana–Champaign, Urbana, IL, USA
2 National Science Foundation Science and Technology Center for Quantitative Cell Biology, Beckman Institute for Advanced Science and Technology, University of Illinois at Urbana–Champaign, Urbana, IL, USA
3 Molecular Systems Biology Unit, European Molecular Biology Laboratory (EMBL), Heidelberg, Germany
4 Department of Physics, University of Illinois at Urbana–Champaign, Urbana, IL, USA
5 Beckman Institute for Advanced Science and Technology, University of Illinois at Urbana-Champaign, Urbana, IL, USA
6 Cancer Center at Illinois, University of Illinois at Urbana-Champaign, Urbana, IL, USA
7 Cell Biology and Biophysics Unit, European Molecular Biology Laboratory (EMBL), Heidelberg, Germany
8 Department of Chemistry, University of Illinois at Urbana–Champaign, Urbana, IL, USA

* julia.mahamid@embl.de * zan@illinois.edu

Contents

1 Species, reactions and parameters in the simulation **3**
- 1.1 Summary of parameterization strategy . . . 3
- 1.2 Species . . . 3
- 1.3 Initial counts of species . . . 5
- 1.4 Chemical reactions in the RDME system . . . 7
- 1.5 Chemical reactions in the system . . . 11
- 1.6 Diffusion Coefficients for species in RDME and ODE . . . 12

2 Geometry Reconstruction **12**
- 2.1 Geometric Reconstructions Parameters . . . 13
- 2.2 Chromosome model . . . 15
- 2.3 Nuclear Pore . . . 16

3 Additional Simulation Results **17**
- 3.1 Additional *GAL*-related species for Section 3.1 . . . 17
- 3.2 Additional *GAL*-related species for Section 3.2 . . . 18
- 3.3 Additional *GAL*-related species for Section 3.3 . . . 19
- 3.4 Additional *GAL*-related species for Section 3.4 . . . 20
- 3.5 Ribosome in Translation . . . 21

3.6 Comparison of Repressor Dynamics in the Nucleoplasm and Cytoplasm Between RDME-ODE and CME-ODE Models 22
3.7 Comparison of Activator and Repressor Dynamics in the Nucleoplasm and Cytoplasm Between Non-Chromosome and Chromosome-Included Models 23
3.8 Gene Positioning in the Nucleus Has Minimal Impact on Activation Dynamics 23
3.9 Ribosome Number and Spatial Organization Jointly Limit GAL2 Translation 24
3.10 Ablation analysis of factors contributing to CME–ODE and RDME–ODE differences 25
3.11 Obstacle perturbation analysis of local GAL2 promoter accessibility . . 27

4 Reduced Nucleoplasmic Diffusion and Gene Positioning 28

5 Comparison of Predicted and Measured Final Fold Changes of GAL Proteins Across Experimental and Modeling Studies 29

6 Comparison of promoter-binding propensity in CME and RDME 29

7 Mathematical interpretation of ER-dependent translation and trafficking 33

8 Sensitivity Analysis on kinetic parameters 34

1 Species, reactions and parameters in the simulation

1.1 Summary of parameterization strategy

Table A. Summary of how reaction and transport parameters were handled in the present RDME–ODE model. Full numerical values are provided in the detailed tables below. The main sources are from our previous works [1, 2] where the original literature values are cited.

Parameter / reaction class	Role in model	Source	Handling in this study
Core GAL regulatory binding/unbinding rates	Promoter regulation, Gal4/Gal80 interactions, transcriptional switching	Bianchi et al. [1]	Transferred to the RDME–ODE model as baseline biochemical kinetic parameters
Transcription rates	mRNA production for *GAL* genes and reporter	Bianchi et al. [1]	Retained as baseline rates; applied within the spatially resolved framework
Translation rates	Protein production from mRNA species	Bianchi et al. [1]	Retained, with spatial constraints added in the RDME–ODE model (e.g., ER-associated translation where applicable)
mRNA degradation rates	mRNA turnover	Bianchi et al. [1]	Retained unchanged
Protein / complex degradation rates	Protein turnover and complex removal	Bianchi et al. [1]	Retained unchanged
Metabolic ODE reaction rates	High-copy-number galactose transport/metabolism subsystem	Earnest et al. [2]	Retained in the ODE subsystem; coupled to the RDME portion in the hybrid model
Diffusion coefficients	Spatial transport of RDME species	Earnest et al. [2]	Added specifically for the spatial implementation; listed separately in Section 1.6
Nuclear import/export or compartment-transfer terms	Exchange between cytoplasm and nucleoplasm / other compartments	Earnest et al. [2]	Implemented in the spatial framework with explicit compartment structure
Initial molecular abundances	Starting copy numbers of species	Bianchi et al. [1]	Retained or adjusted according to compartmentalized spatial initialization

1.2 Species

Table B. Species and their annotations

Species	Annotation
DG_1	gene encoding Gal1 with nothing bound (free)
$DG_1 : G_{4d}$	gene encoding Gal1 bound to Gal4 dimer(activated)
$DG_1 : G_{4d} : G_{80d}$	gene encoding Gal1 bound to the Gal4 dimer and Gal80 dimer(repressed)
DG_2	gene encoding Gal2 with nothing bound(free)
$DG_2 : G_{4d}$	gene encoding Gal2 bound to the Gal4 dimer(activated)

Species	Annotation
$DG_2 : G_{4d} : G_{80d}$	gene encoding Gal2 bound to the Gal4 dimer and Gal80 dimer(repressed)
DG_3	gene encoding Gal3 with nothing bound (free)
$DG_3 : G_{4d}$	gene encoding Gal3 bound to the Gal4 dimer(activated)
$DG_3 : G_{4d} : G_{80d}$	gene encoding Gal3 bound to the Gal4 dimer and Gal80 dimer(repressed)
DG_4	gene encoding Gal4 with nothing bound(activated)
DG_{80}	gene encoding Gal80 with nothing bound(free)
$DG_{80} : G_{4d}$	gene encoding Gal80 bound to the Gal4 dimer(activated)
$DG_{80} : G_{4d} : G_{80d}$	gene encoding Gal80 bound to the Gal4 dimer and Gal80 dimer (repressed)
DG_{rep}	gene encoding the reporter fluorescent protein with nothing bound(free)
$DG_{rep} : G_{4d}$	gene encoding reporter bound to the Gal4 dimer(activated)
$DG_{rep} : G_{4d} : G_{80d}$	gene encoding reporter bound to the Gal4 dimer and Gal80 dimer(repressed)
R_1	mRNA for Gal1
R_2	mRNA for Gal2
R_3	mRNA for Gal3
R_4	mRNA for Gal4
R_{80}	mRNA for Gal80
R_{rep}	mRNA for the reporter gene
G_1	galactokinase metabolizes galactose
G_2	galactose transporter
G_3	galactose sensing transcription factor
G_{3i}	Gal3 bound to a galactose molecule
G_{4d}	the transcriptional activator
G_4	the monomer of the transcriptional activator
G_{80d}	the transcriptional repressor dimer
G_{80}	the monomer of the transcriptional repressor
$G_{80d} : G_{3i}$	Gal80 dimer bound to Gal3i; the transcriptional repressor sequestered in the cytoplasm
G_{rep}	a yellow/green fluorescence reporter protein (YFP)
GAE	extracellular galactose
GAI	intracellular galactose
G_2GAI	galactose bound to the Gal2 transporter on the intracellular side
G_2GAE	galactose bound to the Gal2 transporter on the extracellular side
G_1GAI	galactose bound to the Gal1 transporter on the intracellular side

1.3 Initial counts of species

Species	State	Region	Count
DG1	DG1_G4d_G80d	nucleoplasm	1
DG2	DG2_G4d_G80d	nucleoplasm	1
DG3	DG3_G4d_G80d	nucleoplasm	1
DG80	DG80_G4d_G80d	nucleoplasm	1
DGrep	DGrep_G4d_G80d	nucleoplasm	1
DG4	DG4	nucleoplasm	1
R1		cytoplasm	0
R2		cytoplasm	0
R3		cytoplasm	1
R4		cytoplasm	0
R80		cytoplasm	1
Rrep		cytoplasm	0
G1		cytoplasm	132
G2		Plasma membrane	1,156
G3		cytoplasm	4,341
G4d		nucleoplasm	308
Grep		cytoplasm	132
G80		cytoplasm	0
G80		nucleoplasm	0
G80d		cytoplasm	157
G80d		nucleoplasm	157
ribosome		ribosome	345,964

Table C. Species initial counts in RDME for no-chromosome and with-chromosome cases. If not mentioned, the initial counts are 0. Species counts are rounded to integers from Ramsey *et al.* [3].

Species	State	Region	Count
ribosome		ribosome	0
cytosolic ribosome		ribosome	338,131
ER-associated ribosome		ribosome	7,833

Table D. Change in species initial counts when switching from the no-chromosome/with-chromosome models to the ER-inclusive case; species not shown remain unchanged.

Species	State	Region	Count
cytosolic ribosome		ribosome	4,152
dummy cytosolic ribosome		ribosome	333,979
ER-associated ribosome		ribosome	4,256
dummy ER-associated ribosome		ribosome	3,577

Table E. Changes in initial species counts when transitioning from the ER-inclusive model to the effective-ribosome model. Species not shown remain unchanged. "Dummy" denotes ribosomes that are unavailable for translating galactose-switch mRNAs due to competition from non-switch transcripts.

Species	Concentration(mol/L)
G1	7.23×10^{-9}
G2	7.97×10^{-8}
GAI	0
G1GAI	0
G2GAE	0
G2GAI	0

Table F. Species initial concentration in ODE.

To be consistent, G1 and G2 concentration is the initial counts in RDME divided by $NA \cdot V$*.The species counts are rounded to integers from Ramsey et al. [3].*

1.4 Chemical reactions in the RDME system

These are all reactions included in the RDME system, they are grouped based on their functions (regulation, transcription, translation, mRNA degradation and protein/complex degradation).
Kinetic parameters are adopted from previously published studies [1].

Table G. Summary of gene regulation.

Chemical Reaction	Order	Region	Kinetic Parameters	Comments
$D_{G1} + G_{4d} \longrightarrow D_{G1}{:}G_{4d}$	2	nucleoplasm	$3.583 \times 10^7 \mathrm{M}^{-1}\mathrm{s}^{-1}$	GAL4 dimer binds to GAl1 gene
$D_{G1}{:}G_{4d} \longrightarrow D_{G1} + G_{4d}$	1	nucleoplasm	$6.410 \times 10^{-5}\mathrm{s}^{-1}$	GAL4 dimer dissociates from Gal1 gene
$D_{G1}{:}G_{4d} + G_{80d} \longrightarrow D_{G1}{:}G_{4d}{:}G_{80d}$	2	nucleoplasm	$3.583 \times 10^5 \mathrm{M}^{-1}\mathrm{s}^{-1}$	GAL80 dimer binds to GAl1 gene
$D_{G1}{:}G_{4d}{:}G_{80d} \longrightarrow D_{G1}{:}G_{4d} + G_{80d}$	1	nucleoplasm	$1.422 \times 10^{-3}\mathrm{s}^{-1}$	GAL80 dimer dissociates from Gal1 gene
$D_{G2} + G_{4d} \longrightarrow D_{G2}{:}G_{4d}$	2	nucleoplasm	$3.583 \times 10^7 \mathrm{M}^{-1}\mathrm{s}^{-1}$	GAL4 dimer binds to GAl2 gene
$D_{G2}{:}G_{4d} \longrightarrow D_{G2} + G_{4d}$	1	nucleoplasm	$1.684 \times 10^{-3}\mathrm{s}^{-1}$	GAL4 dimer dissociates from Gal2 gene
$D_{G2}{:}G_{4d} + G_{80d} \longrightarrow D_{G2}{:}G_{4d}{:}G_{80d}$	2	nucleoplasm	$3.583 \times 10^5 \mathrm{M}^{-1}\mathrm{s}^{-1}$	GAL80 dimer binds to GAl2 gene
$D_{G2}{:}G_{4d}{:}G_{80d} \longrightarrow D_{G2}{:}G_{4d} + G_{80d}$	1	nucleoplasm	$2.250 \times 10^{-3}\mathrm{s}^{-1}$	GAL80 dimer dissociates from Gal2 gene
$D_{G3} + G_{4d} \longrightarrow D_{G3}{:}G_{4d}$	2	nucleoplasm	$3.583 \times 10^7 \mathrm{M}^{-1}\mathrm{s}^{-1}$	GAL4 dimer binds to GAl3 gene
$D_{G3}{:}G_{4d} \longrightarrow D_{G3} + G_{4d}$	1	nucleoplasm	$6.720 \times 10^{-4}\mathrm{s}^{-1}$	GAL4 dimer dissociates from Gal3 gene
$D_{G3}{:}G_{4d} + G_{80d} \longrightarrow D_{G3}{:}G_{4d}{:}G_{80d}$	2	nucleoplasm	$3.583 \times 10^5 \mathrm{M}^{-1}\mathrm{s}^{-1}$	GAL80 dimer binds to GAl3 gene
$D_{G3}{:}G_{4d}{:}G_{80d} \longrightarrow D_{G3}{:}G_{4d} + G_{80d}$	1	nucleoplasm	$8.842 \times 10^{-3}\mathrm{s}^{-1}$	GAL80 dimer dissociates from Gal3 gene
$D_{G80} + G_{4d} \longrightarrow D_{G80}{:}G_{4d}$	2	nucleoplasm	$3.583 \times 10^7 \mathrm{M}^{-1}\mathrm{s}^{-1}$	GAL4 dimer binds to GAl80 gene
$D_{G80}{:}G_{4d} \longrightarrow D_{G80} + G_{4d}$	1	nucleoplasm	$6.720 \times 10^{-5}\mathrm{s}^{-1}$	GAL4 dimer dissociates from Gal80 gene

Chemical Reaction	Order	Region	Kinetic Parameters	Comments
$D_{G80}{:}G_{4d} + G_{80d} \longrightarrow D_{G80}{:}G_{4d}{:}G_{80d}$	2	nucleoplasm	$3.583 \times 10^{5}\mathrm{M}^{-1}\mathrm{s}^{-1}$	GAL80 dimer binds to GAl80 gene
$D_{G80}{:}G_{4d}{:}G_{80d} \longrightarrow D_{G80}{:}G_{4d} + G_{80d}$	1	nucleoplasm	$8.842 \times 10^{-3}\mathrm{s}^{-1}$	GAL80 dimer dissociates from Gal80 gene
$D_{G_{rep}} + G_{4d} \longrightarrow D_{G_{rep}}{:}G_{4d}$	2	nucleoplasm	$3.583 \times 10^{7}\mathrm{M}^{-1}\mathrm{s}^{-1}$	GAL4 dimer binds to GFP/YFP gene
$D_{G_{rep}}{:}G_{4d} \longrightarrow D_{G_{rep}} + G_{4d}$	1	nucleoplasm	$6.410 \times 10^{-5}\mathrm{s}^{-1}$	GAL4 dimer dissociates from GFP/YFP gene
$D_{G_{rep}}{:}G_{4d} + G_{80d} \longrightarrow D_{G_{rep}}{:}G_{4d}{:}G_{80d}$	2	nucleoplasm	$3.583 \times 10^{5}\mathrm{M}^{-1}\mathrm{s}^{-1}$	GAL80 dimer binds to GFP/YFP gene
$D_{G_{rep}}{:}G_{4d}{:}G_{80d} \longrightarrow D_{G_{rep}}{:}G_{4d} + G_{80d}$	1	nucleoplasm	$1.422 \times 10^{-3}\mathrm{s}^{-1}$	GAL80 dimer dissociates from GFP/YFP gene

Table H. Summary of transcription reactions in the system. Kinetic parameters are from the previous study [1]

Chemical Reaction	Order	Region	Kinetic Parameters	Comments
$D_{G_1}{:}G_{4d} \longrightarrow D_{G_1}{:}G_{4d} + R_1$	1	nucleoplasm	$0.012\mathrm{s}^{-1}$	GAL1 transcription
$D_{G_2}{:}G_{4d} \longrightarrow D_{G_2}{:}G_{4d} + R_2$	1	nucleoplasm	$0.042\mathrm{s}^{-1}$	GAL2 transcription
$D_{G_3}{:}G_{4d} \longrightarrow D_{G_3}{:}G_{4d} + R_3$	1	nucleoplasm	$7.110 \times 10^{-3}\mathrm{s}^{-1}$	GAL3 transcription
$D_{G_{rep}}{:}G_{4d} \longrightarrow D_{G_{rep}}{:}G_{4d} + R_{rep}$	1	nucleoplasm	$0.019\mathrm{s}^{-1}$	YFP/GFP transcription
$D_{G_{80}}{:}G_{4d} \longrightarrow D_{G_{80}}{:}G_{4d} + R_{80}$	1	nucleoplasm	$0.010\mathrm{s}^{-1}$	GAL80 transcription
$D_{G_4} \longrightarrow D_{G_4} + R_4$	1	nucleoplasm	$1.650 \times 10^{-4}\mathrm{s}^{-1}$	GAL4 transcription(no regulation)

Table I. Summary of mRNA degradation

Chemical Reaction	Order	Region	Kinetic Parameters	Comments
$R_1 \longrightarrow \varnothing$	1	cytoplasm; nucleoplasm; ribosomes	$3.727 \times 10^{-4}\mathrm{s}^{-1}$	R1 degradation
$R_2 \longrightarrow \varnothing$	1	cytoplasm; nucleoplasm; ribosomes	$1.284 \times 10^{-3}\mathrm{s}^{-1}$	R2 degradation

Chemical Reaction	Order	Region	Kinetic Parameters	Comments
$R_3 \longrightarrow \varnothing$	1	cytoplasm; nucleoplasm; ribosomes	$4.443 \times 10^{-4} \mathrm{s}^{-1}$	R3 degradation
$R_4 \longrightarrow \varnothing$	1	cytoplasm; nucleoplasm; ribosomes	$4.127 \times 10^{-4} \mathrm{s}^{-1}$	R4 degradation
$R_{80} \longrightarrow \varnothing$	1	cytoplasm; nucleoplasm; ribosomes	$4.813 \times 10^{-4} \mathrm{s}^{-1}$	R80 degradation
$R_{rep} \longrightarrow \varnothing$	1	cytoplasm; nucleoplasm; ribosomes	$5.777 \times 10^{-4} \mathrm{s}^{-1}$	GFP/YFP mRNA degradation

Table J. Summary of translation.

Chemical Reaction	Order	Region	Kinetic Parameters	Comments
Ribosome + $R_1 \longrightarrow$Ribosome:R_1	2	Ribosomes	$3.454 \times 10^5 \mathrm{M}^{-1}\mathrm{s}^{-1}$	R1 binds to Ribosome
Ribosome:$R_1 \longrightarrow$Ribosome+ $R_1 + G_1$	1	Ribosomes	$0.032 s^{-1}$	R1 translation
Ribosome + $R_2 \longrightarrow$Ribosome:R_2	2	Ribosomes	$3.454 \times 10^5 \mathrm{M}^{-1}\mathrm{s}^{-1}$	R2 binds to Ribosome
Ribosome:$R_2 \longrightarrow$Ribosome+ $R_2 + G_2$	1	Ribosomes	$0.225 s^{-1}$	R2 translation
Ribosome + $R_3 \longrightarrow$Ribosome:R_3	2	Ribosomes	$3.454 \times 10^5 \mathrm{M}^{-1}\mathrm{s}^{-1}$	R3 binds to Ribosome
Ribosome:$R_3 \longrightarrow$Ribosome+ $R_3 + G_3$	1	Ribosomes	$0.924 s^{-1}$	R3 translation
Ribosome + $R_4 \longrightarrow$Ribosome:R_4	2	Ribosomes	$3.454 \times 10^5 \mathrm{M}^{-1}\mathrm{s}^{-1}$	R4 binds to Ribosome
Ribosome:$R_4 \longrightarrow$Ribosome+ $R_4 + G_4$	1	Ribosomes	$0.178 s^{-1}$	R4 translation
Ribosome + $R_{rep} \longrightarrow$Ribosome:R_{rep}	2	Ribosomes	$3.454 \times 10^5 \mathrm{M}^{-1}\mathrm{s}^{-1}$	reporter mRNA binds to Ribosome
Ribosome:$R_{rep} \longrightarrow$Ribosome+ $R_{rep} + G_{rep}$	1	Ribosomes	$0.096 s^{-1}$	reporter mRNA translation
Ribosome + $R_{80} \longrightarrow$Ribosome:R_{80}	2	Ribosomes	$3.454 \times 10^5 \mathrm{M}^{-1}\mathrm{s}^{-1}$	R80 binds to Ribosome
Ribosome:$R_{80} \longrightarrow$Ribosome+ $R_{80} + G_{80}$	1	Ribosomes	$0.061 s^{-1}$	R80 translation

Table K. Summary of complex formation

Chemical Reaction	Order	Region	Kinetic Parameters	Comments
$G_4 + G_4 \longrightarrow G_{4d}$	2	cytoplasm; nucleoplasm	$3.583 \times 10^{10}\text{M}^{-1}\text{s}^{-1}$	GAL4 dimer formation
$G_{4d} \longrightarrow G_4 + G_4$	1	cytoplasm; nucleoplasm	$1.667 \times 10^{-5}\text{s}^{-1}$	GAL4 dimer dissociation
$G_{80} + G_{80} \longrightarrow G_{80d}$	2	cytoplasm; nucleoplasm	$3.583 \times 10^{10}\text{M}^{-1}\text{s}^{-1}$	GAL80 dimer formation
$G_{80d} \longrightarrow G_{80} + G_{80}$	1	cytoplasm; nucleoplasm	$1.667 \times 10^{-5}\text{s}^{-1}$	GAL80 dimer dissociation
$G_3 \longrightarrow G_{3i}$	1	cytoplasm	$266.940 \cdot [GAI]\text{s}^{-1}$	GAL3 binds to GAI
$G_{3i} \longrightarrow G_3$	1	cytoplasm	14.833s^{-1}	GAL3:GAI dissociation
$G_{3i} + G_{80d} \longrightarrow G_{80d}{:}G_{3i}$	2	cytoplasm	$9.214 \times 10^{6}\text{M}^{-1}\text{s}^{-1}$	GAL3:GAI binds to Gal80 dimer
$G_{80d}{:}G_{3i} \longrightarrow G_{3i} + G_{80d}$	1	cytoplasm	$2.660 \times 10^{-4}\text{s}^{-1}$	GAL3:GAI:G80d dissociation

Kinetic parameters are adopted from previously published studies [3].

Table L. Summary of protein/complex degradation

Chemical Reaction	Order	Region	Kinetic Parameters	Comments
$G_{3i} \longrightarrow \varnothing$	1	cytoplasm	$1.925 \times 10^{-4}\text{s}^{-1}$	GAL3 degradation
$G_{80D}{:}G_{3i} \longrightarrow \varnothing$	1	cytoplasm	$9.625 \times 10^{-5}\text{s}^{-1}$	Gal3:GAI:Gal80 degradation
$G_1 \longrightarrow \varnothing$	1	cytoplasm	$6.418 \times 10^{-5}\text{s}^{-1}$	G1 degradation
$G_2 \longrightarrow \varnothing$	1	cytoplasm plasmaMembrane	$6.418 \times 10^{-5}\text{s}^{-1}$	Gal2 degradation
$G_3 \longrightarrow \varnothing$	1	cytoplasm	$1.925 \times 10^{-4}\text{s}^{-1}$	Gal3 degradation
$G_4 \longrightarrow \varnothing$	1	cytoplasm; nucleoplasm	$1.155 \times 10^{-4}\text{s}^{-1}$	Gal4 degradation
$G_{rep} \longrightarrow \varnothing$	1	cytoplasm	$1.925 \times 10^{-4}\text{s}^{-1}$	Reporter degradation
$G_{80} \longrightarrow \varnothing$	1	cytoplasm; nucleoplasm	$1.155 \times 10^{-4}\text{s}^{-1}$	Gal80 degradation

1.5 Chemical reactions in the system

Table M. Summary of galactose transportation and metabolism in ODE

Chemical Reaction	Order	Kinetic Parameters	Comments
$G_1 + GAI \longrightarrow G_1GAI$	2	$1.442 \times 10^5 \mathrm{M}^{-1}\mathrm{s}^{-1}$	G1 bind to galactokinase
$G_1GAI \longrightarrow G_1 + GAI$	1	$30.708\mathrm{s}^{-1}$	
$G_1GAI \longrightarrow G_1$	1	$55.833\mathrm{s}^{-1}$	metabolite of Galactose
$G_2 \longrightarrow G2GAE$	1	$1.123 \times 10^5 \cdot [GAE]\mathrm{s}^{-1}$	transporter bind to external galactose
$G_2GAE \longrightarrow G_2$	1	$39.875\mathrm{s}^{-1}$	
$G_2GAE \longrightarrow G_2GAI$	1	$72.5\mathrm{s}^{-1}$	symmetric transport
$G_2GAI \longrightarrow G_2GAE$	1	$72.5\mathrm{s}^{-1}$	symmetric transport
$G_2GAI \longrightarrow G_2 + GAI$	1	$39.875\mathrm{s}^{-1}$	release galactose to cytoplasm

Kinetic parameters are adopted from previously published studies [1].

1.6 Diffusion Coefficients for species in RDME and ODE

The diffusion coefficients for all species in the RDME simulation. If the diffusion rule is not mentioned, then it is not allowed between regions.

Table N. Diffusion Coefficients for Various Species between different Regions.

Species	From	To	Diffusion Coefficient (m^2/s)
genes	Any	Any	0
mRNAs	nucleoplasm	nucleoplasm	0.05×10^{-12}
	nucleoplasm	cytoplasm	0.05×10^{-12}
	cytoplasm	cytoplasm	0.05×10^{-12}
	cytoplasm	ribosome	0.05×10^{-12}
	ribosome	cytoplasm	0.05×10^{-12}
proteins and complexes	ribosome	cytoplasm	2.74×10^{-12}
	cytoplasm	cytoplasm	1.00×10^{-12}
	ribosome	ER	2.74×10^{-12}
G4/G4d/G80/G80d	nucleoplasm	nucleoplasm	1.00×10^{-12}
	nucleoplasm	cytoplasm	1.00×10^{-12}
	cytoplasm	nucleoplasm	1.00×10^{-12}
G2	cytoplasm	plasma membrane	1.00×10^{-12}
	plasma membrane	plasma membrane	0.01×10^{-12}
	ER	ER	1×10^{-12}
	ER	cytoplasm	1×10^{-12}

Diffusion Coefficients are adopted from previously published studies [2, 4].

2 Geometry Reconstruction

We demonstrate the process of reconstructing of nuclear envelope as an example:

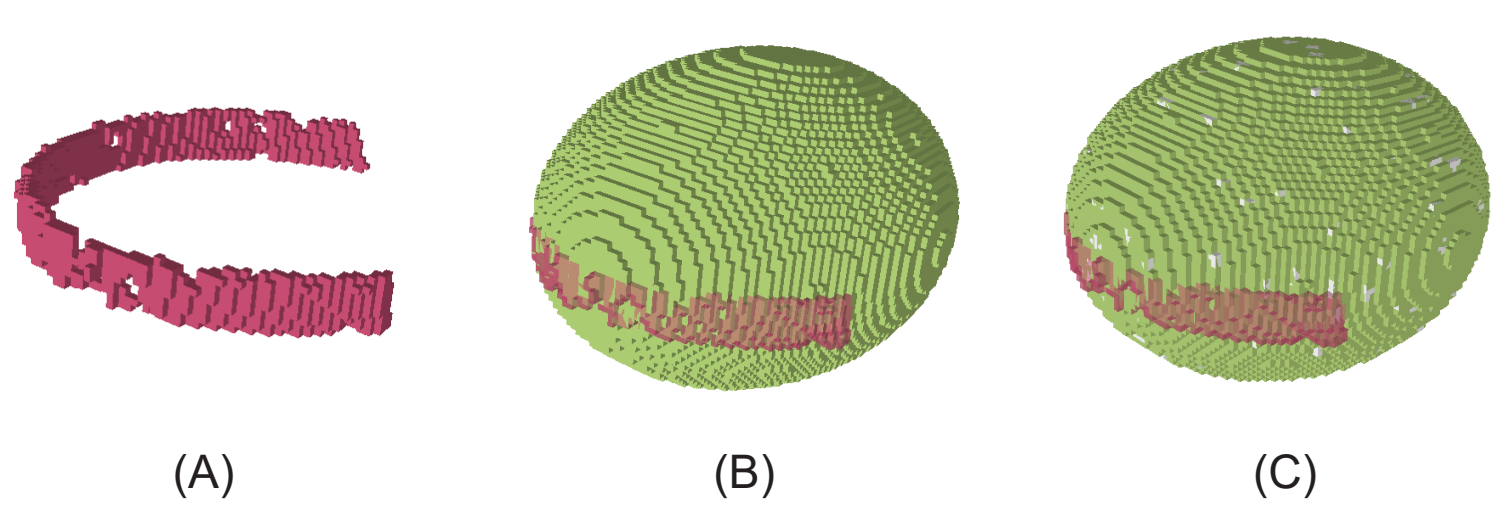

Fig A. Geometric Reconstruction of the Nuclear Envelope.(A) Segmentation of the nuclear envelope membranes included in cryo-ET data of the yeast cell slice.(B) Ellipsoidal fitting of the nuclear envelope.(C) Addition of nuclear pore complexes as white crosses to the reconstructed envelope.

2.1 Geometric Reconstructions Parameters

Here, we assumed plasma membrane, nucleus envelope and cell wall are ellipsoidal structures and used python *scipy* to fit the parameters. All fitted parameters are listed below:

Table O. Ellipsoid parameters: center coordinates, Euler orientations, and semi-axis lengths. Length unit: lattice coordinates; Angle unit: radians

**Structure**	**Center** (x, y, z)	**Orientation** (α, β, γ)	**Semi-axes** (a, b, c)
Plasma Membrane	(96, 96, 96)	(3.39, −1.59, −0.90)	(61, 92, 61)
Nuclear Envelope	(132, 81, 112)	(0.16, −1.74, 0.56)	(30, 37, 30)
Cell Wall	(96, 96, 96)	(3.39, −1.59, −0.90)	(65, 96, 65)

Because the mitochondria and vacuole are randomly positioned within the cytoplasmic region with fixed volumetric occupancy, each execution of the script generates distinct mitochondrial and vacuolar configurations with varying parameters. Consequently, these values are not reported in the table.

2.2 Chromosome model

We utilized the static chromosome model [5], applying spatial transformations and uniform scaling to maximize nucleoplasmic occupancy while preventing overlap with the nuclear envelope.

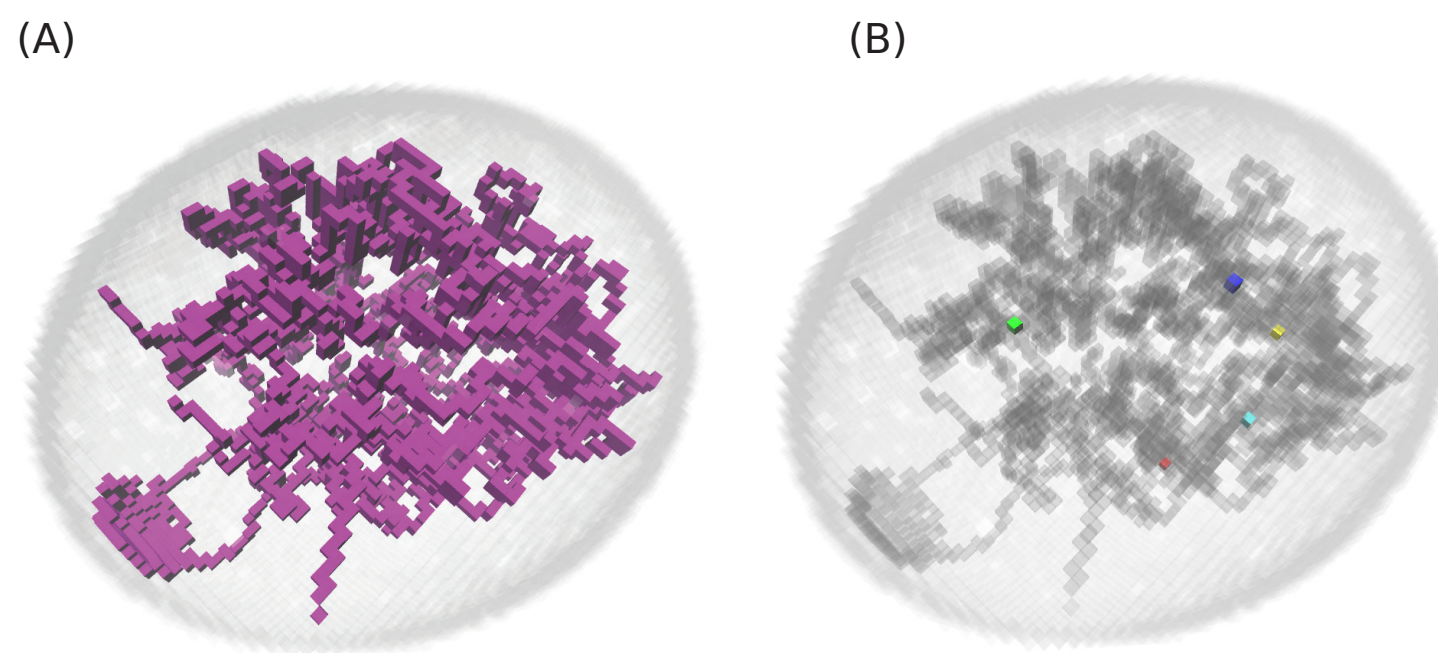

Fig B. (A)Chromosomes in the nuclear envelope. (B) Gene locations in the chromosome. GAL1: cyan, GAL2: red, GAL3: yellow, GAL4: green, GAL80: blue.

Gene	1-step	2-step	3-step
GAL1	23.1%	18.4%	8.3%
GAL2	23.1%	11.2%	6.9%
GAL3	0.0%	3.1%	10.6%
GAL4	0.0%	8.2%	8.3%
GAL80	0.0%	14.3%	10.1%

Table P. Neighbor occupancy by chromosome lattice.

2.3 Nuclear Pore

We measured the surface density of nuclear pore complex to be $13.21/\mu m^2$ based on the cryo-ET data [2]. Then, we assumed the nuclear pores are uniformly distributed on the entire nuclear envelope which make the total number of 139.

3 Additional Simulation Results

3.1 Additional *GAL*-related species for Section 3.1

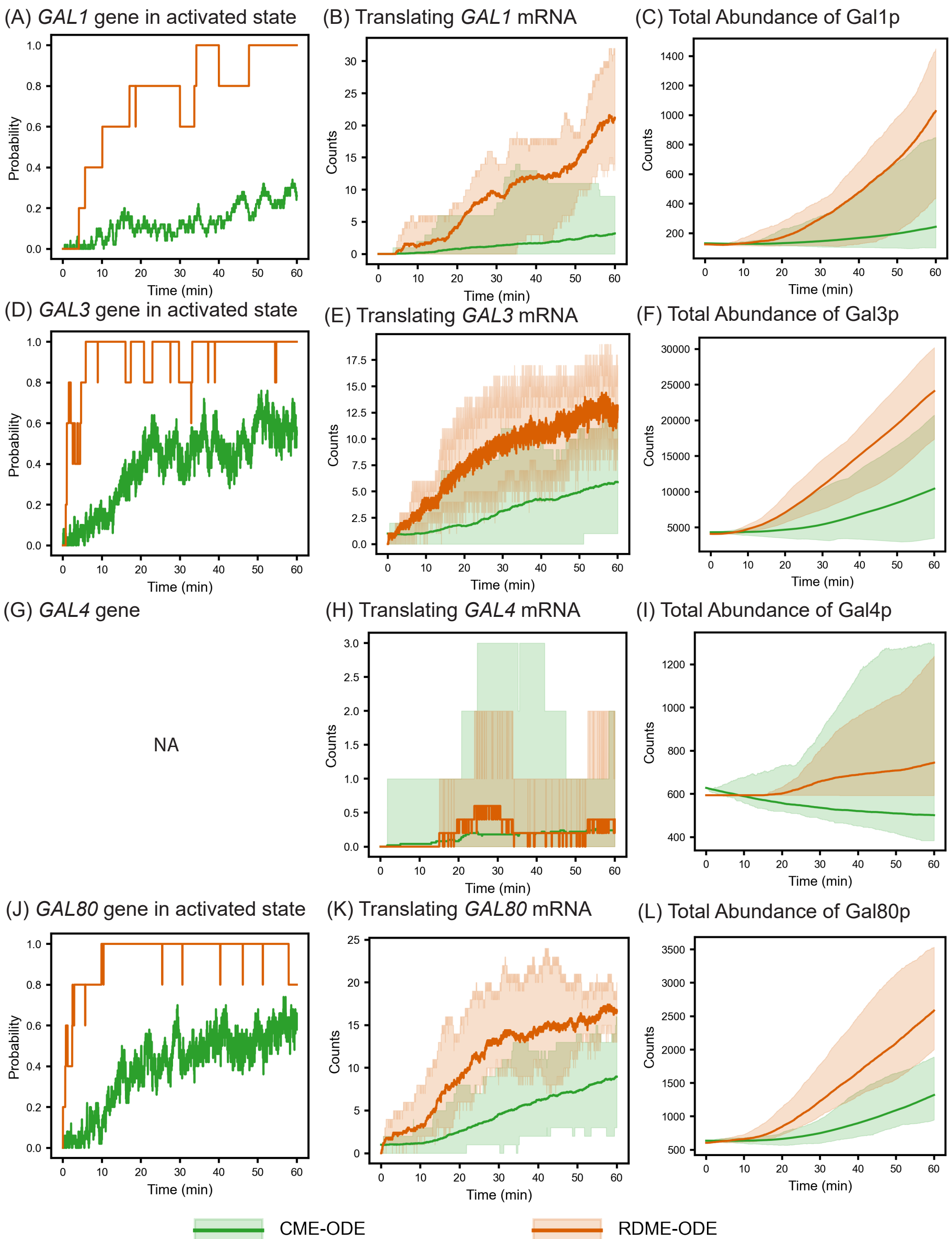

Fig C

The Gal4 gene trajectory (Figure CG) is not shown because Gal4 transcription is defined as $\varnothing \longrightarrow$ mRNA, meaning that no explicit *GAL4* gene species exists in the CME model.

3.2 Additional *GAL*-related species for Section 3.2

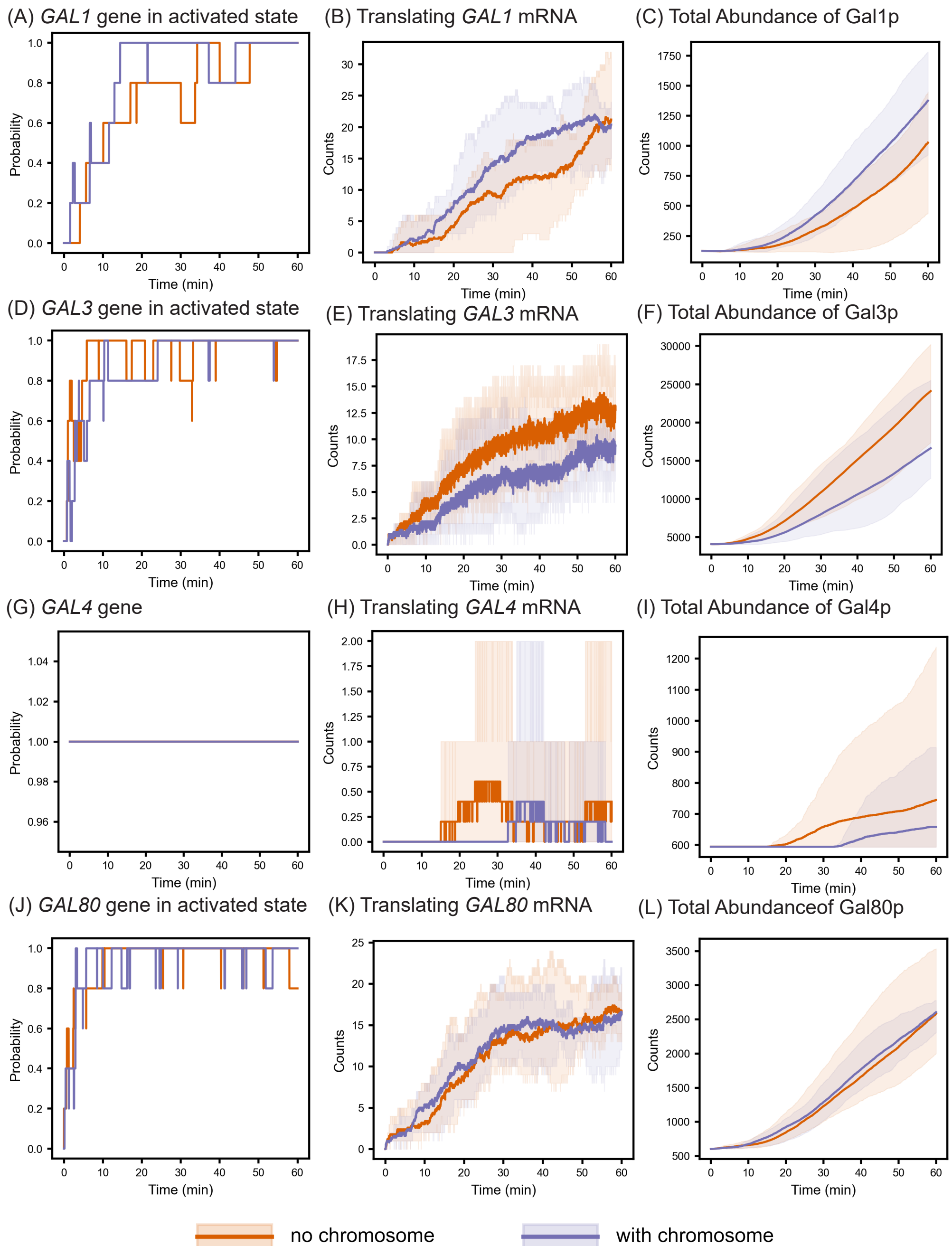

Fig D

3.3 Additional *GAL*-related species for Section 3.3

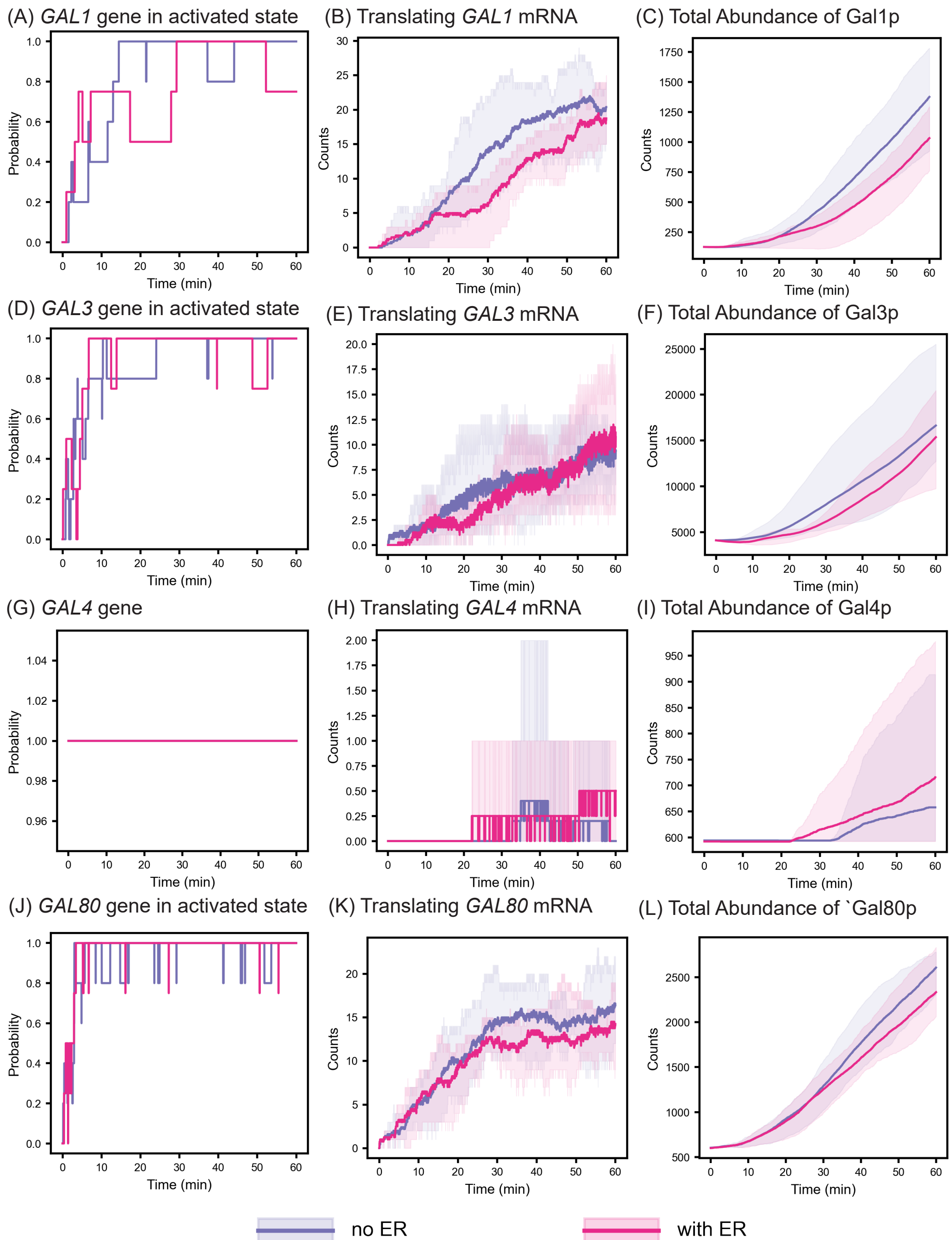

Fig E

3.4 Additional *GAL*-related species for Section 3.4

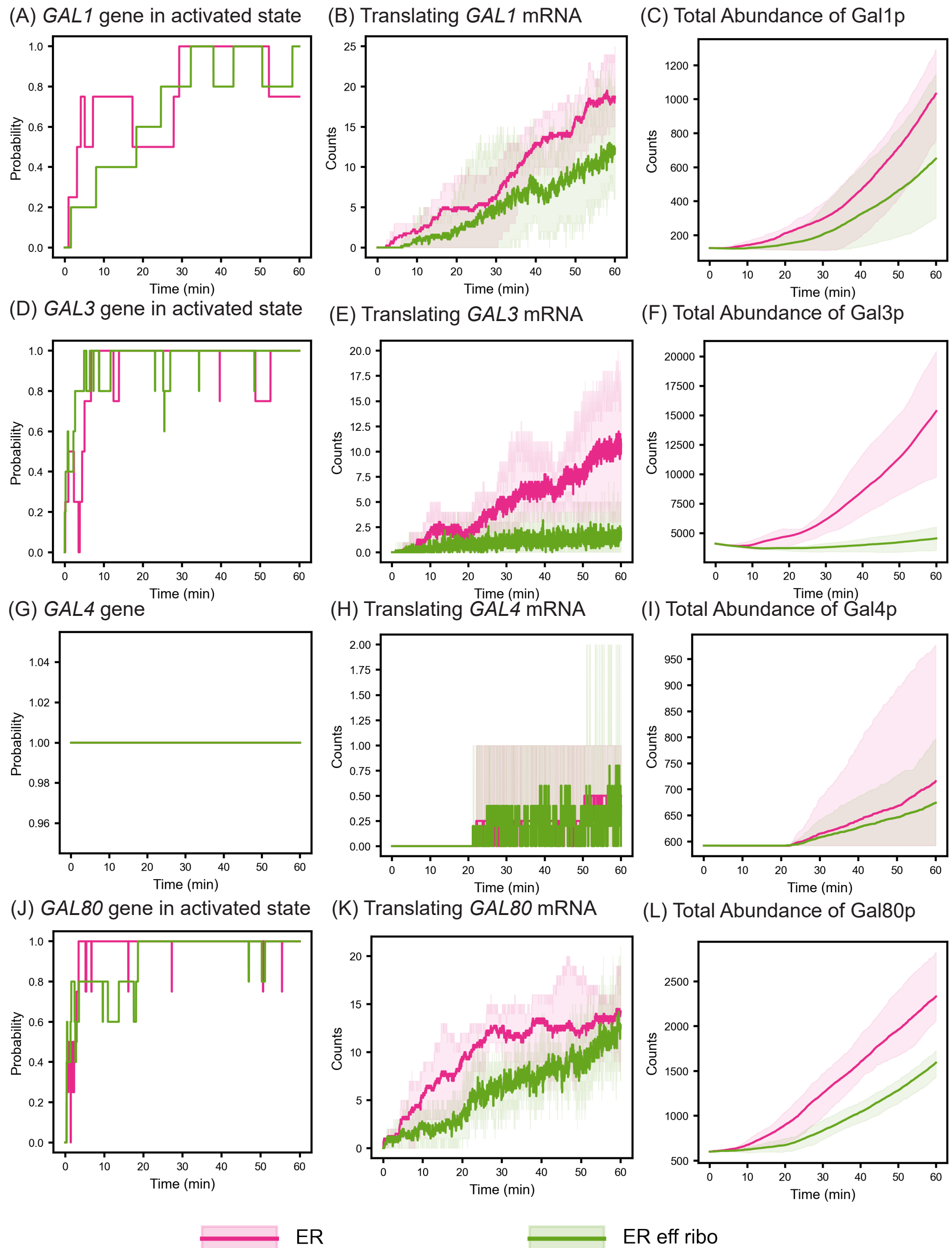

Fig F

As shown in Fig. FF, the induction effect of Gal3p appears weaker than that of the other *GAL* proteins in the effective-ribosome simulations. This reduced induction primarily arises from the high initial Gal3p abundance used in the model, which was at least fourfold higher than that of the other *GAL* genes. The initial abundance (4,341 molecules) was adopted from Ramsey *et al.* [3] and also applied in the CME study by Bianchi *et al.* [1].

To evaluate the effect of a more physiologically relevant starting level, we estimated the initial Gal3p abundance based on the mean value reported in the Saccharomyces Genome Database (SGD) [6], which is 865 molecules, and scaled it according to proteomics data for cells grown in 2% raffinose medium [7]. Applying the reported ratio (65%), the adjusted initial abundance is $0.65 \times 865 = 564$. When this revised abundance is used, the induction response of Gal3p becomes comparable to that of the other *GAL* proteins, as shown in Fig. GB.

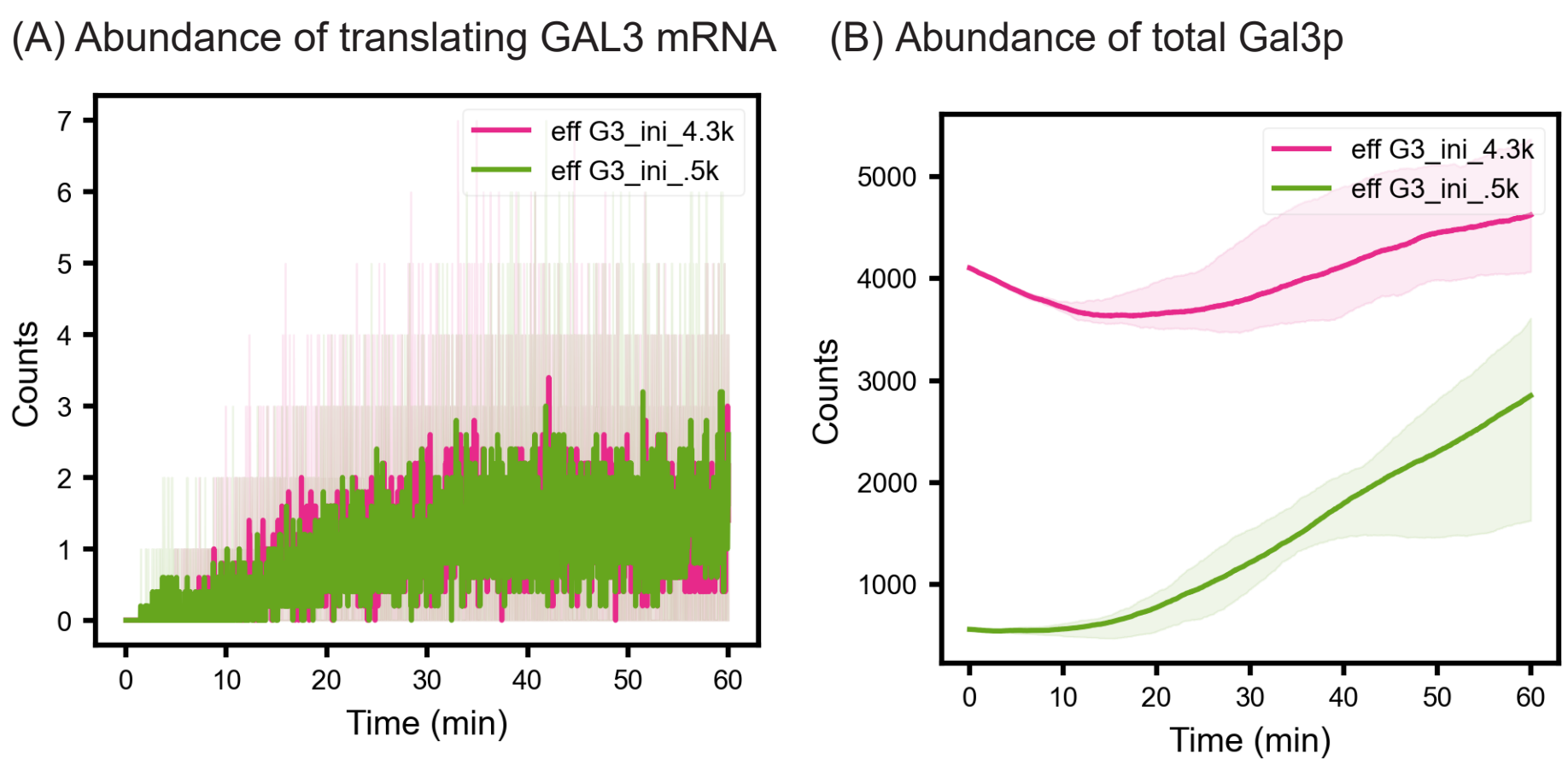

Fig G

At the same time, we note that the initially higher fraction of translating *GAL2* mRNA(Main Text, Fig. 9A) arises from its relatively low total mRNA abundance at the start of the simulation. Moreover, no significant differences were observed within the first 10 minutes in either total *GAL2* mRNA or translating *GAL2* mRNA levels ($p < 0.05$; FigH).

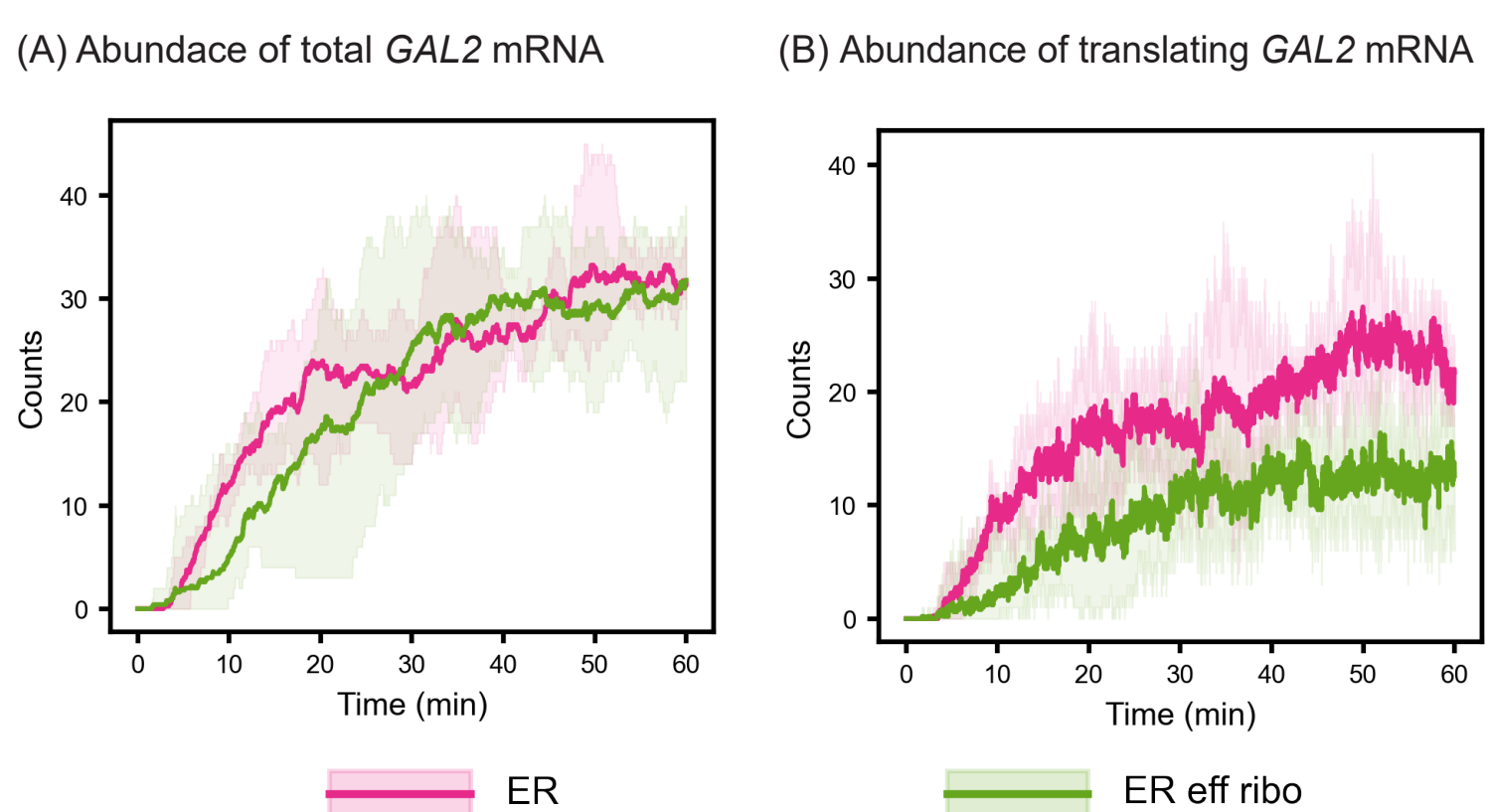

Fig H

3.5 Ribosome in Translation

The translating ribosome trajectories for no-ER, with-ER and no-ER 2,102 ribosome cases respectively. It is clear, the maximum translating ribosomes are below 150 in first 60 minutes simulation in all cases.

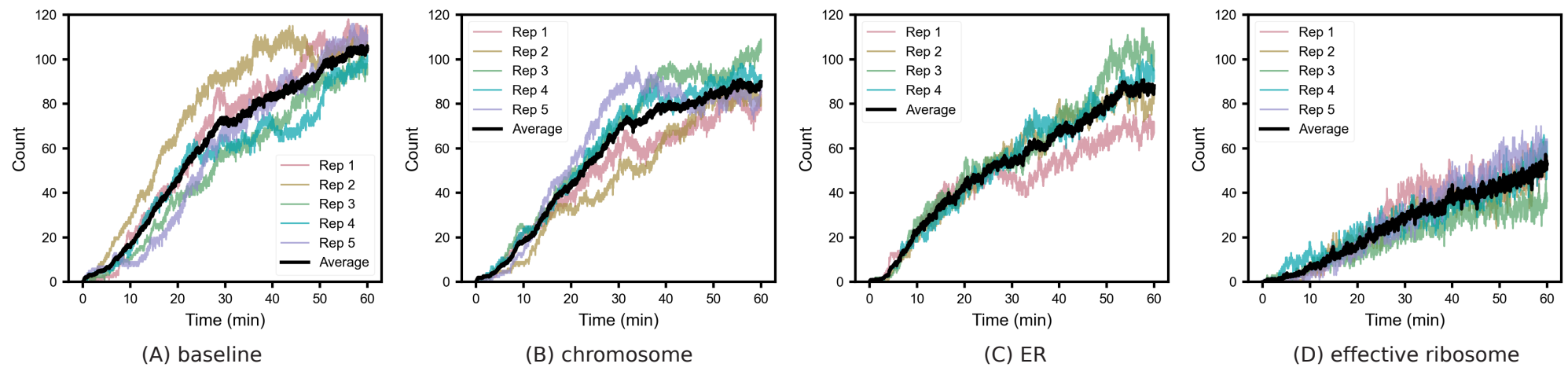

Fig I. (A) Yeast cell in 60 minutes simulation.(B) Yeast cell in 60 minutes simulation with chromosomes only. (C) Yeast cell in 60 minutes simulation with ER and chromosomes. (D) Yeast cell in 60 minutes simulation with effective ribosomes, ER and chromosomes.

3.6 Comparison of Repressor Dynamics in the Nucleoplasm and Cytoplasm Between RDME-ODE and CME-ODE Models

Figure J compares the time-resolved dynamics of the repressor G80d in the nucleoplasm (A) and cytoplasm (B) between the well-stirred CME–ODE and spatially resolved RDME–ODE frameworks. In both compartments, the repressor G80d exhibits a rapid transient decline over the first ∼10 minutes, followed by relaxation toward a low steady-state abundance for the remainder of the 60-minute simulation window. While the qualitative temporal trend is conserved across modeling frameworks, the spatial RDME–ODE simulations maintain consistently lower repressor copy numbers at early times, most prominently in the nucleoplasm. This early depletion is accompanied by reduced variability in G80d abundance in RDME–ODE compared with CME–ODE, consistent with spatial compartmentalization and diffusion constraints that redistribute repressors across subcellular volumes. Importantly, after the initial transient, both models converge to comparably low nucleoplasmic and cytoplasmic G80d levels, indicating that long-timescale repressor availability becomes similar across frameworks despite differences in early-time spatial redistribution.

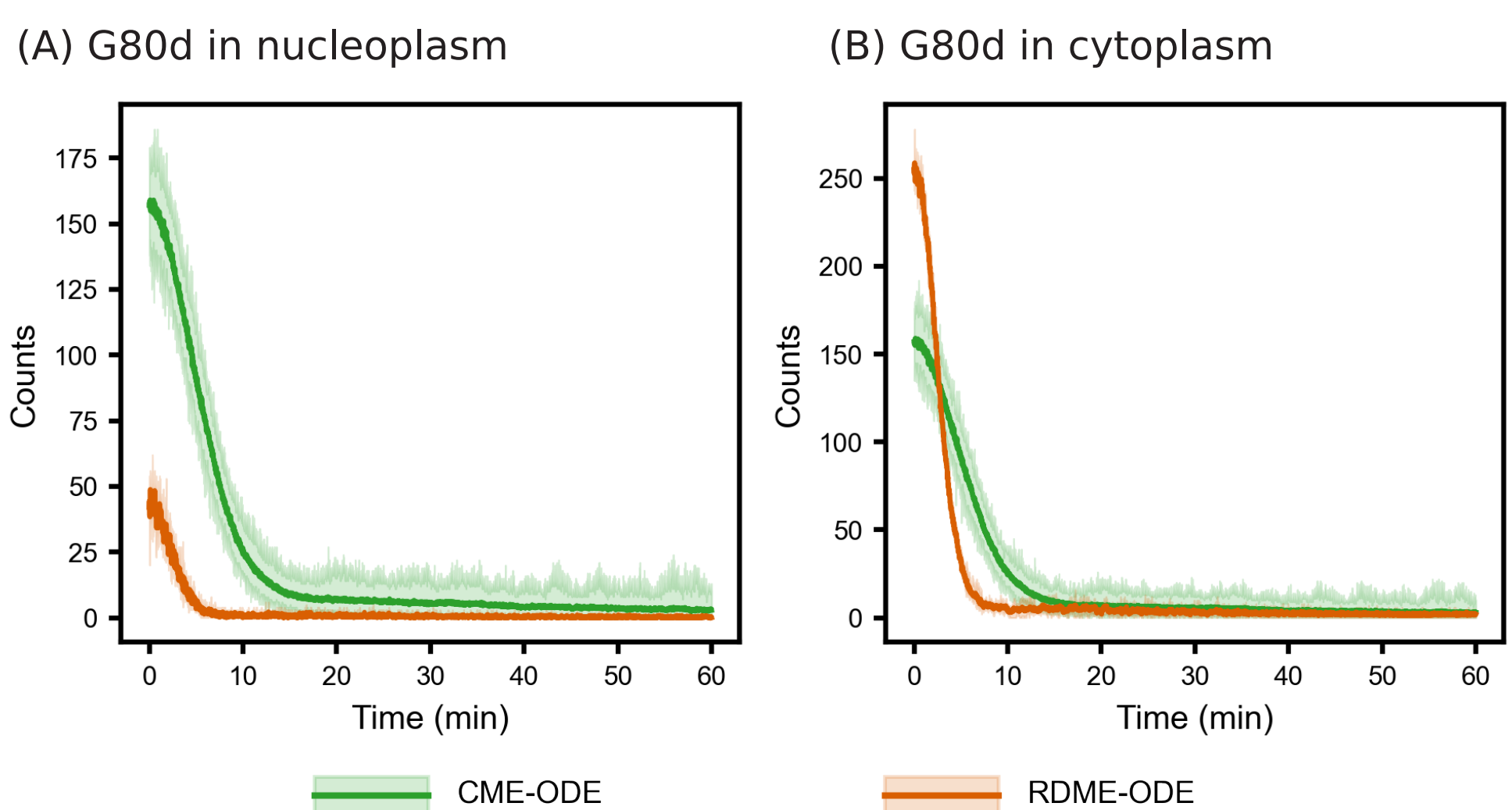

Fig J. (A)G80d abundance in nucleoplasm. (B)G80d abundance in cytoplasm.

3.7 Comparison of Activator and Repressor Dynamics in the Nucleoplasm and Cytoplasm Between Non-Chromosome and Chromosome-Included Models

Figure K compares the nucleoplasmic dynamics of the activator Gal4p dimer (G4d; panel A) and the repressor Gal80p dimer (G80d; panel B) between spatial RDME–ODE simulations performed with and without explicit chromosome geometries. Across the full 60-minute simulation window, G4d copy numbers remain relatively stable and exhibit substantial overlap between the two conditions, indicating that inclusion of chromosome occupancy does not measurably perturb the nucleoplasmic abundance of the core activator. Similarly, G80d displays a rapid early-time depletion over the first ∼10 minutes, followed by convergence to near-zero abundance thereafter; importantly, this transient decay profile is nearly identical between chromosome and non-chromosome simulations. Together, these results suggest that introducing chromosome lattice obstacles does not significantly alter the steady-state or transient nucleoplasmic availability of either regulator, consistent with a minimal impact of chromosome volume exclusion on regulator diffusion and promoter accessibility at the occupied volume fractions present in the model.

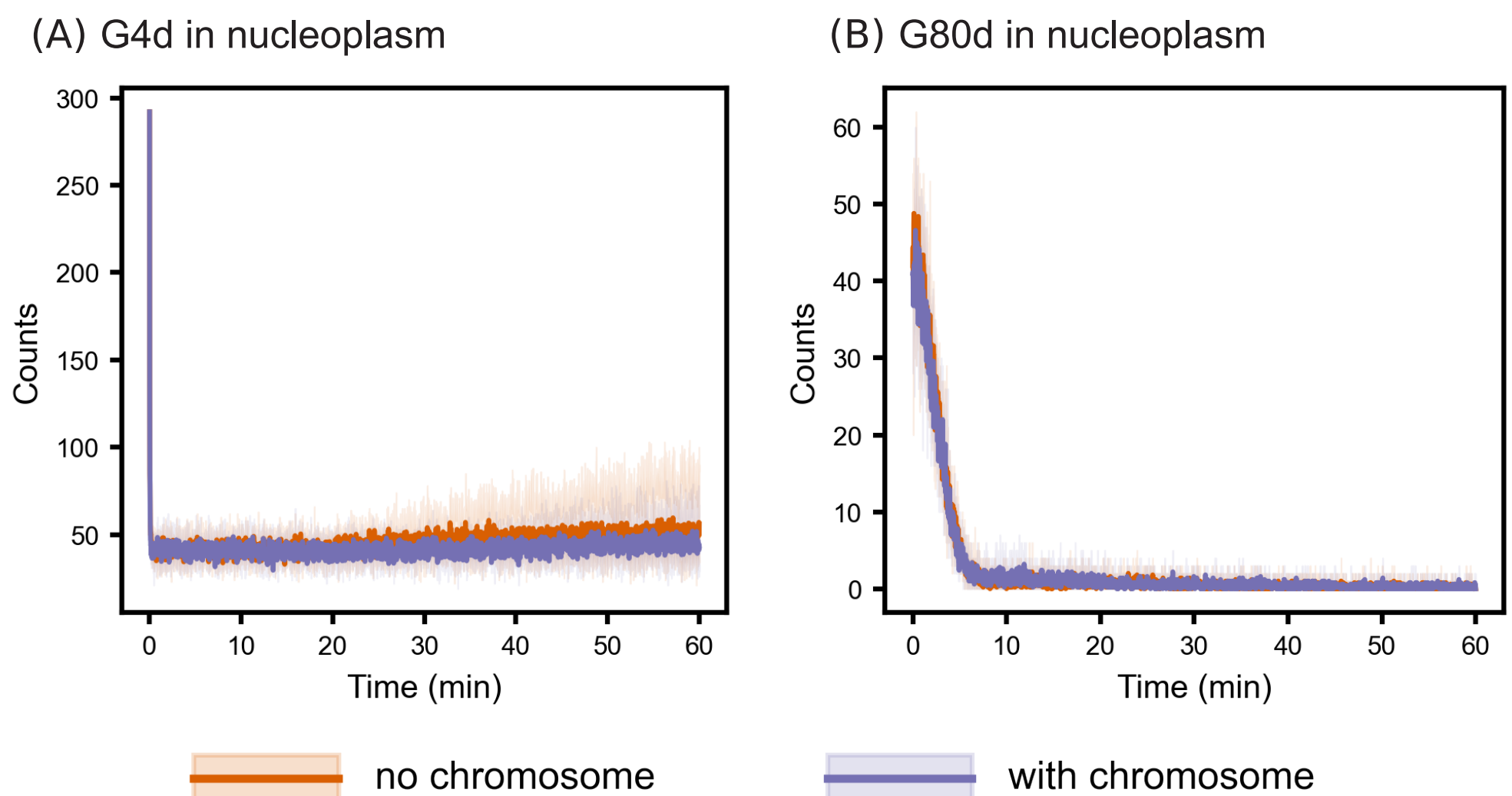

Fig K. (A)G80d abundance in nucleoplasm. (B)G80d abundance in cytoplasm.

3.8 Gene Positioning in the Nucleus Has Minimal Impact on Activation Dynamics

We next examined whether the spatial placement of *GAL* genes within the nucleus can influence induction dynamics in the RDME–ODE framework. To isolate gene-position effects from chromosome volume exclusion, we performed additional simulations using the RDME–ODE model with all chromosome lattice sites removed, yielding a homogeneous nucleoplasmic volume. Within this non-chromosome setting, we tested two extreme gene localization scenarios: (i) all *GAL* network genes placed at the geometric center of the nucleoplasm and (ii) all *GAL* genes placed near the nuclear periphery at randomly selected edge voxels. Across these conditions, we observed no significant differences ($p > 0.05$) in either the timing or magnitude of gene activation, indicating that gene positioning alone does not measurably affect induction under the present parameter regime. For clarity, the plots show only transporter-related species.

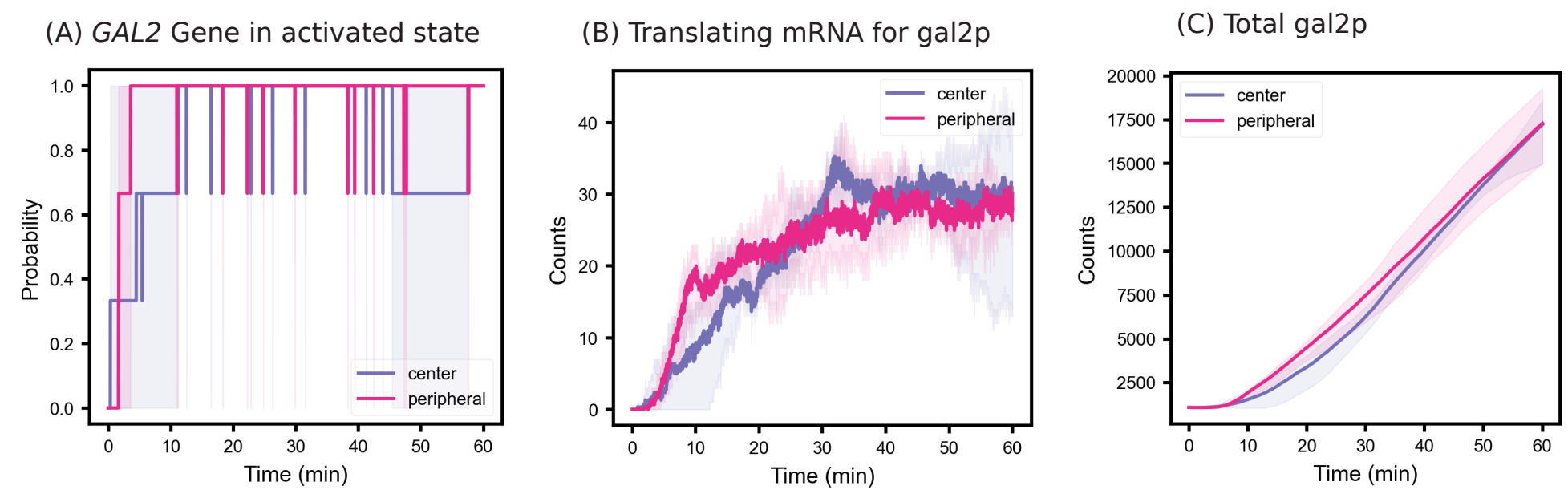

Fig L. RDME-ODE simulations with gene at the center and the peripheral of nucleoplasm.

3.9 Ribosome Number and Spatial Organization Jointly Limit GAL2 Translation

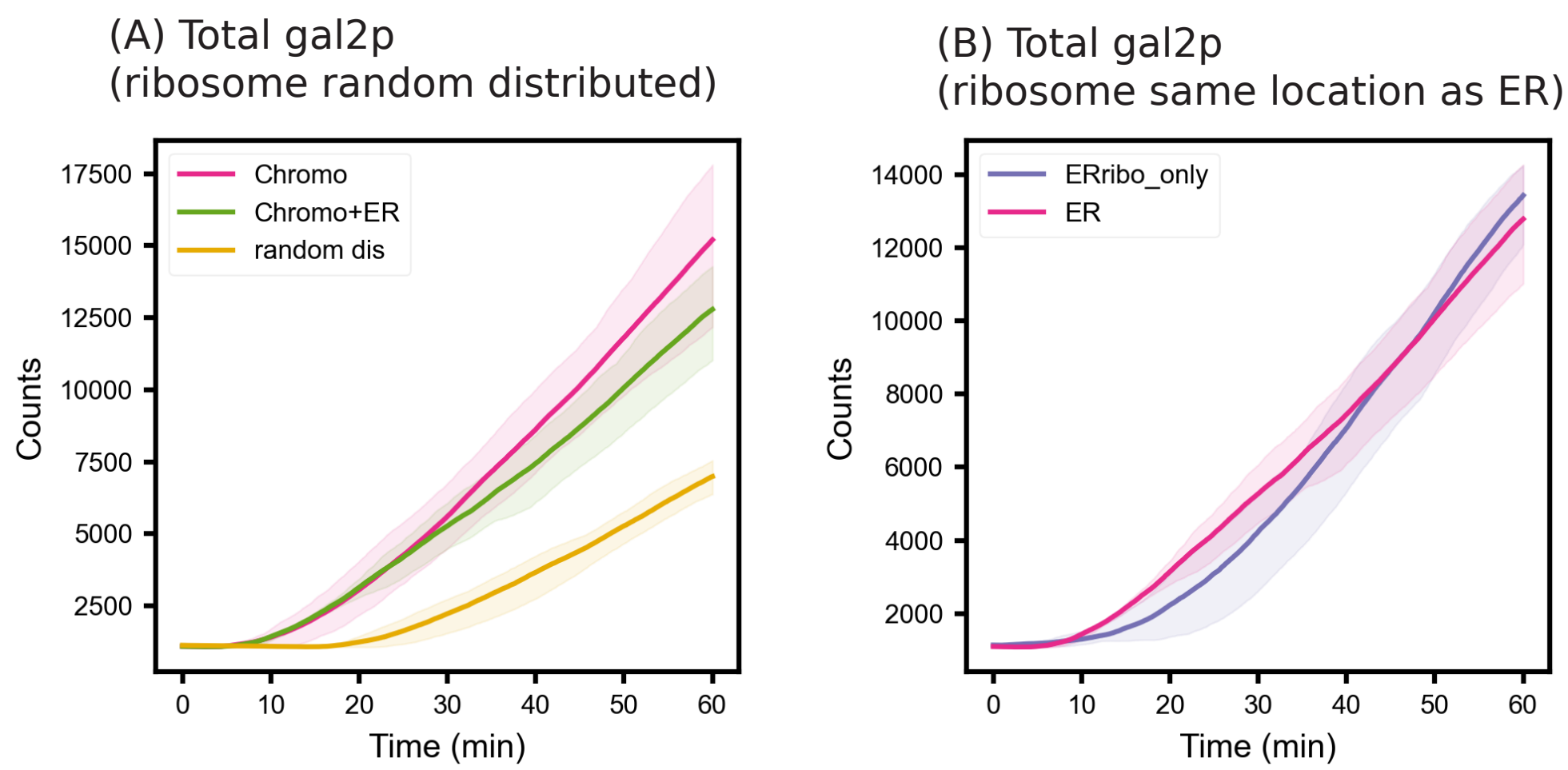

Fig M. Numerical and spatial ribosome distributions jointly shape *GAL2* expression dynamics.(A) Comparison of total gal2p abundance in chromosome (no ER), ER (with ER) and randomly distributed simulation. (B) Comparison of total gal2p in ERRibo_only (ER case with ER geometry removed and keep ER-associated ribosomes in their location) and ER ("with ER" case in main text).

3.10 Ablation analysis of factors contributing to CME–ODE and RDME–ODE differences

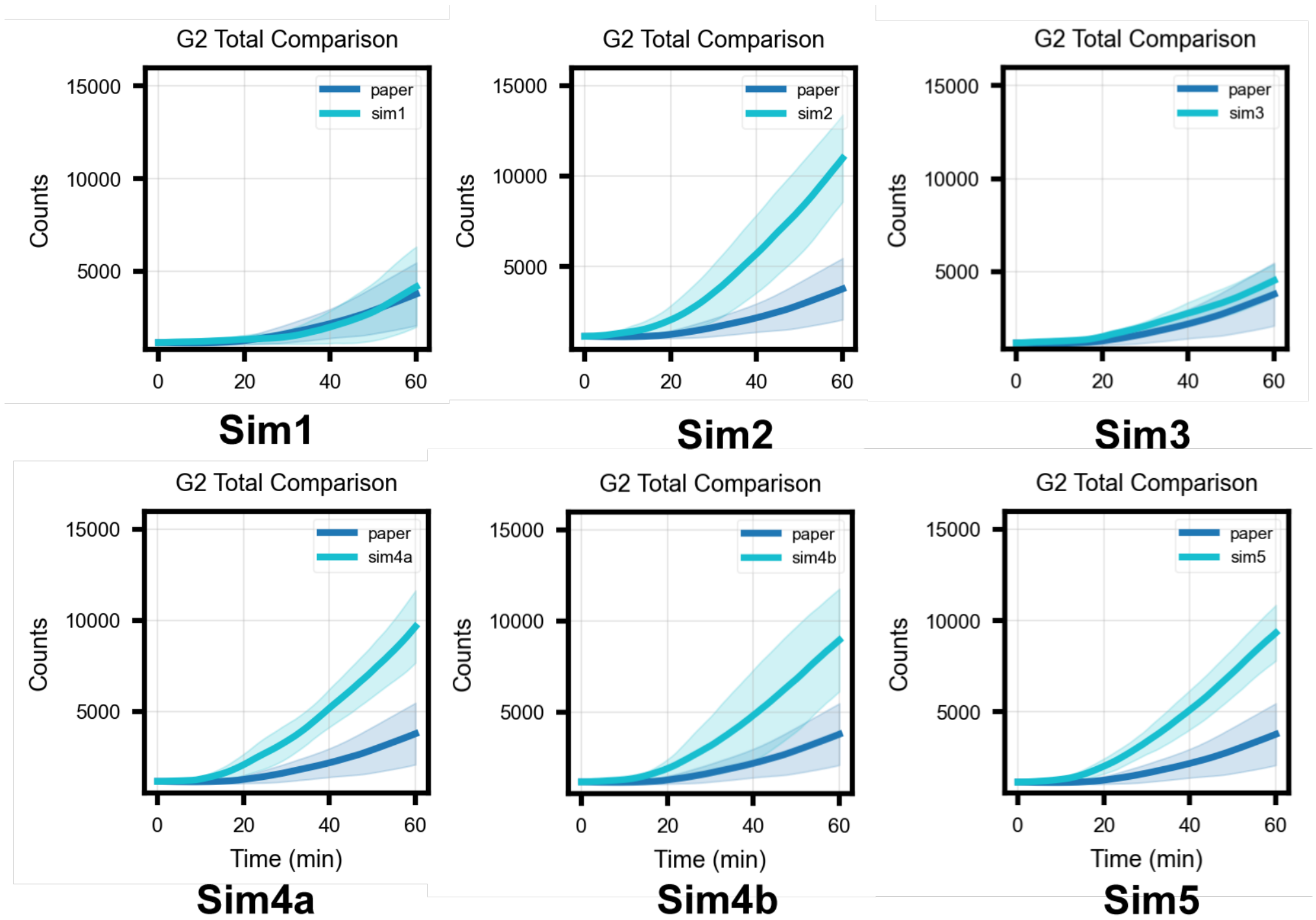

Fig N. Ablation analysis of factors contributing to differences between CME–ODE and RDME–ODE simulations. Six simulation conditions were used to test candidate mechanisms underlying the difference in G2 abundance between the CME–ODE and RDME–ODE results in Fig. 5C. All ablation cases were simulated in the CME–ODE framework with 10 replicate trajectories per condition. Sim1 modifies the initial G80d distribution from a uniform distribution to a distribution proportional to the nuclear and cytoplasmic volume ratio used in the RDME–ODE simulation. Sim2 reduces the promoter-binding kinetic parameter to account for the approximately 16-s diffusion-limited promoter search time observed in the RDME–ODE simulation. Sim3 tests local-volume effects by scaling both binding and unbinding rates fivefold while preserving the equilibrium constant. Sim4a increases cytoplasm-to-nucleus transport sevenfold, whereas Sim4b decreases nucleus-to-cytoplasm transport sevenfold, testing the effect of asymmetric nuclear-pore transport. Sim5 combines the contributing perturbations to test whether their effects are additive. Together, these simulations show that the discrepancy is not primarily explained by initial G80d partitioning, but instead reflects spatial effects that modify effective reaction kinetics, including diffusion-limited promoter search, local-volume-dependent encounter rates, and asymmetric nuclear transport.

To identify factors contributing to the difference between the CME–ODE and RDME–ODE simulations in Fig. 5C, we performed five additional ablation-like simulation cases in which one modeling feature was perturbed at a time. All additional simulations were performed in the CME–ODE framework, with 10 replicate trajectories for each case. These simulations were designed to test whether the discrepancy between the two frameworks could be explained by initial compartmental partitioning, diffusion-limited promoter search, local-volume effects, asymmetric nuclear transport, or

interactions among these effects.

In Sim1, we changed the initial distribution of G80d from a uniform distribution to a distribution proportional to the nuclear and cytoplasmic volume ratio used in the RDME–ODE simulation. This perturbation produced no significant change in G2 abundance over the 60-min simulation, suggesting that the initial G80d partitioning alone is not the major source of the discrepancy between the CME–ODE and RDME–ODE results.

In Sim2, we reduced the promoter-binding kinetic parameter to account for the approximately 16-s search time required for the promoter to leave and re-enter the promoter region in the nucleus in the RDME–ODE simulation. This perturbation produced a substantial change in G2 abundance, indicating that diffusion-limited promoter search can strongly affect the effective kinetic behavior. This result suggests that, if a simpler and faster CME–ODE description is used, its kinetic parameters may need to be re-parameterized to account for diffusion-mediated delays.

In Sim3, we tested whether local-volume effects could explain the observed discrepancy. To mimic a fivefold reduction in effective local volume, we scaled both the binding and unbinding rates by fivefold while keeping the equilibrium constant unchanged. Thus, binding and unbinding occurred five times faster, while the equilibrium affinity was preserved. Under this condition, G2 abundance increased, suggesting that local-volume-dependent changes in encounter frequency can alter the predicted regulatory response even when the equilibrium binding affinity is unchanged.

In Sim4, we examined the effect of asymmetric diffusion through nuclear pores. Based on the calculated directional transport ratio, we either increased cytoplasm-to-nucleus diffusion by sevenfold (Sim4a) or decreased nucleus-to-cytoplasm diffusion by sevenfold (Sim4b). Both perturbations increased G2 abundance, indicating that asymmetric nuclear transport may also contribute to the differences between the CME–ODE and RDME–ODE simulations.

Finally, in Sim5, we combined the perturbations identified above as contributing factors. The combined perturbation did not produce a simple additive increase in G2 abundance. Instead, the response appeared to approach an upper bound, suggesting that these mechanisms interact nonlinearly rather than contributing independently. Together, these ablation simulations indicate that the CME–ODE/RDME–ODE discrepancy is not caused by a single initialization choice, but instead arises primarily from spatial effects that alter effective reaction kinetics, including diffusion-limited promoter search, local-volume effects, and asymmetric nuclear transport.

These results support the interpretation discussed in Section 4.1 of the main text. They also suggest a practical route for using simpler CME–ODE models when full spatial RDME–ODE simulations are computationally inaccessible: the CME–ODE kinetic parameters should be re-parameterized to incorporate diffusion-dependent effective rates.

3.11 Obstacle perturbation analysis of local GAL2 promoter accessibility

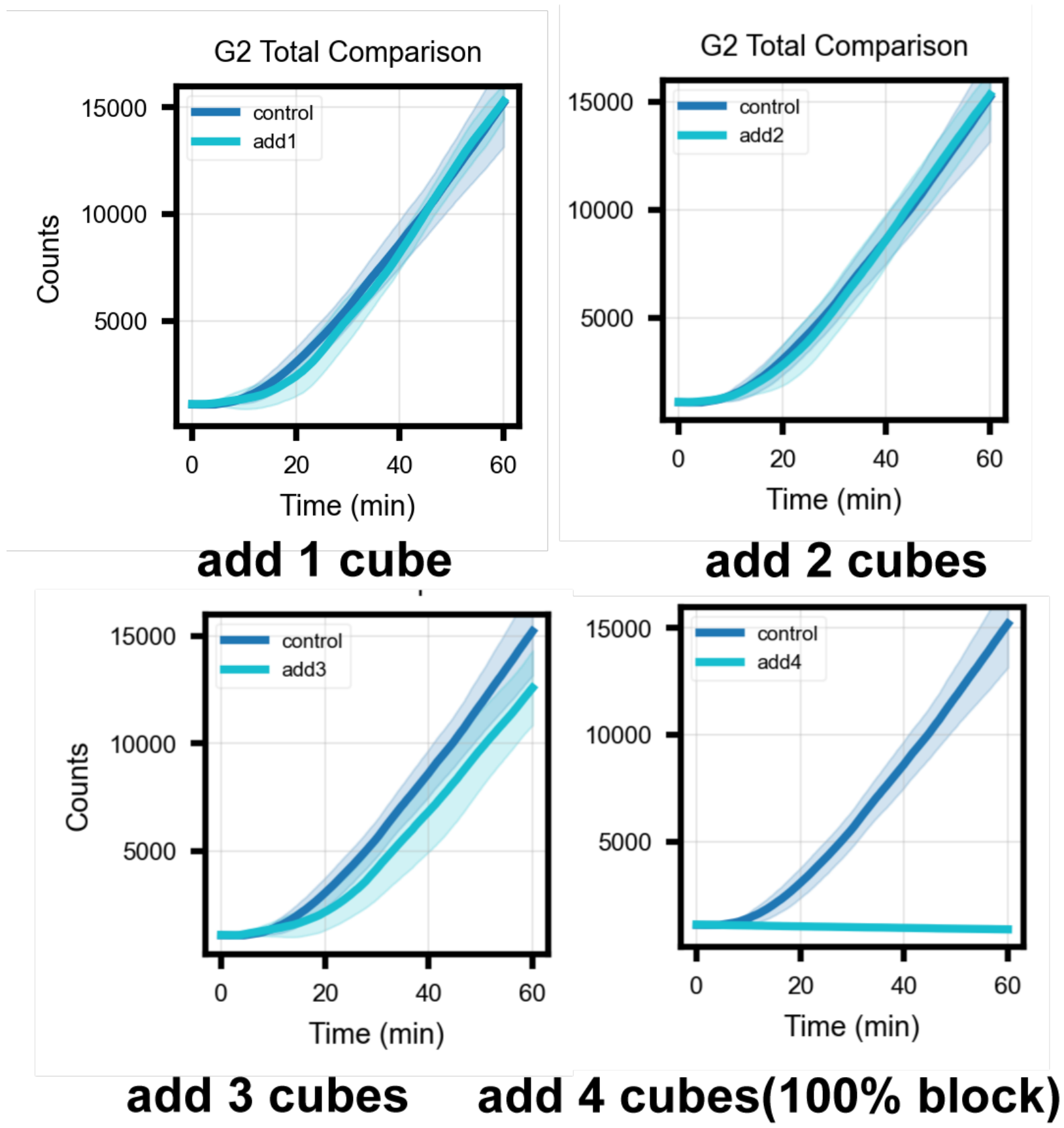

Fig O. Obstacle perturbation analysis of local *GAL2* promoter accessibility.

To further assess how physical obstacles affect gene-locus accessibility, we artificially introduced additional blocked lattice sites around the *GAL2* gene to mimic increased local chromatin crowding. In Lattice Microbes, diffusion events can occur across the six directly neighboring lattice sites at each timestep. In the original geometry, two of the six neighboring sites around *GAL2* were already blocked by chromosomal volume. We therefore incrementally added one, two, three, or four additional blocked neighboring sites around the *GAL2* locus.

Blocking one or two additional neighboring sites produced no significant change in G2 abundance. Blocking three neighboring sites produced a modest reduction. When all four remaining neighboring sites were blocked, G2 abundance no longer increased, consistent with the loss of transcription and translation events at the inaccessible *GAL2* locus, and instead decreased due to molecular degradation. These results support the interpretation that local excluded-volume effects near gene loci can reduce effective promoter accessibility when the locus becomes sufficiently constrained.

However, the modest effect observed under partial blocking should be interpreted in the context of other model simplifications. As shown in Fig. S11, the total number of nuclear G80d repressors rapidly decreased to approximately five molecules during the simulation, likely due to fast asymmetric diffusion. Thus, the limited effect of chromosome geometry is unlikely to be explained solely by the absolute nuclear abundance of the repressor. Instead, it may reflect simplified treatment of

transcriptional regulation and nucleocytoplasmic transport in the present model. Specifically, the model does not explicitly represent chromatin remodeling, preinitiation-complex recruitment, or regulated transport through the nuclear pore complex. For example, G80d may require additional regulatory signals or transport mechanisms to pass through the nuclear pore complex from the nuclear side. These processes are beyond the scope of the present chromosome-geometry perturbation analysis but may be important for future spatial models of gene regulation.

4 Reduced Nucleoplasmic Diffusion and Gene Positioning

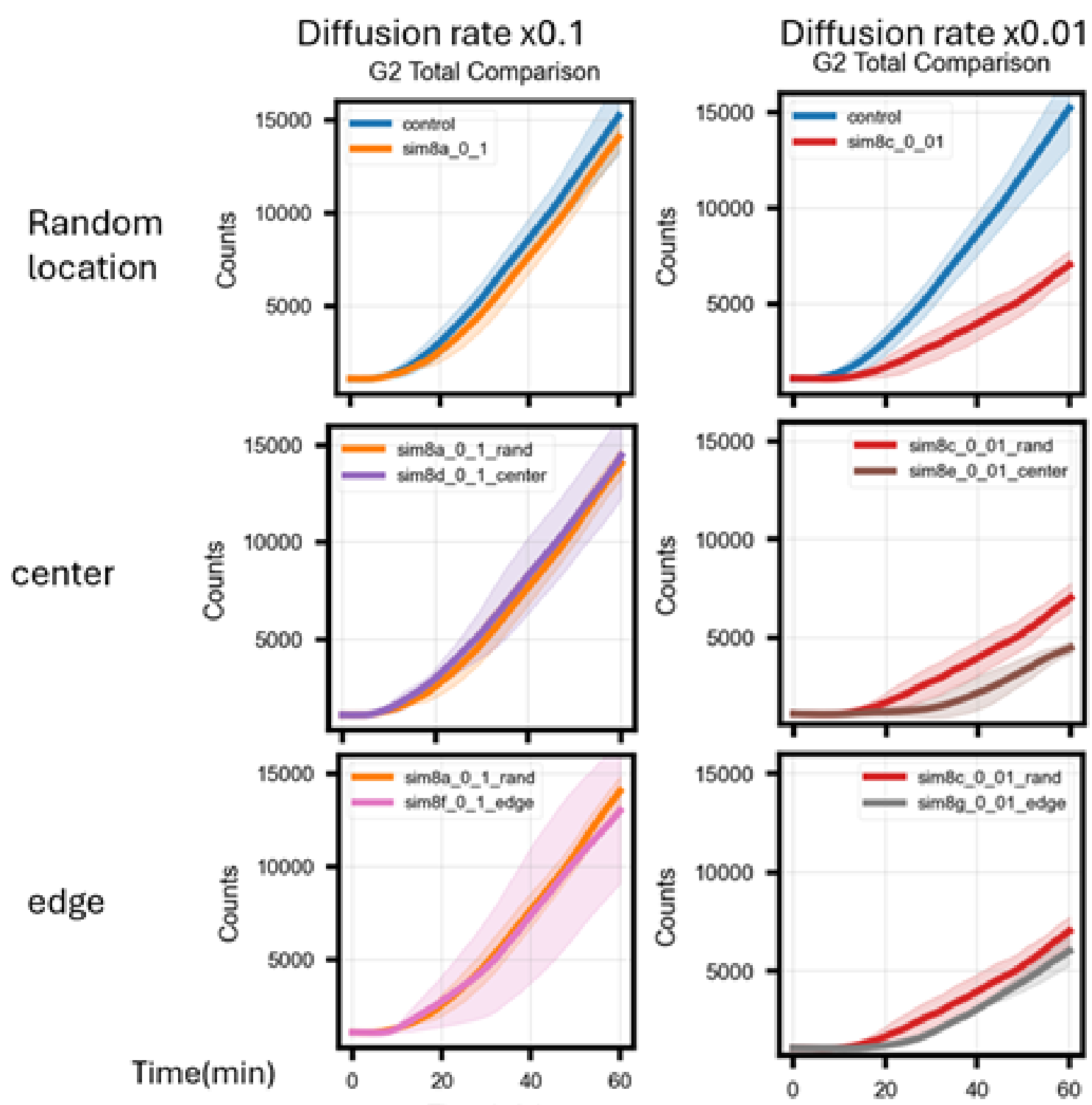

Fig P. Reduced nucleoplasmic diffusion reveals a stronger effect of gene positioning on Gal2p production. RDME–ODE simulations were performed with nucleoplasmic diffusion reduced to 0.1× or 0.01× of the baseline rate. At 0.1× diffusion, Gal2p transporter production remained similar across random, center, and edge gene-positioning conditions. At 0.01× diffusion, overall Gal2p production decreased, and genes positioned near the nuclear center produced less Gal2p than randomly positioned genes. Shaded regions indicate variation across three replicates.

To test whether gene positioning becomes more important under slower nucleoplasmic diffusion, we performed additional RDME–ODE simulations. We reduced the diffusion coefficients of all nucleoplasmic species to 0.1× and 0.01× of the baseline values, with three replicates for each condition.

At 0.1× diffusion, GAL2 expression was similar to the baseline case. At 0.01× diffusion, Gal2p transporter abundance decreased, likely because slow nuclear mRNA diffusion reduced efficient transcript transport.

We then repeated the 0.1× and 0.01× diffusion simulations with genes placed either near the nuclear center or near the nuclear edge. Under 0.1× diffusion, gene position had only a small effect. Under 0.01× diffusion, center-positioned genes produced less Gal2p than randomly positioned genes.

These results support our prediction that gene positioning becomes more influential when nucleoplasmic diffusion is strongly reduced.

5 Comparison of Predicted and Measured Final Fold Changes of GAL Proteins Across Experimental and Modeling Studies

Both results are close to what Ramsey et al. reported around x10 fold changes for protein Gal1p/Grep in 420 min simulation with Ramsey's original ODE model. [3].

Table Q. Fold-change comparison of GAL proteins under two galactose concentrations.

Species (Total)	5.55 mM/ 0.1% (Ramsey, ODE)	11.1 mM/ 0.2 %(Ramsey, ODE)
Gal1p/Grep	9.23	10.49
Gal2p	12.81	14.44
Gal3p	6.00	6.19
Gal80p	2.51	2.58

Table R. Comparison of Initial Protein Copy Numbers from Ramsey's [3] and Paulo's Datasets [7]. Values from Ramsey et al. were extracted from the Supplemental Information of the original publication. Values from Paulo et al. represent mean proteomics abundances measured in 2% raffinose medium. All quantities were rounded to the nearest integer to satisfy the requirement that molecular counts be integers in stochastic simulations.

Species (Total)	Ramsey	Paulo
Gal1p	132	7733
Gal2p	1157	2184
Gal3p	4341	1777
Gal80p	3	62
Gal4p	618	2033
Reporter protein	628	NA

6 Comparison of promoter-binding propensity in CME and RDME

In this section, we compare the propensity of the repression reactions of each galactose gene in the CME versus the RDME treatment of the system. We show that, even though the instantaneous propensity of the RDME treatment is orders of magnitude higher than that of the CME (due to a substantially decreased voxel size), the overall repression of the CME system is actually greater across time. Thus, there are a higher proportion of active genes in the RDME replicates than there are in the CME replicates, thereby contributing to the increased activation of the galactose genes in the RDME treatment (see Figure 5A).

Consider the reaction where repressor G_{80d} binds to activated gene G_{act} to form G_{rep}, the repressed gene:

$$G_{\text{act}} + G80d \longrightarrow G_{\text{rep}},$$

with macroscopic association constant k_{rep}. Because the reaction occurs in the nucleus, let V_{nuc} denote the accessible nuclear volume and V_{cell} denote the accessible cell volume in the CME scenario.

We define $A^X(t) \in \{0, 1\}$ as the indicator that the promoter is active in model $X \in \{\text{CME}, \text{RDME}\}$. For a finite trajectory, the conditional expectation of a quantity Y^X during promoter-active periods is estimated by the following equation:

$$\widehat{\mathbb{E}}\left[Y^X \mid A^X = 1\right] = \frac{\displaystyle\int_0^T A^X(t) Y^X(t)\, dt}{\displaystyle\int_0^T A^X(t)\, dt}.$$

In the CME model, let $S^{\text{CME}}_{\text{G80d,nuc}}(t)$ be the total nuclear G80d copy number. As such, the repression propensity is as follows:

$$P^{\text{rep}}_{\text{CME}}(t) = \frac{k_{\text{rep}}}{N_A V_{\text{cell}}} A^{\text{CME}}(t) S^{\text{CME}}_{\text{G80d,nuc}}(t).$$

In the RDME model, assume that the promoter is located in voxel j, with volume V_j, and let $S^{\text{RDME}}_{\text{G80d},j}(t)$ be the local G80d copy number. Under the standard local RDME mass-action rate,

$$P^{\text{rep}}_{\text{RDME}}(t) = \frac{k_{\text{rep}}}{N_A V_j} A^{\text{RDME}}(t) S^{\text{RDME}}_{\text{G80d},j}(t).$$

The exact active-state-conditioned propensity ratio is therefore

$$R_{\text{rep}} = \frac{\mathbb{E}\left[P^{\text{rep}}_{\text{RDME}} \mid A^{\text{RDME}} = 1\right]}{\mathbb{E}\left[P^{\text{rep}}_{\text{CME}} \mid A^{\text{CME}} = 1\right]} = \frac{V_{\text{cell}}}{V_j} \frac{\mathbb{E}\left[S^{\text{RDME}}_{\text{G80d},j} \mid A^{\text{RDME}} = 1\right]}{\mathbb{E}\left[S^{\text{CME}}_{\text{G80d,nuc}} \mid A^{\text{CME}} = 1\right]}.$$

To estimate the repression propensity without assuming a spatially uniform G80d distribution(non-stationary distribution), we directly use the G80d abundance in the promoter-containing RDME voxel. Conditional on the promoter being active, the mean RDME repression propensity is

$$\mathbb{E}\left[P^{\text{rep}}_{\text{RDME}} \mid A^{\text{RDME}} = 1\right] = \frac{k_{\text{rep}}}{N_A V_j} \mathbb{E}\left[S^{\text{RDME}}_{\text{G80d},j} \mid A^{\text{RDME}} = 1\right].$$

The corresponding CME repression propensity is

$$\mathbb{E}\left[P^{\text{rep}}_{\text{CME}} \mid A^{\text{CME}} = 1\right] = \frac{k_{\text{rep}}}{N_A V_{\text{cell}}} \mathbb{E}\left[S^{\text{CME}}_{\text{G80d,nuc}} \mid A^{\text{CME}} = 1\right].$$

Therefore, under the standard local RDME mass-action rate, the conditional RDME/CME repression-propensity ratio is

$$\boxed{R_{\text{rep}} = \frac{V_{\text{cell}}}{V_j} \frac{\mathbb{E}\left[S^{\text{RDME}}_{\text{G80d},j} \mid A^{\text{RDME}} = 1\right]}{\mathbb{E}\left[S^{\text{CME}}_{\text{G80d,nuc}} \mid A^{\text{CME}} = 1\right]}}.$$

For a cubic RDME voxel with side length 28 nm,

$$V_j = (28\,\text{nm})^3 = 2.1952 \times 10^{-5}\,\mu\text{m}^3.$$

If the CME repression propensity uses $V_{\text{cell}} = 33.3\,\mu\text{m}^3$, then

$$\frac{V_{\text{cell}}}{V_j} = \frac{33.3}{2.1952 \times 10^{-5}} \approx 1.517 \times 10^6.$$

Hence,

$$\boxed{R_{\text{rep}} \approx 1.517 \times 10^6 \frac{\mathbb{E}\left[S^{\text{RDME}}_{\text{G80d},j} \mid A^{\text{RDME}} = 1\right]}{\mathbb{E}\left[S^{\text{CME}}_{\text{G80d,nuc}} \mid A^{\text{CME}} = 1\right]}}.$$

This equation essentially tells us the ratio of RDME to CME propensities for repression of a gene, when the gene is in its active state and is a function of time. If the ratio is above 1, the RDME propensity for repression exceeds the CME propensity and when it is below 1, the CME propensity exceeds the RDME propensity. We hypothesized that the ratio in this equation would average to be less than 1 across the simulation time, thereby making the propensity for a CME gene repression reaction higher than that of an RDME repression reaction.

To test this, we used our current simulation data resolved at one-second intervals. For each trajectory (CME = 20 and RDME = 5), and at each one-second interval we computed the expectation value of either $S^{CME}_{G80d,nuc}(t)$ or $S^{RDME}_{G80d,j}(t)$. Expectation values in the CME regime could be computed using the time-dependent quantity of nuclear repressors during the times which the gene was in the active state. For the RDME regime, because we had only one-second resolved time series data, we were not able to track every instance of the repressor entering the gene voxel. Instead, we estimated this quantity by determining the number of times the repressed gene complex changed from 1 to 0 counts. In this instant, a repressor molecule would then have been present in the gene voxel and therefore able to react with the gene. Although this estimation does not account for other times the repressor would be transiently in the same subvoxel as the gene, we address why this issue does not affect our calculations in a following paragraph.

As a result of the computation of the expectation values of both the CME and RDME scenario trajectories, we obtained time-dependent traces of the expectation values for each replicate. In order to compute the ratio of expectation values for each timepoint, we averaged the expectation values at each time point across all trajectories for each regime independently, thereby creating a single time trace which represented the average expectation value for each regime. Next, we excluded time points from our CME trajectories with expectation value traces with less than four active genes (2,827 of 3,600 time points satisfy this criterion; for average expectation values across trajectories in CME and RMDE scenarios, see Figure S17-A). Doing so resulted in us considering three of the seven repressor-gene encounters due to the other four having a low number of CME trajectories with active genes (see Figure S17-B for the three events where $R^{local}_{rep}(t)$ was greater than 0).

After these calculations, we determined the volumetric ratio V_{cell}/V_j . For the 28-nm RDME voxel, $V_{cell}/V_j = \frac{33.3\mu m^3}{0.028\mu m^3} = 1.51710^6$.

We then plotted the replicate-averaged value of $R^{local}_{rep}(t)$ across all replicates for the entirety of the simulation time (see Figure S17-B). As an additional reference, Figure S17-C shows the number of promoter-active trajectories in both CME and RDME regimes across the entirety of the simulation time. The main result from these analyses is the near-zero baseline of Figure S17-B rather than the rare peaks. Of the 2,827 supported time points, 2,824 (99.9%) have $R^{local}_{rep}(t) = 0$. The CME denominator remains nonzero because its well-stirred nuclear G80d pool decreases from approximately 50 molecules to a plateau of about 3–5 molecules. In contrast, a G80d

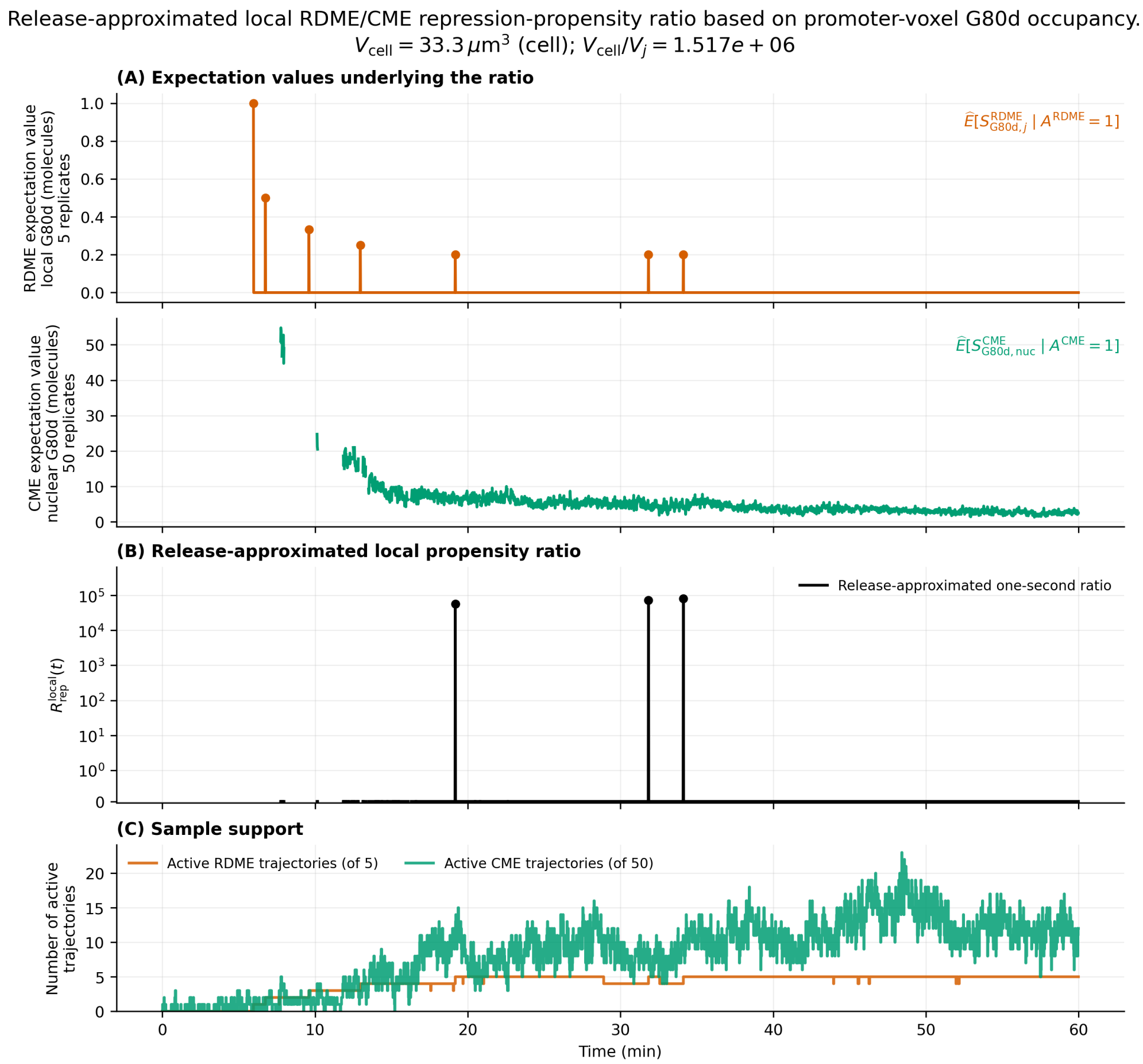

Fig Q. Release-approximated local RDME/CME repression-propensity ratio. Five RDME and fifty CME replicates; all quantities are conditioned on an active promoter, $\widehat{\mathbb{E}}[\cdot \mid A^X = 1]$. **(A)** Repressor occupancy in RDME (upper) and nuclear G80d abundance in CME (lower), in molecules. Each point is the ordinary mean over the promoter-active replicates, active replicates without a repressor counted as zero, so one occupied voxel among n active RDME replicates reads $1/n$. As the local occupancy is zero or one, the upper curve is also the local-encounter frequency and is not repeated in (C). **(B)** $R^{\text{local}}_{\text{rep}}(t)$, obtained by evaluating the boxed ratio above on the two curves of (A). Values are shown only where at least 1 of the 5 RDME and 4 of the 50 CME replicates are promoter-active; unsupported points are blank, not zero. The axis is symmetric-logarithmic and linear below unity so that exact zeros stay visible. **(C)** Numbers of promoter-active RDME and CME trajectories supporting each time point. The release approximation assigns one local G80d molecule to the activated snapshot of each saved $DG2\_G4d\_G80d \rightarrow DG2\_G4d$ transition, giving seven events, of which three had sufficient CME support.

molecule is present in the 28-nm promoter voxel in only 7 of 3,600 promoter-active RDME snapshots, corresponding to approximately 1% of the active time.

With these calculations in mind, we can compute the average $R_{rep}^{local}(t)$ across our entire simulation to be as follows:

$$\frac{5.710^4 + 7.410^4 + 8.210^4}{2824} = 75.42$$

With each of the numerator terms coming from the magnitude of the three release-event peaks in Figure S17-B. This result would seem to indicate that the RDME gene repression propensity is generally higher than the CME propensity.

Importantly, however, even though our one-second resolved data would suggest that the repressor remained in the same subvoxel as the activated gene for an entire second, these release-associated events cannot be treated as one-second residence events. This is because for a standard cubic RDME lattice with voxel width h=0.028 μm and diffusion coefficient $D = 1\mu m^2 s^{-1}$, the hopping rate to each neighboring voxel is D/h^2. For an interior voxel with six accessible neighbors, the total escape rate and mean first-exit time are $k_{escape} = 6D/h^2$ and $\tau_{res} = h^2/6D = 1.3110^{-4}s = 0.13ms$.

Thus, assigning each release-associated event one full second of occupancy overestimates its expected duration by approximately $1/(1.3110^{-4}) = 7.710^3$. The previously calculated ratio of time-averaged propensities, approximately 75, arose from this one-second-bin assignment and is therefore misleading. Rescaling that descriptive value by the diffusion-based residence time gives the following: $75 \times \frac{1.3110^{-4}s}{1s} = 9.810^{-3}$.

Therefore, the ratio of RDME to CME repression propensities substantially favors CME repression events, thereby contributing to the increased gene activation reported in Figure 5A. This value should be regarded as an order-of-magnitude estimate rather than an exact measurement because it assumes an interior voxel with six neighbors, diffusion-controlled first exit, and no contribution from subsequent return visits. Exact integration would require event-level diffusion times or substantially finer temporal output. Nevertheless, the corrected estimate is much smaller than unity and supports the robust qualitative conclusion: repression in the RDME is highly intermittent and encounter-limited. Rare occupancy of the promoter voxel generates very large instantaneous local propensities, but the encounters are sufficiently brief that the time-integrated RDME repression propensity is expected to remain well below the continuously nonzero well-stirred CME propensity.

7 Mathematical interpretation of ER-dependent translation and trafficking

To formalize the impact of endoplasmic reticulum (ER) geometry on transporter expression, we describe the process using a minimal diffusion–encounter and compartmental trafficking framework. Translation initiation of *GAL2* mRNA is treated as a two-step process consisting of (i) diffusion-limited encounter with ER-bound ribosomes and (ii) a local biochemical initiation step. The effective ER-dependent initiation rate is written as

$$\frac{1}{k_{\text{init}}^{\text{ER}}} = \frac{1}{k_{\text{diff}}^{\text{ER}}} + \frac{1}{k_{\text{chem}}}, \qquad k_{\text{diff}}^{\text{ER}} \sim 4\pi D_{\text{mRNA}} a \rho_{\text{ER-ribo}},$$

where D_{mRNA} is the effective diffusion coefficient of *GAL2* transcripts, a is an effective capture radius, and $\rho_{\text{ER-ribo}}$ is the surface density of ER-bound ribosomes.

Following synthesis, Gal2p dynamics are modeled using a two-compartment trafficking system:

$$\frac{dG_{\mathrm{ER}}}{dt} = J_{\mathrm{syn}}(t) - k_{\mathrm{export}} G_{\mathrm{ER}} - k_{\mathrm{deg,ER}} G_{\mathrm{ER}},$$
$$\frac{dG_{\mathrm{PM}}}{dt} = k_{\mathrm{export}} G_{\mathrm{ER}} - k_{\mathrm{int}} G_{\mathrm{PM}} - k_{\mathrm{deg,PM}} G_{\mathrm{PM}},$$

where G_{ER} and G_{PM} denote Gal2p abundance in the ER and plasma membrane compartments, respectively.

ER geometry enters the model through an effective trafficking time,

$$\tau_{\mathrm{ER}}^{\mathrm{geom}} \sim \frac{\left(L_{\mathrm{ER}}^{\mathrm{eff}}\right)^2}{2D_{\mathrm{ER}}} + \frac{L_{\mathrm{ER}}^{\mathrm{eff}}}{v}, \qquad k_{\mathrm{export}} \approx \frac{1}{\tau_{\mathrm{ER}}^{\mathrm{geom}}},$$

where $L_{\mathrm{ER}}^{\mathrm{eff}}$ is an effective path length within the ER network, D_{ER} is the lateral diffusion coefficient of Gal2p, and v represents any directed transport component. Increased ER geometric complexity therefore decreases k_{export}, lowering steady-state plasma-membrane abundance

$$G_{\mathrm{PM}}^{*} \approx \frac{k_{\mathrm{export}}}{k_{\mathrm{int}} + k_{\mathrm{deg,PM}}} \cdot \frac{J_{\mathrm{syn}}}{k_{\mathrm{export}} + k_{\mathrm{deg,ER}}}.$$

This reduction in plasma-membrane Gal2p directly limits galactose uptake, thereby reducing intracellular galactose levels.

8 Sensitivity Analysis on kinetic parameters

To further support our argument that the increased probability of the GAL2 gene being in the activated state leads to higher levels of G2 protein translation within the RDME–ODE framework, we conducted a parameter sensitivity analysis. Specifically, we first constructed a pure ODE model based on the CME–ODE formulation and systematically varied all kinetic parameters within a range of $0.1\times$ to $10\times$ their baseline values. Parameters that produced more than a twofold change in G2 abundance after 60 minutes of simulation were identified(Fig R(B)). We then generated pairwise heatmaps of these parameters to visualize their combined effects. Notably, the diffusion rates of G80d between the cytoplasm and nucleoplasm were capable of driving more than a twofold change in G2 abundance together(Fig R(A, C)). Moreover, when two such parameters were altered simultaneously, the resulting G2 abundance closely approximated that predicted by the RDME–ODE model at 60 minutes(Fig R(D)). These findings support our conclusion that the observed increase arises primarily from asymmetric diffusion.

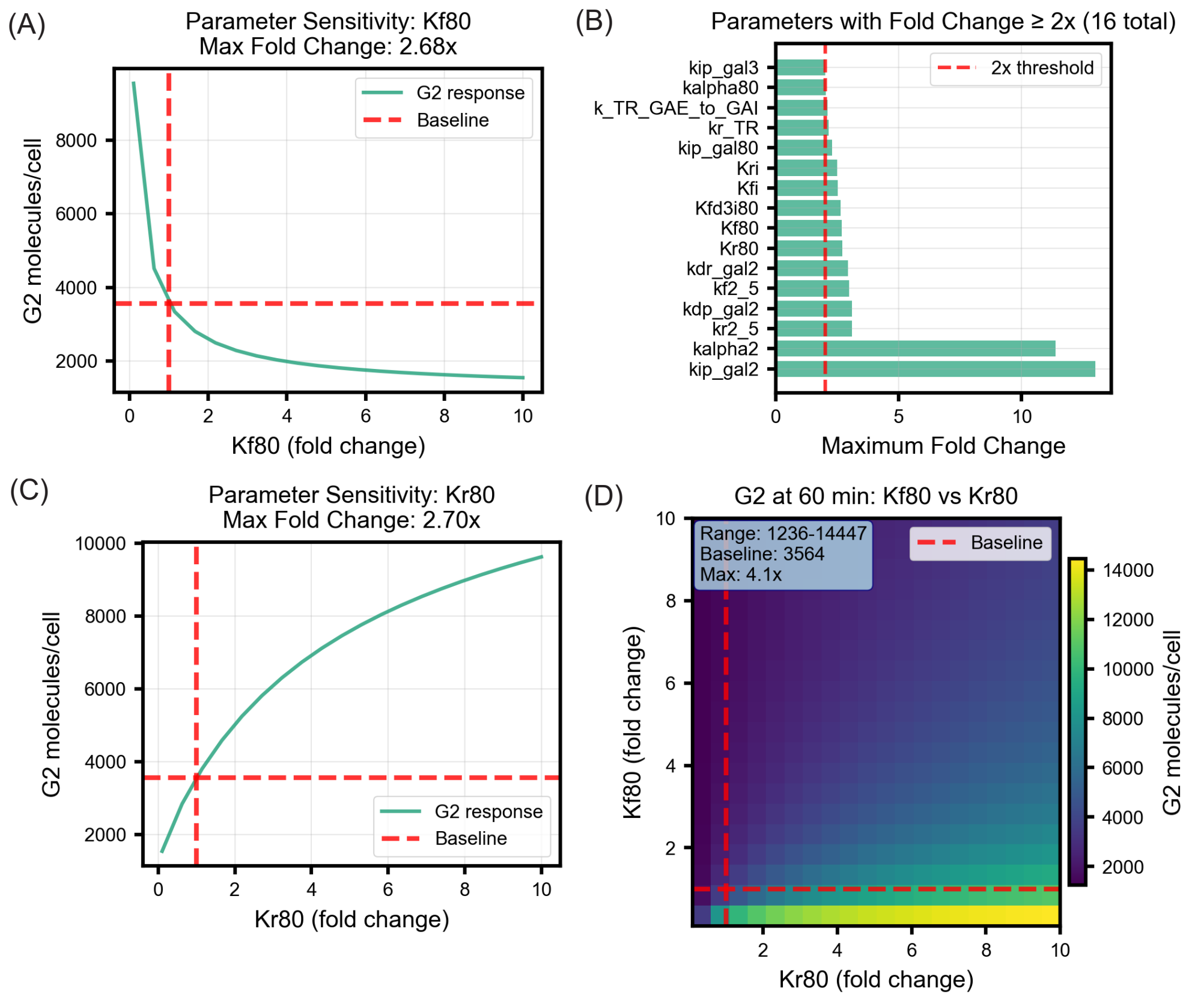

Fig R. Parameter Sensitivity Analysis Results. (A) Effect of single-parameter perturbation of the G80d transition rate $kf80$ from the cytoplasm to the nucleoplasm. (B) Kinetic parameters exhibiting more than a twofold change in G2 abundance within the 0.1× to 10× perturbation range. (C) Effect of single-parameter perturbation of the G80d diffusion rate $Kr80$ from the nucleoplasm to the cytoplasm. (D) Combined perturbation of both G80d diffusion rates (cytoplasm → nucleoplasm and nucleoplasm → cytoplasm).

References

1. Bianchi DM, Peterson JR, Earnest TM, Hallock MJ, Luthey-Schulten Z. Hybrid CME–ODE method for efficient simulation of the galactose switch in yeast. IET Systems Biology. 2018;12(4):170–176. doi:10.1049/iet-syb.2017.0070.

2. Earnest TM, Watanabe R, Stone JE, Mahamid J, Baumeister W, Villa E, et al. Challenges of Integrating Stochastic Dynamics and Cryo-Electron Tomograms in Whole-Cell Simulations. The Journal of Physical Chemistry B. 2017;121(15):3871–3881. doi:10.1021/acs.jpcb.7b00672.

3. Ramsey SA, Smith JJ, Orrell D, Marelli M, Petersen TW, De Atauri P, et al. Dual feedback loops in the GAL regulon suppress cellular heterogeneity in yeast. Nature Genetics. 2006;38(9):1082–1087. doi:10.1038/ng1869.

4. Thornburg ZR, Bianchi DM, Brier TA, Gilbert BR, Earnest TM, Melo MCR, et al. Fundamental behaviors emerge from simulations of a living minimal cell. Cell. 2022;185(2):345–360.e28. doi:10.1016/j.cell.2021.12.025.

5. Duan Z, Andronescu M, Schutz K, McIlwain S, Kim YJ, Lee C, et al. A three-dimensional model of the yeast genome. Nature. 2010;465(7296):363–367. doi:10.1038/nature08973.

6. Cherry JM, Hong EL, Amundsen C, Balakrishnan R, Binkley G, Chan ET, et al. Saccharomyces Genome Database: the genomics resource of budding yeast. Nucleic Acids Research. 2012;40(D1):D700–D705. doi:10.1093/nar/gkr1029.

7. Paulo JA, O'Connell JD, Gaun A, Gygi SP. Proteome-wide quantitative multiplexed profiling of protein expression: carbon-source dependency in Saccharomyces cerevisiae. Molecular Biology of the Cell. 2015;26(22):4063–4074. doi:10.1091/mbc.E15-07-0499.
