# Supplementary material for: Spatially resolved reaction–diffusion modeling reveals effects of intracellular spatial heterogeneity on yeast galactose network dynamics
Source: PLoS Comput Biol. 2026 Oct 5;22(10):e1014811. doi: 10.1371/journal.pcbi.1014811 (PMC13705588; doi:10.1371/journal.pcbi.1014811)
Supplement: Supplementary file 2 [file pcbi.1014811.s009.docx]

# Response to Editor and Reviewer Comments:

This document includes our responses to all Editor and Reviewer comments on the submission of “Spatially resolved reaction diffusion modeling reveals effects of intracellular spatial heterogeneity on yeast galactose network dynamics” (PCOMPBIOL-D-26-00367) to the journal of PLOS Computational Biology. All Editor and Reviewer comments have been numbered and are represented with brown text. All of our responses are listed below the respective comments in black text.

# Journal Requirements/Editor Comments:

1. Please ensure that the CRediT author contributions listed for every co-author are completed accurately and in full. At this stage, the following Authors/Authors require contributions: Abner T. Apsley, Emmy Earnest, Julia Mahamid, Marie-Christin Spindler, Zane Thornburg, Tianyu Wu, and Zaida Luthey-Schulten. Please ensure that the full contributions of each author are acknowledged in the "Add/Edit/Remove Authors" section of our submission form.
    The list of CRediT author contributions may be found here: [https://journals.plos.org/ploscompbiol/s/authorship#loc-author-contributions](https://urldefense.com/v3/__https:/journals.plos.org/ploscompbiol/s/authorship*loc-author-contributions__;Iw!!DZ3fjg!8gj4I5gVF9biZJcMF_HWcvZQFdQjztiVJxLz66roC8_WmaWKFzlT6RNPlg3FtvL313zC1WZYS6yYydBhKf36Qw$)

The following is a list of each author’s contributions:

Abner T. Apsley: Writing – Original Draft Preparation, Writing – Review & Editing, Visualization, Investigation, Supervision
Emmy Earnest: Software, Methodology
Julia Mahamid: Writing – Review & Editing, Funding Acquisition, Conceptualization,Resources,Supervision
Marie-Christin Spindler: Writing – Original Draft Preparation, Investigation, Formal Analysis
Zane Thornburg: Software, Supervision
Tianyu Wu: Writing – Original Draft Preparation, Data Curation, Formal Analysis, Investigation, Visualization
Zaida Luthey-Schulten: Writing – Review & Editing, Funding Acquisition, Conceptualization,Resources,Supervision, Project Administration

We have also updated each author’s contributions on the “Add/Edit/Remove Authors” section of the submission form.

2. We ask that a manuscript source file is provided at Revision. Please upload your manuscript file as a .doc, .docx, .rtf or .tex. If you are providing a .tex file, please upload it under the item type u2018LaTeX Source Fileu2019 and leave your .pdf version as the item type u2018Manuscriptu2019.

   We have provided a .tex file.
3. Please upload all main figures as separate Figure files in .tif or .eps format. For more information about how to convert and format your figure files please see our guidelines:

   All figures were regenerated based on the TIFF requirements listed below:

| [**File Format**](https://journals.plos.org/ploscompbiol/s/figures#loc-file-format) | TIFF or EPS |
| --- | --- |
| [**Dimensions**](https://journals.plos.org/ploscompbiol/s/figures#loc-dimensions) | Width: 789 – 2250 pixels (at 300 dpi). Height maximum: 2625 pixels (at 300 dpi). |
| [**Resolution**](https://journals.plos.org/ploscompbiol/s/figures#loc-resolution) | 300 – 600 dpi |
| [**File Size**](https://journals.plos.org/ploscompbiol/s/figures#loc-file-size) | <10 MB |
| [**Text within Figures**](https://journals.plos.org/ploscompbiol/s/figures#loc-text-within-figures) | Arial, Times, or Symbol font only in 8-12 point |
| [**Figure Files**](https://journals.plos.org/ploscompbiol/s/figures#loc-figure-files) | Fig1.tif, Fig2.eps, and so on. Match file name to caption label and citation. |
| [**Captions**](https://journals.plos.org/ploscompbiol/s/figures#loc-captions) | In the manuscript, not in the figure file. |

4. you have uploaded Supporting Information files, but you have not included a list of legends. Please add a full list of legends for your Supporting Information files after the references list.

   A full list of legends for all Supporting Information files has been added to the revised manuscript. The legends are shown below:

5. Data Availability Statement is currently missing the DOI/accession number of each dataset OR a direct link to access each dataset. If your manuscript is accepted for publication, you will be asked to provide these details on a very short timeline. We therefore suggest that you provide this information now, though we will not hold up the
   peer review process if you are unable.

   We have provided a Code and Data Availability statement in the revised manuscript which reads as follows:

6. Amend your detailed **Financial Disclosure statement**. This is published with the article. It must therefore be completed in full sentences and contain the exact wording you wish to be published.
   State the initials, alongside each funding source, of each author to receive each grant. For example: "This work was supported by the National Institutes of Health (####### to AM; ###### to CJ) and the National Science Foundation (###### to AM)."

   State what role the funders took in the study. If the funders had no role in your study, please state: "The funders had no role in study design, data collection and analysis, decision to publish, or preparation of the manuscript."

    If any authors received a salary from any of your funders, please state which authors and which funders.
   Please ensure that the funders and grant numbers match between the Financial Disclosure field and the Funding Information tab in your submission form. Note that the funders must be provided in the same order in both places as well.

Our Financial Disclosure Statement now reads as follows:

This work was supported by the European Research Council (Starting Grant 3DCellPhase 760067 to J.M.), the Chan Zuckerberg Initiative (grant 2023-331612 to J.M.), EMBL (to J.M.), the National Science Foundation Science and Technology Center for Quantitative Cell Biology (DBI-2243257 to T.W., M.C.S., A.T.A and Z.L.S.), and the Cancer Center at Illinois–Beckman Institute Postdoctoral Fellows Program, sponsored by the Cancer Center at Illinois and the Beckman Institute for Advanced Science and Technology, University of Illinois Urbana–Champaign (to Z.R.T.). This work also used Delta and DeltaAI at the National Center for Supercomputing Applications through allocation BIO250002 from the Advanced Cyberinfrastructure Coordination Ecosystem: Services & Support program, which is supported by the U.S. National Science Foundation under awards 2138259, 2138286, 2138307, 2137603, and 2138296. The funders had no role in study design, data collection and analysis, decision to publish, or preparation of the manuscript. The content is solely the responsibility of the authors and does not necessarily represent the official views of the program sponsors.

7. Kindly amend your competing statement in the online submission form to align with the journal's style guidelines: '**The authors declare that there are no competing interests.**'

This has to be changed in the submission website.

# We thank all the reviewers for their helpful comments, and have used our response to modify the manuscript in red as requested by the journal

# Reviewer 1:

## Major Problems:

1. In Result 3.1, it is shown that there is a difference between the well-stirred model and the model which incorporates spatial aspects, with the conclusion that such **incorporation can lead to shifts in the timing**. I would have loved to see more depth on what specifically is causing the models to have different results. With regard to the latter, an explanation is given but it would be great to **use model perturbation to illustrate this more clearly.**

   We thank the reviewer for this comment. To address this point, we performed five additional simulation cases in which one parameter was perturbed at a time to identify factors contributing to the difference between the CME–ODE and RDME–ODE simulations in Fig. 5C. All additional simulations were performed in the CME–ODE framework, with 10 replicates for each case.

   In **Sim1**, we changed the initial distribution of G80d from a uniform distribution to a distribution proportional to the nuclear and cytoplasmic volume ratio used in the RDME–ODE simulation. This change produced no significant difference in G2 abundance over the 60-min simulation, suggesting that the initial G80d partitioning is not the major source of the discrepancy.

   In **Sim2**, we reduced the promoter-binding kinetic parameter to account for the approximately 16s search time required for the promoter to leave and re-enter the promoter region in the nucleus in the RDME–ODE simulation. This perturbation produced a substantial change in G2 abundance, as shown in the Sim2 result.The effects of diffusion need to be included in the future CME kinetic parameters to use the simpler and faster description.

   In **Sim3**, we tested whether local volume effects could explain the discrepancy. To mimic a 5-fold reduction in effective local volume, we scaled both the binding and unbinding rates by 5-fold while keeping the equilibrium constant unchanged. Thus, binding and unbinding occurred five times faster, while the equilibrium affinity was preserved. Under this condition, G2 abundance increased.

   In **Sim4**, we examined the effect of asymmetric diffusion through nuclear pores. Based on our calculated ratio, we either increased cytoplasm-to-nucleus diffusion by 7-fold (**Sim4a**) or decreased nucleus-to-cytoplasm diffusion by 7-fold (**Sim4b**). Both perturbations increased G2 abundance, indicating that asymmetric nuclear transport may also contribute to the CME–ODE/RDME–ODE differences observed.

   Finally, in **Sim5**, we combined these contributing factors. The combined perturbation did not produce a simple additive increase in G2 abundance. Instead, the response appeared to approach an upper bound, suggesting that these mechanisms interact nonlinearly rather than stacking independently.

   To supplement our coverage of these topics in the Discussion (Section 4.1), we have included the following figure in our Supplemental File 1 and reference corresponding figure results in the respective subsections of the Discussion. We realize that the biology community does not always have access to state of the art GPUs, but improving on the kinetic model when only CPUs and CME/ODE methods are available involves re-parameterization to account for the effects of diffusion and is only possible once the RMDE simulations have been performed on galactose concentrations less than 40mM.

2. Result 3.2 focuses on adding a detailed pre-determined physical chromosome to the simulations. I would have thought that this would lead to a substantial difference in the simulation outcomes, due to the specific localization of the genes involved as well as the diffusion obstacles they mention. However, they don’t observe a difference, **which is attributed to the concentrations of activators and repressors in the nucleus**. It therefore seems straightforward to perform simulations in which these concentrations are increased, to try and find concentrations in which adding the phy**sical aspects of the chromosome would matter.**

   We thank the reviewer for this comment. We would like to clarify that this analysis is not based on concentration changes, but rather more on excluded-volume effects. Specifically, chromosomes are treated as physical obstacles that can prevent activators and repressors from accessing certain regions of the nucleoplasm, thereby reducing their probability of contacting gene loci.

To further display the effect of physical obstacles on accessibility of gene loci, we artificially introduced additional blocked lattice sites around the GAL2 gene to mimic increased local chromatin crowding. For each timestep in Lattice Microbes, diffusion events are allowed to take place across the six directly neighboring lattice sites. In the original geometry, two of the six neighboring sites around GAL2 were already blocked by chromosomal volume. We therefore incrementally added one, two, three, and four additional blocked sites around the GAL2 gene.

When one or two additional sites were blocked, GAL2 abundance showed no significant change. Blocking three neighboring sites produced a modest reduction. When all four remaining neighboring sites were blocked, GAL2 no longer increased (no transcription or translation events) and instead began to decrease due to degradation, as expected. This result supports the interpretation that local excluded-volume effects near gene loci can reduce effective promoter accessibility.

(control is the same as “with chromosome” trajectory in figure 6C)

Meanwhile, as shown in Figure S11, the total number of nuclear G80d repressors rapidly decreased to approximately five during the simulation, likely due to fast asymmetric diffusion. Therefore, we do not believe that the limited effect of chromosome geometry is primarily explained by the absolute nuclear concentration of the repressor. Rather, it is more likely attributable to the simplified treatment of transcription, meaning we ignored the chromatin remodeling and recruitment of the preinitiation complex, and also we simplified the nucleocytoplasmic transport by pure passive diffusion in our model. For example, G80d may require additional regulatory signals or transport mechanisms to pass through the nuclear pore complex from the nuclear side, which is beyond the scope of our chromosome-geometry perturbation study.

3. Result 3.5.1, we now see which model is now closest to experimentally-measured behaviors, but we definitely need some more detail here. For example, the impression one gets from Result 3.1 is that the comparisons between CME-ODE and RDME-ODE results and experiments should be similar. Then of course, there is the finding that even the Ramsey ODE model outperforms everything when it comes to Gal80p and that the most integrated model performs among the worst with regard to Gal3p. These need significantly more discussion. What could be done to get the most integrated model to match more correctly? Some treatment of this topic is given in Result 3.5.2, with mention of adding a inhibitory link between Gal1p and Gal80p. It seems like this would be an addition to the model that would add value to this specific study, and I can’t envision a future paper in which only this is added, so I would recommend performing those simulations and giving the results and analysis here.

We should have mentioned that we only had a so-called “Gold Standard” for fold changs (FC) at high galactose concentrations from the work of Paulo in Table 3. For low sugar concentrations we took the work of Ramsey who only provided experiments Gal1 and gfp. The values in Table 2 are from his ODE program. Finaly It is worth noting that the at the high galactose concentration (2% vs .2 % in Result 3.5.2) Table 3, the fold change(FC) of gal80p in Ramsey’s model(2.6) is roughly 5 fold smaller than Paulo’s experiment (12), whereas ours is only 2 fold larger (30.3).. Be that as it may, As we stated above in the response to the first comment, an important difficulty is finding the kinetic parameters for the CME-ODE treatment that account for spatial effects like diffusion and location of the process within the cell. Often we can only parameterize the CME-ODE treatment after the more time consuming RDME-ODE simulations or additional experimental information becomes available. To address this comment, we have performed additional simulations. The first set of simulations were spatially-homogeneous and included the aforementioned binding between the Gal1p-galactose complex and the Gal80p protein. According to a previous study by Tali Lavy, Hayato Yanagida and Dan S. Tawfik in 2016 (10.1093/molbev/msv240), at saturating galactose concentrations, the Gal1p-galactose complex associates with Gal80 around 0.246 times as well as the Gal3-galactose complex, and dissociates 3.036 times faster. Using these proportions, Gal1p binding and unbinding reactions with Gal80p were parameterized and added to spatially homogeneous versions of the current model. This model was then run for 20 replicates, at 60 minutes, and at an external galactose concentration of 11.1mM. Results from this set of simulations revealed that the Gal80p fold-change in this newly parameterized model was not significantly different from the value in the original CME-ODE model (new model fold-change: 1.98 +/- 0.38, p>0.05 when compared with previous model). These results suggest that the addition of the Gal1p inhibitory pathway would not affect the Gal80p fold-change in our current simulations.

We also performed a parameter screen of variables directly related to the fold change of G80, including the initial G80d abundance (G80d_init), GAL80 transcription rate (kalpha80), G80 mRNA degradation rate (kdr_gal80), and G80 translation rate (kip_gal80).

The screening results show that, with appropriate adjustment of G80-related parameters, the G80 fold change can be brought closer to Paulo's experimental data while causing only limited changes in the fold changes of several other GAL proteins. However, we do not interpret this as sufficient justification to adopt these parameters as the new default. These parameter changes are not independently constrained by experimental measurements and may compensate for model simplifications rather than reflect biologically meaningful regulation. In addition, some parameter combinations substantially perturb the fold changes of other GAL components, as highlighted by the two red boxes, indicating that improving the G80 fold change alone can introduce unintended network-level effects. Therefore, we use this screen primarily as a sensitivity analysis: it shows that the G80 discrepancy is parameter-dependent and potentially reducible, but it also illustrates that parameter tuning must be constrained by system-wide behavior rather than optimizing a single species in isolation.

We chose not to use those fitted parameters because they are fitted based on high concentration(111mM), and also we don’t know the mechanism behind those adjustments. Since our original kinetic parameters are originally from Ramsey’s 2006 paper which used 11.1mM, we did not want to change our model to use this set of fitted parameters for both high and low concentrations.

4. Another perturbation that could be simulated is suggested by a conclusion in 4.2.1: “We therefore expect gene positioning to become more influential in regimes with **slower nucleoplasmic diffusion o**r stronger crowding.” Again, I recommend testing this hypothesis in simulation.

   We greatly thank the reviewer for this comment. To address the concerns, we performed three additional RDME-ODE simulations, each with three replicates. We decreased the diffusion rate of everything in nucleoplasm by x0.1 and x0.01. We can see the abundance of transporters dropped when they decreased to x0.01, which is due to the slow diffusion of mRNA in the nucleus.

   Then, we did the same x0.1 and x0.01 with all genes in the center or the edge. We found out that when in the center case with diffusion rate x0.01, the overall production of gal2p transporter is smaller than in the random case( where genes are placed randomly in the nucleus). It proved that when genes are placed in the center, when diffusion rate is decreased to x0.01 of our current rate, it showed a discrepancy by reducing the production rate of galactose transporter gal2p.

We have added the figures to the appendix 16.

5. Finally, overall it feels like the manuscript should be more focused on the biological relevance and findings that were or could be generated from this work. The conclusions and discussion are more focused on the modeling side. **What are the takeaways for a yeast biologist?** Should they be using this model, even though some of the comparisons to experiment are not as close as others? If so, what are the main justifications for doing so?

    The main aim of the current manuscript is to provide information on how spatial heterogeneities in the form of either reaction locality and/or complex organelle geometries affect the overall results of cell-scale biological simulations. Specifically, we hoped to make the argument that cell-scale models of eukaryotic cells can be drastically affected by the inclusion of spatial heterogeneities and that additional efforts need to be taken to provide models under the well-stirred assumption.

Here we have modeled a small portion of the molecular processes in yeast, and the processes that we did model are not modeled in their full complexity (e.g., we did not explicitly include the action of RNA polymerase in the transcription of GAL genes, we have to estimate the mRNA counts for other membrane proteins transporting other nutrients besides galactose). A useful model for yeast biologists should be able to simulate a vast number of scenarios and environments to help make predictions about experiments that can be performed. With our current on-going model, it can already capture the fold increases of the well-studied genetic switch and is being used to analyze the effects of cytoplasmic obstacles like filaments on influencing the movements of tracer particles with various sizes. Simulations and experiments will appear in the follow-up works.

To make explicit the intentions of our current model, we have included the following sentence at the beginning of 4.3 Limitations and Future Directions

“While our current model can adequately predict galactose protein dynamics under a limited set of environmental conditions, it still requires further improvement. Many additional biological complexities and computational adjustments will need to be incorporated before the model can perform generalized cellular predictions accurately and computationally efficiently. ”

### **Minor Problem:**

6. Lines 518-523: confusing, I would rewrite and probably use multiple sentences

We have changed the sentence from:

“The differences between the well-stirred CME-ODE models and spatially-resolved RDME-ODE modes arise because when repression involves low-copy repressor acting on single genomic loci (GAL2 ), enforcing spatial locality reduces effective repression events and biases the system toward earlier onset of activation, which then propagates forward to Gal2p accumulation and intracellular galactose uptake (A more detailed explanation is provided in Discussion 4.2)”

To the following:

“Differences between well-stirred and spatially-resolved models can arise when a low-copy repressor molecule acts on a single genomic loci (e.i., *GAL2*). This is because enforcing spatial locality reduces effective repression events and biases the system toward earlier gene activation (see Discussion 4.2 for a more detailed explanation).”

7. Line 576: should be “Next, the converted”

Thank you, we have fixed this sentence to read as follows:

“Next, \ribosome densities were converted into…”

8. Legend of Fig. 9: two periods at the end

Thank you, we have deleted one of the periods.

# Reviewer 2:

## Major Problems:

9. Figure 4: The scaling behavior appears rather suboptimal, e.g. from 2 to 4 H100 GPUs, the runtime is only reduced by ~25%. Given the fact that one has to perform multiple runs anyway, I am wondering whether a parallelization-free code would not have been effectively more computationally efficient. This should be discussed.

We thank the reviewer for this comment. The suboptimal scaling arises primarily from communication overhead between GPUs in our domain-decomposed lattice. The simulation space is evenly partitioned, and GPUs must frequently exchange information to handle particle diffusion across subdomain boundaries. As the number of GPUs increases, this inter-GPU communication becomes a larger fraction of the total runtime, limiting scaling efficiency.

We agree that for smaller systems, using fewer GPUs (or even a single GPU) is more efficient. However, for large systems such as the full yeast model (192×192×192 voxels), a single-GPU run would require more than a day (>24 hrs), making multi-GPU execution necessary despite the communication overhead. We have added a discussion of this trade-off in the revised manuscript.

“The observed scaling is sublinear because the domain-decomposed RDME lattice requires frequent inter-GPU communication to resolve particle diffusion across subdomain boundaries. As the number of GPUs increases, this boundary-exchange overhead becomes a larger fraction of the total runtime, limiting parallel efficiency. Therefore, for smaller systems, fewer GPUs or a single GPU may be more efficient. However, for the full yeast model used here (192x192x 192 voxels), a single-GPU simulation required approximately 25 hours, making multi-GPU execution practically necessary despite the communication overhead. This trade-off explains why the multi-GPU solver improves practical wall-clock feasibility for large yeast-scale simulations, even though the scaling is not linear.”

10. L. 502 ff: I assume that these results depend massively on the model parameterization. In this case, it would be critical to know how the CME-ODE was parameterized in comparison to the RDME-ODE. As collision rates are volume dependent, the conversion of the parameter does not appear trivial. The geometry effect could simply be due to a suboptimal parameter “conversion”. Overall, the comparison between spatially resolved and homogeneous models remains largely qualitative.

We thank the reviewer for this important comment. In this study, the RDME–ODE model uses the same macroscopic kinetic parameter set as the previously published CME–ODE galactose-switch model, which was based on the Ramsey model. Our objective was not to perform a separate re-parameterization of the spatial model, but to isolate the consequences of introducing spatial locality and realistic intracellular geometry while holding the underlying biochemical parameterization fixed. We readily acknowledge from our work on the 4D simulations of a minimal cell (Cell 2026, see DnaA filament formation) effects of diffusion will give substantial differences in some of the parameters in moving from RDME to CME. This was also discussed briefly in reply to Reviewer 1.

Accordingly, the observed differences between the CME–ODE and RDME–ODE models are not an artifact of an arbitrary parameter conversion, but instead arise from the mechanistic differences inherent to the two formalisms. As we discuss in Sections 4.1.1–4.1.5, the spatial model explicitly imposes compartment-specific initial abundances, finite molecular search times, voxel-local bimolecular propensities, and geometry-dependent nuclear transport. These are precisely the physical effects that are absent from the well-mixed model and that the comparison is intended to reveal.

For this reason, the CME–ODE versus RDME–ODE comparison is intentionally framed as a controlled qualitative comparison under a shared baseline parameterization, rather than as an attempt to force exact quantitative agreement between well-mixed and spatial descriptions. We have already clarified the mechanistic basis of these differences in Sections 4.1.1–4.1.5, and have revised the text near line 502 to make this point more explicit.

Line 502: “The CME--ODE and RDME--ODE models use the same baseline macroscopic kinetic parameter set; thus, the differences observed here reflect the explicit introduction of spatial locality, compartmentalization, and geometry-dependent transport in the RDME framework rather than an independent re-parameterization of the biochemical network.”

11. Given the complexity of the model, a sensitivity analysis would have been valuable.

We thank the reviewer for this comment. We have already included one parameter-sensitivity analysis examining how the G80 transition rate contributes to the fold change of Gal2p transporter abundance (see response to Reviewer 1 - comment #3).

Because the model contains approximately 50 parameters, a full global sensitivity analysis is beyond the scope of the present study. Moreover, most parameters were adopted from previous studies rather than newly fitted in this work[18,35]. Therefore, our goal here is not to re-estimate the full parameter set, but to assess whether key parameter choices could influence the observed fold-change deviations.

To provide additional context, we performed a sensitivity analysis testing how the initial abundance of each protein affects the final protein abundance after 60 min of simulation. These results should be interpreted cautiously, given the uncertainty in the parameterization inherited from Ramsey’s model and the deviations in fold changes discussed in our manuscript. Briefly, we perturbed the initial abundance of each protein and measured the resulting final protein fold changes. The results are shown below. For visualization, the color bar represents log⁡2(FC). [All those parameter screening is done in ODE model]

In these heatmaps, the x-axis denotes the perturbed protein, whose initial abundance was altered, and the y-axis denotes the measured protein, whose final abundance at 60 min was quantified. Color intensity represents the log₂ fold change in final abundance relative to the baseline simulation. The left panel shows the response to a 10-fold reduction in initial protein abundance, whereas the right panel shows the response to a 10-fold increase.

Several patterns emerge from this perturbation analysis. First, the strongest effects occur along the diagonal, indicating that perturbing a protein’s initial abundance most directly affects its own final abundance. This self-effect is particularly pronounced for regulators Gal3, Gal80, and Gal4, suggesting that these components retain strong abundance memory over the 60-min simulation window or are subject to regulatory feedback that amplifies their initial perturbation. Second, the system displays an asymmetric response to decrease vs. increase of initial abundance. For the left plot, decreasing initial abundance of gal2p or gal3p will lead to less binding of repressor G80d and increase the final abundance of free repressor G80d. Third, repressor G80d appears as a key regulatory node. Decreasing the initial abundance of G80d leads to an overall more expression of other GAL genes(left plot). For the right plot, as expected, increased abundance of G80d will lead to less final abundance of other GAL genes. These observations are consistent with our simple kinetic model. This effect is less noticeable with activator G4d.

12. Section 4: In parts, this is not really a discussion but presents new results or at least discusses new aspects, e.g. Section 4.1.2 - 4.1.5. I would recommend that the authors consider a redistribution of the content.

We thank the reviewer for this comment. Although Sections 4.1.2 - 4.1.5 introduce additional concepts and some small additional “results”, we do not believe that this content should be redistributed. Our main findings from Section 3.1 are that the changes from homogeneous to spatially-resolved simulations cause changes in gene activation timing and subsequent protein and metabolite trajectories. In Sections 4.1.2-4.1.5, we aim to explain why these differences were observed. Additionally, according to the comments made by Reviewer #1, we have extended our work explaining why these two model types exhibit different behaviors, and these results have been added to the Supplementary Information.

### **Minor Problems:**

13. Some parts of the methods section read like an Introduction rather than a Materials & Methods section, e.g. Section 2.1.2. In my opinion, a Methods section should describe what has been used, not what could be used / is available. I would recommend integrating the respective section with the actual description of the work.

We thank the reviewer for their insight. While we would have included more of this information in the manuscript Introduction, we did not feel it added to the overall argument of our paper. Rather, we believe that it was important information for readers interested in knowing the details of how we simulated spatial heterogeneity. Additionally, the Introduction section already covered a substantial amount of topics and information, therefore, we did not want to introduce specific details about the simulation algorithms we use because we felt it was not central to our overall aims of the paper. As such, we decided to not make any adjustments to our current Materials and Methods sections.

14. I was surprised to see that a stochastic spatial model was coupled with a deterministic model assuming spatial homogeneity. It would be great to know why an ODE was used instead of a PDE for the high-copy number species.

We thank the reviewer for this thoughtful comment. We used an ODE rather than a PDE description for the high-copy-number metabolites (in our model, only the sugar galactose) because these species diffuse much more rapidly than the regulatory proteins and are present at sufficiently high abundance that spatial concentration gradients are expected to relax on timescales(1 secs) much faster than the gene-regulatory events of interest. Under these conditions, treating metabolite concentrations as spatially uniform is a reasonable approximation and substantially reduces computational cost while preserving the key regulatory dynamics.

Our goal in the hybrid RDME–ODE framework was therefore to resolve spatial effects where they are most important, namely for low-copy-number species and gene-regulatory interactions, while representing fast, abundant metabolites with a well-mixed deterministic description. We clarify this modeling assumption in the revised manuscript as follows:

“Both the transport of extracellular galactose into the yeast cell via Gal2p and galactose metabolism via Gal1p are modeled deterministically using ordinary differential equations (ODEs; see Table S11). We use a well-mixed ODE description for galactose because it is a high-copy-number metabolite that diffuses much faster than the low-copy-number regulatory proteins represented in the RDME model; thus, intracellular galactose gradients are expected to relax much faster than the gene-regulatory dynamics of interest. This hybrid RDME--ODE framework reserves spatial stochastic resolution for low-copy-number regulatory species and gene-regulatory interactions, while treating fast, abundant metabolites deterministically to reduce computational cost. ODEs are solved numerically using the LSODA function in the SciPy package in Python[42]. Because downstream metabolic pathways are not explicitly modeled, galactose-1-phosphate is represented as a dead-end species.”

15. L 192-203:In my opinion, it would be important to discuss the differences / implications beyond the acceleration. I assume that a key aspect is that GAI is no longer consumed by the reaction (which might be negligible). Yet, beyond this, it is unclear why/how the reformulation should be more efficient. Did the authors compare the simulation results? As the authors developed MGPUMpdRdmeSolver themselves, it would be great to get more insights

We thank the reviewer for this helpful comment. The benefit of the reformulation is not limited to acceleration. When a high-copy-number species such as GAI is represented explicitly in the stochastic RDME system, its large abundance generates very large propensities and therefore a very large number of reaction events. This creates a substantial computational burden, even though stochastic fluctuations in such abundant metabolites are expected to be negligible relative to those of the low-copy-number regulatory species.

In addition, the reformulation changes the treatment of the GAI--G3 binding step. In the fully stochastic description, this interaction is represented as a second-order reaction. In the hybrid formulation, because GAI is treated as a high-copy-number deterministic species, the binding step is rewritten as an effective first-order process with rate $k_{eff}=k[GAI]$ . This replaces repeated stochastic firing against an abundant reactant with a continuous deterministic coupling, which further improves efficiency.

We also clarify that we did not develop the original *MGPUMpdRdmeSolver* from scratch. The original multi-GPU RDME framework was developed previously by Hallock, Earnest, and colleagues. Our contribution in this work was to modify and extend that existing implementation for the present hybrid RDME--ODE yeast model and to improve its performance for our application. We have edited the text of the revised manuscript around line 416 to read as follows:

“Compared to prokaryotic systems, modeling yeast cells is substantially more computationally demanding because of their larger size and increased complexity. To address this, we built on the previously developed multi-GPU framework in Lattice Microbes and substantially modified *MGPUMpdRdmeSolver* for the present yeast-cell simulations[15,38]”

We agree that these changes have implications beyond speedup. In particular, GAI is no longer consumed through discrete stochastic events, and the GAI--G3 binding step is no longer simulated as an explicit second-order stochastic reaction. However, because GAI is abundant, this approximation is expected to have minimal impact on the regulatory behavior of interest while substantially improving computational tractability.

16. L. 40-42: “Although analogous regulatory switches exist in bacteria [19–21], the larger size and compartmental complexity of eukaryotic cells create spatial environments in which diffusion, organelle proximity, and intracellular trafficking can significantly influence the kinetics of regulatory interactions.” - It would be beneficial if the authors provide some scale information to support the statement. Furthermore, it is unclear whether the second part of the statement refers to an observation (which is not mentioned) or is a hypothesis. This should also be clarified.

We thank the reviewer for this insightful comment. In order to provide a scale reference and clarify that we were hypothesizing that the size and compartmental complexity of eukaryotes may affect regulatory dynamics, we have changed the sentence to the following:

“Although analogous regulatory switches exist in bacteria [19–21], the larger sizes (e.g., yeast are roughly volumetrically 100x larger than *E. coli*) and compartmental complexity of eukaryotic cells creates spatial environments in which diffusion, organelle proximity, and intracellular trafficking may significantly influence the kinetics of regulatory interactions.”

17. L. 44-45: “These differences make the galactose switch a powerful test-bed for assessing how spatial context governs regulatory outcomes.” - It is unclear if this is a hypothesis or whether this has been shown. Please clarify, e.g. by providing a reference or by restating.

We greatly thank the reviewer for this helpful comment. Our intent was not to claim that the galactose switch has already been formally established as a spatial test-bed, but rather that it is particularly well suited for this purpose. We agree that the original wording was too strong in this regard. In the revised manuscript, we have therefore restated this sentence to clarify that the budding-yeast galactose switch is a well-characterized and mechanistically rich model system for investigating how spatial context may influence regulatory outcomes. This rationale is supported by the fact that the GAL network has been studied extensively for decades as a classic model of eukaryotic gene regulation and cellular decision-making, with well-defined roles for the major regulators Gal4, Gal80, and Gal3, including compartment-dependent aspects of regulation.

“Taken together, these features make the budding-yeast galactose switch a well-suited model system for investigating how spatial context may influence regulatory outcomes(Lohr 1995, Platt 1998, Traven 2006, Stockwell 2015, Palme 2021, Hahn 2011)”

*Citations are newly added with entries provided below:*

Platt A, Reece RJ. The yeast galactose genetic switch is mediated by the formation of a Gal4p-Gal80p-Gal3p complex. EMBO J. 1998 Jul 15;17(14):4086-91. doi: 10.1093/emboj/17.14.4086. PMID: 9670023; PMCID: PMC1170741.

Lohr D, Venkov P, Zlatanova J. Transcriptional regulation in the yeast GAL gene family: a complex genetic network. FASEB J. 1995 Jun;9(9):777-87. doi: 10.1096/fasebj.9.9.7601342. PMID: 7601342.

Traven A, Jelicic B, Sopta M. Yeast Gal4: a transcriptional paradigm revisited. EMBO Rep. 2006 May;7(5):496-9. doi: 10.1038/sj.embor.7400679. PMID: 16670683; PMCID: PMC1479557.

Stockwell SR, Landry CR, Rifkin SA. The yeast galactose network as a quantitative model for cellular memory. Mol Biosyst. 2015 Jan;11(1):28-37. doi: 10.1039/c4mb00448e. Epub 2014 Oct 20. PMID: 25328105; PMCID: PMC4252300.

Julius Palme, Jue Wang, Michael Springer (2021) Variation in the modality of a yeast signaling pathway is mediated by a single regulator eLife 10:e69974

Hahn S, Young ET. Transcriptional regulation in Saccharomyces cerevisiae: transcription factor regulation and function, mechanisms of initiation, and roles of activators and coactivators. Genetics. 2011 Nov;189(3):705-36. doi:. PMID: 22084422; PMCID: PMC3213380.

18. L. 122 ff: I think it would be beneficial to also mention the diffusion part in the description. Indeed, I would be very interested in the strategy which the authors used to handle discretization effects (see e.g. doi: ).

We greatly thank the reviewer for this helpful comment. We agree that the diffusion component of the RDME and the implications of spatial discretization should be described more explicitly. In the revised manuscript, we have expanded the discussion around Eq. 1 to clarify that the RDME consists of two coupled parts: a local reaction operator, which treats each voxel as well mixed, and a diffusion-jump operator, which represents molecular transport between adjacent voxels. We have also clarified how the diffusion propensity is defined from the underlying diffusion coefficient and voxel size.

We also agree that discretization effects are important, particularly for bimolecular reactions. In our implementation, reactions are local to a voxel, so the mesh size controls both the representation of diffusion and the effective encounter statistics of reacting molecules. We will therefore add text clarifying that the voxel size must be chosen as a compromise: small enough to resolve spatial heterogeneity, but not so small that RDME bimolecular kinetics become inaccurate. In the revised manuscript, we have cited the work of Hellander et al. (2015), which discusses the scale dependence of mesoscopic reaction rates and the lower mesh-size limit for accurate RDME representations of diffusion-limited reactions.

In our case, the purpose of Eq. 1 was to introduce the governing RDME framework rather than to derive a new discretization scheme. However, we agree that the manuscript should make clearer how diffusion is represented and what assumptions underlie the chosen spatial discretization. We have revised this section accordingly and have added the relevant citation as follows:

“In the RDME, diffusion is represented as stochastic jump events between adjacent voxels with propensities determined by the species diffusion coefficient and lattice spacing, while reactions are assumed to occur only among molecules that occupy the same voxel. As a result, the voxel size affects not only spatial resolution but also the accuracy of bimolecular reaction kinetics, especially in diffusion-limited regimes (Hellander 2015).”

*Citation is newly added with entry provided below:*

Hellander S, Hellander A, Petzold L. Reaction rates for mesoscopic reaction-diffusion kinetics. Phys Rev E Stat Nonlin Soft Matter Phys. 2015 Feb;91(2):023312. doi: 10.1103/PhysRevE.91.023312. Epub 2015 Feb 23. PMID: 25768640; PMCID: PMC4854576.

19. L. 236-238: It should be clarified in which relation the current publication stands to Earnest et al. [15]. If the geometries were simply taken from a publication, this should be stated more clearly, and the section might need to be reduced. [Put 2.2.1 in SI, and reference it in 2.2]

The geometric reconstruction framework itself, including the cryo-ET-based yeast-cell model and ellipsoid-extrapolation strategy, was previously established by Earnest et al. [15]. In the present work, we used that published framework as the basis for our geometry generation, while reconstructing the additional missing cellular components like the various ER regions required for our simulations, including completion of partially imaged structures and representation of organelles not fully captured in the original tomogram. Our extension of the previously established framework used to only investigate the diffusion of an imported sugar into the nucleus Earnest et al. [15] However, modeling the galactose switch required additional information about the organelles.

To make this distinction clearer and improve the flow of the manuscript, we have shortened Section 2.2 in the main text and removed the detailed reconstruction procedure currently in Section 2.2.1, while retaining a brief summary in the main text and explicitly referencing Earnest et al. [15]. The revised manuscript now reads as follows:

## “Reconstruction of Yeast Cell Geometry Using Cryogenic Electron Tomography

The yeast-cell geometry used in this study was generated using the cryo-ET-based reconstruction framework previously described by Earnest et al.[15]. Briefly, the initial 3D geometry was derived from a cryo-FIB-milled *S. cerevisiae* cell grown in glucose-rich conditions.In the present work, we used this published framework to complete partially imaged structures, reconstruct additional cellular components required for simulation, including the chromosome, ER regions and estimate the ribosome abundances used in the model. (methods shown in below.)”

20. Section 3.5: The results suggest that spatial heterogeneity affects switching behaviour. Is this effect primarily due to rare events (e.g. first-passage times to activation) or does it also alter the steady-state distribution of active/inactive states? This distinction would be important for biological interpretation.

We thank the reviewer for this important question. In our model, the effect of spatial heterogeneity is not limited to rare events alone. Rare-event processes such as first-passage and search times are clearly important, because activation and repression require regulators to locate their targets and binding partners in space.(see our discussion in section 4.1) However, these same spatial constraints also alter effective reaction frequencies over time through compartmentalization, diffusion, and local molecular abundances, thereby changing the broader dynamical behavior of the switch rather than only isolated activation events.

That said, the present study was designed to analyze transient and intermediate-time switching behavior, not to characterize the steady-state distribution of active and inactive states. Accordingly, we did not simulate either the CME--ODE or RDME--ODE models to a point where a formal steady-state comparison could be made. Only CME-ODE/ODE versions could be integrated for a long time. We therefore limit our conclusions to transient and intermediate-time effects and have clarified this point in the revised manuscript as follows:

Line 690: “We systematically increased the spatial heterogeneity and complexity in our model to determine the effect each addition had on cellular responses to galactose uptake. Because the simulations were analyzed over finite induction windows rather than run to steady state, the results in this section are interpreted as changes in transient and intermediate-time switching behavior rather than formal differences in steady-state occupancy.”

21. Figure 4 is, in my opinion, misleading, as it appears as if subdomains are exclusive. Yet, the text refers to “overlapping regions” (l. 455).
     Indeed, I was wondering how the simulation is performed precisely, as events in the overlapping regions need to be synchronized between GPUs.

We apologize for the confusion. We agree that the original Figure 4 schematic could be misread as showing strictly exclusive subdomains, whereas in the actual implementation neighboring GPUs must exchange boundary information to resolve diffusion across subdomain interfaces. To clarify this, we have revised both the main text and the figure caption.

In the revised text, we now state that each GPU processes its assigned **primary subdomain** and exchanges **boundary-layer (apron) molecules** with neighboring GPUs during synchronization phases. We have also revised the caption of Figure 4A to make clear that the schematic shows the primary decomposition of the lattice, while neighboring GPUs communicate apron molecules at shared interfaces during synchronization steps. This clarification more accurately reflects how cross-boundary diffusion events are handled in the multi-GPU simulation.

(line -428)Text: “During synchronization phases, neighboring GPUs exchanged only the apron-layer data required to resolve particle diffusion across subdomain interfaces..”

“(A) Schematic of the multi-GPU domain decomposition used in the yeast RDME simulations. The 3D cellular lattice is partitioned into four primary subdomains, each assigned to a separate GPU; neighboring GPUs exchange boundary-layer (apron) molecules at shared interfaces during synchronization steps. (B) Performance benchmark per biological hour of 1-, 2-, and 4-GPU simulations on A100 and H100 GPUs, showing sublinear scaling with up to 2.5x speedup on four GPUs}”

22. Section 4.2.1 and some others: I find it a bit strange to have the figure indexed in the subsection heading.

We agree that including figure numbers directly in subsection headings is unnecessary and can be distracting. In the revised manuscript, we have removed figure indexing from the subsection titles and retained figure references in the main text where appropriate.

23. please check again for typos (l. 157: “from from”)

We thank the reviewer for catching this typo. We have corrected the duplicated word at line 157 (“from from”) and performed an additional careful proofreading pass to identify and fix similar typographical errors throughout the revised manuscript.

## **Reviewer 3:**

### **Major Problems:**

24. The account of the simulation methods is clear and well organized into successive stages of detail. Table 2 provides an excellent stepwise comparison.
     For each stage, can the authors provide ground-truth tests against analytic cases, or alternatively against limiting numerical cases where the solution is well-defined? This would strengthen the confidence in these methods and approximations.

We thank the reviewer for this comment. However, it is challenging to define a direct “ground truth” test in this context even for the fold changes determined at the two sugar concentations. For high (111 mM) concentration, we took the ground-truth from Paulo’s proteomics study. For low (11 mM) we took the ground-truth to be the values obtained from Ramsey’s ODE model. If the reviewer is referring to wet-lab validation, it is not experimentally feasible to construct a yeast cell without the ER or chromosomes. If “ground truth” refers instead to Ramsey’s original ODE model, that model is well mixed, so it cannot directly represent spatial structures such as chromosomes or ER. Indeed, this limitation is one of the main motivations for our study.

In response to Reviewer #1 - comment #1, we performed an ablation analysis to identify which factors contribute to the differences observed when moving from the well-mixed CME–ODE model to the spatial RDME–ODE model. This allowed us to quantify the contribution of each factor discussed in the manuscript.

In addition, we performed an interpolation analysis by gradually adding back the ER-attached ribosomes that were removed in the ribosome-competition case. Specifically, we tested four cases in which 10%, 25%, 50%, and 75% of the removed ER-attached ribosomes were restored and allowed to translate Gal2p. As expected, increasing the fraction of restored ribosomes made Gal2p abundance progressively closer to the full-ribosome case with ER.

25. It would be interesting for the authors to compare their approach with any of three other major simulators that also look at spatial stochastic events: **MCell, Smoldyn, and STEPS**. While it will be a huge exercise to replicate their entire model in another system, it would help to identify key numerical stages in their approach which could be compared. Clearly the single-partical reaction-diffusion calculations will run much slower, but a cross-test of a short portion of the calculation would be reassuring.

We thank the reviewer for this thoughtful suggestion. We agree that comparisons with MCell(https://mcell.org/), Smoldyn(https://www.smoldyn.org), and STEPS (Visintin C, et al. steps: Software for spatially and temporally explicit population simulations. Methods Ecol Evol. 2020;11:596–603.) would be valuable in principle. These are mature and widely used spatial stochastic simulators that have made important contributions to cell-scale modeling. MCell is particularly strong for particle-based Monte Carlo simulations in realistic three-dimensional cellular geometries. Smoldyn provides an efficient particle-based framework for simulating individual molecules, diffusion, reactions, membranes, and spatial confinement. STEPS is especially powerful for stochastic reaction--diffusion simulation on tetrahedral meshes, with important applications to complex cellular geometries and neuronal signaling.

However, a full reimplementation of our model in another simulator would be a substantial undertaking because our framework combines a reconstructed yeast geometry, hybrid RDME--ODE coupling, compartment-specific initialization, ER-constrained translation, and limited-ribosome competition. Some of the large-system simulations presented here are already computationally demanding in our own framework and would not be straightforward to reproduce in alternative simulators.

To improve transparency and enable practical cross-checking, we have prepared SBML representations of both the well-mixed CME--ODE model and a spatially decompartmentalized RDME--ODE version of the model. The CME--ODE SBML captures the reaction topology, compartments, and hybrid deterministic/stochastic structure of the well-mixed system, while the RDME--ODE SBML includes compartmental subdivision and annotations linking species and reactions to the spatially resolved model. These resources allow readers to inspect, reuse, and reproduce simplified versions of the model in other compatible software environments.

We agree that providing these model specifications is a useful step toward transparency and portability, even if the full geometry-aware hybrid implementation is not directly transferable to standard external simulators. We have therefore revised the manuscript accordingly and have made both SBML files available with the model resources (<https://github.com/Luthey-Schulten-Lab/Yeast_Galactose_Switch_RDMEODE/tree/main/SBML_model>).

26. Similarly, when the authors break the problem up over multiple GPUs, can they illustrate a validation step to show that the result matches single-GPU or even single-thread outcomes?

We greatly thank the reviewer for this important suggestion. We agree that validation of the multi-GPU implementation against smaller-scale reference calculations is important. The underlying multi-GPU RDME framework used here has already been developed and validated in previous Lattice Microbes publications, including the original multi-GPU implementation paper by Hallock et al., which presents parallel performance results for reaction-diffusion simulations on multiple GPUs, and the later yeast cryo-ET modeling study by Earnest et al., which applied the same framework in a biologically realistic eukaryotic setting. (<https://pubs.acs.org/doi/full/10.1021/acs.jpcb.7b00672> <https://doi.org/10.1016/j.parco.2014.03.009>)

In the present work, our contribution was to adapt and extend that previously validated framework for the current hybrid RDME--ODE yeast galactose-switch model, rather than to introduce a new parallel stochastic engine from scratch. We have clarified this point in the revised manuscript and cite these prior validation studies more explicitly when describing the multi-GPU solver.

### **Minor Problems:**

27. While I acknowledge that it is difficult to encode all the model details into SBML, it would help to have an SBML specification of their reaction systems, ideally with the compartmental subdivisions. This provides readers with access to several tools at least for the reaction topology and the CME and well-mixed versions of the model.

We thank the reviewer for this helpful suggestion. We agree that providing SBML specifications would improve transparency and interoperability, even if not all details of the full spatial hybrid implementation can be captured in a standard SBML representation. To address this, we have provided SBML files for both the well-mixed CME--ODE model and a spatially decompartmentalized RDME--ODE version of the model. The CME--ODE SBML includes the reaction topology, kinetic parameters, and compartment structure of the well-mixed system, while the RDME--ODE SBML includes compartmental subdivisions and spatial annotations corresponding to the compartment-resolved model structure.

These SBML resources will allow readers to inspect the reaction topology and reproduce the CME/well-mixed and compartmentalized non-geometry-resolved versions of the model in other compatible software environments. We agree that this is a useful way to make the model more accessible, and we have revised the manuscript Data Availability section as follows:

“All simulation and analysis code, with SBML files for CME-ODE and RDME-ODE model configurations used in this study is publicly available at GitHub. (public_luthey-schulten-labyeast_galactose_switch_rdmeode_2025)”

28. The account of parameterization is painstaking, for which I compliment them, but the number of items makes it difficult to follow. Can the authors provide a summary table of what they did for each parameter, to help the reader?

We thank the reviewer for this helpful suggestion and for the positive feedback on the level of detail in the Supporting Information. We agree that the current parameterization description is difficult to follow in narrative form.

The key point is that the core reaction-network parameterization was not newly fit in this study. Rather, the CME--ODE parameterization was established in prior work and was carried over here into the RDME--ODE framework. In the present study, our contribution was to transfer that existing parameterization into the spatial hybrid model and to make the model-specific adjustments required for the RDME--ODE formulation, such as removing or reformulating terms associated with the spatial implementation and adding diffusion/transport-related quantities where needed.

To make this clearer, we have added a summary Table S1 in Subsection S1.1 to the Supporting Information that serves as a roadmap for the reader. This table does not duplicate the full numerical listings already present, but rather summarizes, for each parameter group and reaction class, whether it was inherited from the prior CME--ODE model, retained unchanged, reformulated in the RDME--ODE framework, or introduced specifically for the spatial model.

29. Section 4.1: Point ii. I'm puzzled at this comment. One normally takes bulk rates as a staring point to estimate propensities, in this process the voxel
     volumes are explicitly accounted for, and in the limit of many small voxels
     comprising a single large volume the two converge. Can the authors clarify?
     It is true that there are higher-order effects due to different local
     propensities, but this does not seem to be what the authors refer to.

In Section 4.1, point ii, we argue that the reaction propensities do not converge at the limit of many subvoxels becoming one large system volume. The reasoning behind the incongruence is described on lines 760-768. In essence, if we consider a system with identical molecular counts, but one is partitioned into subvoxels and the other is a single subvoxel, summing up the collective individual subvoxel propensities is not equivalent with the large single volume propensity (if the reaction is second-order). This is because in the single voxel volume system, all molecules are able to react with each other, whereas, in the many subvoxel system, only select copies of each reacting molecule are allowed to interact. For a mathematical treatment of this comparison, please see SI, Section 5.
