# Supplementary material for: Spatially resolved reaction–diffusion modeling reveals effects of intracellular spatial heterogeneity on yeast galactose network dynamics
Source: PLoS Comput Biol. 2026 Oct 5;22(10):e1014811. doi: 10.1371/journal.pcbi.1014811 (PMC13705588; doi:10.1371/journal.pcbi.1014811)
Supplement: Supplementary file 3 [file pcbi.1014811.s010.docx]

# Response to Reviewer#3 Comments

We greatly appreciate the efforts made by both editor and reviewers, especially the additional comments and questions Reviewer #3 raised in reading the revised manuscript. We apologize for the lack of citations to some of the articles that were foundamental in developing algorithmic treatments of the kinetics of biological processes described by the reaction diffusion master equation. Hopefully, we have now addressed all the concerns.

Reviewer #3:

1. Major point 1: Ground truth tests.
   I understand that it is difficult to find ground-truth experiments. By this comment I meant a simulation of an analytically well-defined reaction-diffusion system, not a new experiment. I make a similar suggestion below for validation against other simulators.

We thank the reviewer for clarifying this point. We now understand that the request was not for new experimental validation, but for validation against analytically defined reaction–diffusion systems or comparison with other spatial stochastic simulators.

We agree that such tests are important for RDME-based simulation frameworks. In the present manuscript, our main goal was to study how diffusion, compartmentalized geometry, ER-associated translation, ribosome localization, and other spatial heterogeneities affect yeast galactose-switch kinetics. Therefore, we focused on the biological consequences of introducing spatially resolved RDME–ODE modeling into a previously studied GAL regulatory network, rather than presenting a full technical revalidation of the Lattice Microbes RDME solver that had partially been considered in previous articles.

The RDME implementation used here builds directly on previously validated versions of Lattice Microbes. Earlier Lattice Microbes work tested analytically tractable reaction systems, including reversible second order/first-order reactions such as $A+B\rightleftharpoons C$, and compared the framework with other mesoscopic stochastic simulators and Gillespie-type methods, including MesoRD and next-subvolume approaches.

The underlying Lattice Microbes methodology and earlier benchmark examples are described in the ***Journal of Computational Chemistry*** ***2012*** technical article by Roberts, Stone, and Luthey-Schulten.

<https://doi.org/10.1002/jcc.23130> (Performance comparisons are given Table 1, provided below for accuracy of RDME check SI section 1.2; for CME, seen below).

Computational performance comparisons of a reversible bimolecular reaction and the Lac switch reaction system are given in Table 1 of the original **JCS** article and for convenience are also provided below. Analyses of the accuracy of both the CME and RDME solvers used in Lattice Microbes on reversible bimolecular reactions are given in the original article’s Supporting Information, and for convenience are also given below in Figures S4 and S5.

**Figure S4. Bimolecular Reaction Accuracy Analyses for CME Treatment in Lattice Microbes**

**Figure S5. Bimolecular Reaction Accuracy Analyses for RDME Treatment in Lattice Microbes**

Table 1 indicates that the computational performance of Lattice Microbes solvers is comparable or outperforms existing solvers. Figures S4 and S5 indicate that, for a bimolecular reaction, the Lattice Microbes CME solver is able to reproduce analytical solutions to the chemical master equation and that the RDME solver is able to accurately reproduce the behavior of the CME solver.

The same Lattice Microbes RDME framework was also applied to the spatial Lac switch model in *E. coli* in Roberts et al. ***Plos Computational Biology*** ***2011*** where simulation results were compared with experimental measurements: <https://doi.org/10.1371/journal.pcbi.1002010>.

Additionally, the same core RDME framework was used in our previous study Earnest et al. ***Journal Physical Chemistry B, 2017*** yeast spatial modeling work to simulate sugar import and diffusion through the cytosol and nucleus via nuclear pores: <https://doi.org/10.1021/acs.jpcb.7b00672>. As this article was part of the Festschrift publication for Klaus Schulten, who had just passed away, authors only gave a brief interpretation of the results and did not compare it to the 2013 study from the Isaacson lab that had treated the diffusion of a sugar in yeast into the nucleus. The citation is now given in the Introduction along with the citations to the 2017 **Journal of Physical Chemistry B (JCPB)** article and 2018 Bianchi et al. **IEEE Systems Biology** where Galactose Switch in yeast was simulated with Lattice Microbes software using a combined stochastic (CME) treatment of gene expression and a deterministic (ODE) treatment of the sugar uptake and first steps of processing due to the high concentrations of galactose. The 2018 Bianchi et al. article investigated the errors associated with communication times between the CME and ODE solvers which were critical for this present manuscript where we have added the spatial and diffusion effects through the RDME treatment.

We are preparing a manuscript about all the improvements in Lattice Microbes code and algorithms that have taken place in the last year where we will provide additional tests of its computational methods on a few models already published in the literature. This is a major undertaking that goes beyond the scope of this present manuscript on the galactose switch in yeast, but to provide Reviewers a sense of the scope of the testing, toy models, and improvements being considered, we have provided a slide summarizing part of the new study. We hope this new study along with the existing comparisons provided in the above Table and Figures will satisfy Major Points 1 and 2 from Reviewer #3.

2. Major point 2: Reimplementation.
   I appreciate the author's preparation of an SBML representation. That very substantially improves the accessibility and reusability of their model. I understand it is hard to reimplement the entire thing (though with AI,who knows!). But coming to the point below about the RDME, might they do some tests of a simple 'toy' reaction-diffusion system to compare against other simulators, specially particle-based onies? There are also extant RDME simulators, mentioned below.

For toy model, in the same yeast geometry, there were some simulation done in previous studies: <https://pubs.acs.org/doi/pdf/10.021/acs.jpcb.7b00672>

We thank the reviewer for this constructive suggestion and for noting the value of the SBML representation. We agree that simple benchmark systems are useful for establishing confidence in the numerical behavior of reaction–diffusion simulators, especially when comparing RDME implementations with alternative spatial stochastic approaches.

As we discussed above, several such “toy-model” validation in Earnest et al. JPCB 2017 and benchmarking tests have already been reported in the original Lattice Microbes benchmarking paper by Roberts, Stone, and Luthey-Schulten in JCC (2013). In JPCB 2017 we used an early yeast tomogram from our experimental collaborators in which the ER was not resolved, subsequent FIB-SEM has allowed more detailed analysis of the organelles used in this manuscript. Additionally in 2017, we only considered sugar diffusion through the cytoplasm into the nucleus, but in the present manuscript on the galactose switch, after import the galactose is either metabolized or binds in the cytoplasm to the first protein in the galactose regulatory network, and never diffuses into the nucleus. So a direct comparison to the present manuscript is not possible for sugar diffusion. In the technical article in JCC 2012, Lattice Microbes was evaluated on a reversible bimolecular reaction–diffusion system, including comparisons between the approximate MPD-RDME method and exact next-subvolume sampling, with error assessed over large trajectory ensembles. The same study also compared RDME performance against other available simulators, including MesoRD, using both simple reaction–diffusion systems and lac-switch cellular models. (See above Table from the JCC article).

**Therefore, while we agree that a broader cross-simulator benchmark, particularly including particle-based methods, would be valuable, we view such a systematic comparison as a substantial methodological study in its own right rather than a necessary component of the present biological application. In the revised manuscript, we have clarified this point and cite the previous Lattice Microbes and yeast-geometry benchmarking studies to better contextualize the RDME implementation and its prior validation.** **We are *preparing a manuscript about all the improvements in Lattice Microbes code and algorithms* that have taken place in the last year where we will provide additional tests of its computational methods on a few models already published in the literature. This is a major undertaking that goes beyond the scope of this present manuscript on the galactose switch in yeast, but to provide Reviewers a sense of the scope of the testing, toy models, and improvements being considered, we have provided a slide summarizing part of the new study (check preliminary work below : GPU_based_GillspieSolver_Benchmarking). We hope this new study along with the existing comparisons provided in the above Table will satisfy Major Points 1 and 2 from Reviewer #3.**

**Results zoom in:**

3. Point: RDME

   This turns out to be a complex issue at the heart of their RDME method.

   3.1-> I'll start with pointing out previous work which addresses this:
   The sections 4.1.2 and 4.1.3 look to me like a restatement of the
   work on the problem of failure of RDME methods in the microscopic limit,
   which is discussed in
   Isaacson and Isaacson 2009 (Phys Rev E),
   Isaacson 2013 (J Chem Phys), and
   Gillespie, Petzold and Seitaridou 2014 (J Chem Phys).
   The authors have mentioned the work of Hellander et al (2015) which follows
   up from these. So it isn't clear to me what new insights these two sections
   (4.1.2 and 4.1.3) bring. In any event, can the authors open these sections by
   placing them clearly in context of the previous work?

   We agree that the microscopic-limit failure of the standard local RDME is well established and should not be presented as a new theoretical result and have now cited all four articles together in our manuscript. We also identified a related study by Isaacson et al. (2013), “The Influence of Spatial Variation in Chromatin Density Determined by X-Ray Tomograms on the Time to Find DNA Binding Sites,” which examines how spatial chromatin-density variation and volume exclusion affect the time required for proteins to locate DNA binding sites. We have now cited this work as well. Accordingly, we revised Sections 4.1.2 and 4.1.3 to explicitly acknowledge the relevant prior literature and to clarify that our contribution is the biological interpretation of finite-voxel RDME locality in the GAL promoter-binding model, rather than a new analysis of RDME convergence.

   added the following paragraph at the beginning of Section 4.1.2:

   “In the RDME framework, diffusion is represented by jumps between voxels and bimolecular reactions occur only when reactants are locally co-located. This finite-voxel interpretation is important because prior work has shown that the standard local RDME does not converge to a microscopic Smoluchowski- or Doi-type model as voxel size approaches zero; instead, bimolecular reactions can be lost when the mesh is made too fine, implying a finite range of mesoscopic validity[89-92]. We therefore use the RDME here as a finite-resolution spatial model to examine how molecular search and local co-localization affect promoter-binding events.

   The inclusion of spatial heterogeneity introduces the need for reacting molecules to find one another.Similar voxel-based models of nuclear protein search have shown that chromatin organization and volume exclusion can alter the time required for a protein to locate a specific DNA binding site [88].“

   3.2-> I should also mention that the lattice diffusion model seems reminiscent of
   NeuroRD, which uses a similar approach by Oliveira et al 2010, PMID: 20661441
   There is also the Spatial Gillespie method (Next Subvolume Method 2014) and
   MesoRD (Fange et al 2012, Bioinformatics).
   Can the authors comment on how the logic of these existing tools for
   handling propensity differs from their implementation of the RDME approach?

   We thank the reviewer for pointing out NeuroRD which treated processes in neurons, the Spatial Gillespie/Next Subvolume Method, and MesoRD. These methods are closely related because they are all implementations of the mesoscopic subvolume/RDME framework. In this framework, the spatial domain is divided into subvolumes; each subvolume is treated as locally well mixed; reactions are evaluated from local molecule counts within each subvolume; and diffusion is represented as stochastic transfer between neighboring subvolumes. The Next Subvolume Method samples this process by assigning each subvolume a next event time, selecting the subvolume with the earliest event, and then using a Gillespie direct-method step within that subvolume to determine whether the event is a reaction or diffusion event.

   Our implementation follows the same local RDME propensity logic, but differs in the sampling algorithm. Specifically, we used Lattice Microbes with its MPD-RDME solver rather than an exact Next Subvolume Method. In MPD-RDME, diffusion and reaction operators are applied over short time steps using operator splitting. Within each subvolume, reaction propensities are still computed from local molecule counts, and reaction events are selected using the standard Gillespie direct-method logic. Thus, the difference is not a new propensity law, but the use of the GPU-accelerated MPD-RDME sampling scheme within our whole-cell yeast geometry and hybrid RDME--ODE GAL model.

   We have revised the Methods section to clarify this distinction and to cite NeuroRD, the Spatial Gillespie/Next Subvolume Method, MesoRD, and Lattice Microbes as related subvolume-based RDME implementations. We also now mention approximate accelerated variants, including tau-leaping-based subvolume methods, which use larger time increments to sample multiple reaction or diffusion events approximately rather than sampling every event exactly.

   In section 2.1.2: “The reaction-diffusion master equation (RDME) provides … spatially-resolved models. Our RDME implementation follows the standard local-propensity formulation: reactions are evaluated within locally well-mixed subvolumes, while diffusion is represented as stochastic transfer between neighboring subvolumes. In Lattice Microbes, reaction events within each subvolume are sampled using direct Gillespie logic. Other RDME simulators use related sampling strategies, including the Next Subvolume Method and approximate tau-leaping variants, but these differ mainly in how events are sampled rather than in the underlying local RDME propensity formulation[46-50].”

In addition, to more clearly define the scope of the present study and the improvements that will be required for a more comprehensive spatial whole-cell model, we have substantially revised Sections 4.3, **Limitations and Future Directions**, and 4.4, **Conclusions**. Section 4.3 now explicitly discusses the computational cost and limited number of stochastic replicates, the use of static cellular architectures, the absence of explicit organelle remodeling and vesicle-mediated trafficking, the reduced representation of the Leloir pathway, and the simplified treatment of post-transcriptional and post-translational processes. We also now emphasize the need for further optimization of the Lattice Microbes simulation code as additional cellular processes are incorporated. Section 4.4 has been revised to more clearly summarize the principal contribution of the present study: that incorporating experimentally derived cellular and chromosome geometries into a spatially resolved yeast model can substantially alter predicted gene activation, transporter production, and nutrient uptake through changes in diffusion, molecular encounter probabilities, and accessibility. We have also clarified that this work represents an important first step toward a spatially resolved whole-cell model of budding yeast and highlights both the opportunities and challenges associated with extending whole-cell modeling to eukaryotic cells. These revisions are highlighted in red in the revised manuscript.

3.3-> I worked through the logic of Supplementary section 5.
It is all straightfoward till the second last line, where the
author asserts (without clarity) that SB,j/SB ~ 1/300.

I would have gone with the logic that
* there is abundance SB in the cell
* The cell is subdivided into N subvoxels.
-> Therefore mean abundance of B (integrated over time) in one subvoxel =
SB,j = SB/N.

I do not understand how the authors get SB,j/SB ~ 1/300, which seems to
be the basis for the assertion that the propensity differs between CME and
RDME. My reading is that this may be a patch for a case where RDME fails to
work well in the microscopic limit. Can the authors comment?

We thank the reviewer for identifying this ambiguity. We agree that the original estimate, S_B,j / S_B ≈ 1/300, was insufficiently justified because it conflated the smallest nonzero local encounter state, S_B,j = 1, with the time-averaged occupancy of the promoter-containing voxel.

Under a well-stirred approximation with N equivalent nuclear voxels:

E[S_B,j | A = 1] = (1/N) E[S_B,nuc | A = 1],

or more generally:

E[S_B,j | A = 1] = (V_j / V_nuc) E[S_B,nuc | A = 1].

This relation requires spatially uniform sampling of G80d within the accessible nuclear volume. Because the RDME distribution is spatially and temporally nonstationary, this assumption is not guaranteed. We therefore removed the 1/300 approximation and no longer infer promoter-voxel occupancy from total nuclear abundance.

The revised repression propensities are:

P_CME^rep(t) = [k_rep / (N_A V_cell)] A_CME(t) S_G80d,nuc^CME(t),

and

P_RDME^rep(t) = [k_rep / (N_A V_j)] A_RDME(t) S_G80d,j^RDME(t),

where S_G80d,j^RDME is the G80d copy number in the promoter-containing voxel. The active-state-conditioned propensity ratio is:

R_rep^local(t) = (V_cell / V_j) ×
E[S_G80d,j^RDME(t) | A_RDME(t) = 1] /
E[S_G80d,nuc^CME(t) | A_CME(t) = 1].

Thus, the revised comparison directly uses the local RDME promoter-voxel occupancy and the nuclear G80d abundance used in the CME implementation, without assuming S_B,j / S_B = 1/N. For a 28 nm RDME voxel:

V_cell / V_j = 33.3 μm³ / (0.028 μm)³ ≈ 1.517 × 10⁶.

We also analyzed the saved RDME spatial lattices at one-second resolution. Most promoter-voxel occupancies were obtained directly from these snapshots. However, following dissociation from DG2_G4d_G80d to DG2_G4d, the released G80d molecule may leave the promoter voxel before the next snapshot. To represent this unresolved immediate post-dissociation state, we assigned one local G80d molecule to the activated snapshot associated with each observed dissociation event.

Seven release-associated events were identified, of which three had sufficient active CME trajectory support to produce defined propensity ratios. These values are explicitly labeled **release-approximated**, rather than directly observed one-second residence events.

This approximation is applied only during post-processing and does not modify the RDME simulation, reaction rates, or microscopic dynamics. The revised analysis therefore removes the unsupported 1/300 assumption and directly evaluates the difference between local RDME repressor availability and the globally represented CME abundance. Supplementary Section 5 and the corresponding figure have been revised accordingly. ( figure shown as below)

**Figure S17. Release-approximated local RDME/CME repression-propensity ratio**. Five RDME and fifty CME replicates were analyzed, with all quantities conditioned on the promoter being active, $\hat{\boldsymbol{E}}[\cdot|A^{X}=1]$(A) Repressor occupancy in the RDME model (upper) and nuclear G80d abundance in the CME model (lower), reported in molecules. Each point represents the ordinary mean across promoter-active replicates, with active replicates lacking a repressor counted as zero. Thus, one occupied promoter voxel among *n* active RDME replicates corresponds to an average occupancy of 1/*n*. Because local RDME occupancy is binary (zero or one molecule), the upper curve also represents the local-encounter frequency and is not repeated in panel C. (B) The local repression-propensity ratio, $R_{rep}^{local}(t)$ calculated from the RDME and CME quantities shown in panel A using the repression-propensity ratio defined above. Values are shown only at time points for which at least one of the five RDME replicates and four of the fifty CME replicates have an active promoter; unsupported time points are left blank rather than assigned a value of zero. The vertical axis uses a symmetric logarithmic scale that is linear below unity, allowing exact zeros to remain visible. (C) Numbers of promoter-active RDME and CME trajectories contributing to each time point. The release approximation assigns one local G80d molecule to the activated snapshot associated with each saved DG2_G4d_G80d → DG2_G4d transition. Seven such release events were identified, three of which occurred at time points with sufficient CME support for calculation of the ratio. ( 5 RDME trajectories and 20 CME trajectories are used in the study.)

Abner – put in the figure caption. Figure b and c are showing the time dependent expectation values indicated in red in the upper right hand corner of the figure. Also state that the study used 5 RMDE and 20 CME replicate runs.
