# Supplementary material for: Spatially resolved reaction–diffusion modeling reveals effects of intracellular spatial heterogeneity on yeast galactose network dynamics
Source: PLoS Comput Biol. 2026 Oct 5;22(10):e1014811. doi: 10.1371/journal.pcbi.1014811 (PMC13705588; doi:10.1371/journal.pcbi.1014811)
Supplement: Peer review history

# Peer review: Decision Letter 0

**DOI:** 10.1371/journal.pcbi.1014811.r001

**Authors:** Marc R Birtwistle

7 Apr 2026

Spatially resolved reaction diffusion modeling reveals effects of intracellular spatial heterogeneity on  yeast galactose network dynamics

PLOS Computational Biology

Dear Dr. Luthey-Schulten,

Thank you for submitting your manuscript to PLOS Computational Biology. After careful consideration, we feel that it has merit but does not fully meet PLOS Computational Biology's publication criteria as it currently stands. Therefore, we invite you to submit a revised version of the manuscript that addresses the points raised during the review process.

Please submit your revised manuscript by Jun 07 2026 11:59PM. If you will need more time than this to complete your revisions, please reply to this message or contact the journal office at ploscompbiol@plos.org. When you're ready to submit your revision, log on to https://www.editorialmanager.com/pcompbiol/ and select the 'Submissions Needing Revision' folder to locate your manuscript file.

Please include the following items when submitting your revised manuscript:

* A letter that responds to each point raised by the editor and reviewer(s). You should upload this letter as a separate file labeled 'Response to Reviewers'. This file does not need to include responses to formatting updates and technical items listed in the 'Journal Requirements' section below.

* A marked-up copy of your manuscript that highlights changes made to the original version. You should upload this as a separate file labeled 'Revised Manuscript with Track Changes'.

* An unmarked version of your revised paper without tracked changes. You should upload this as a separate file labeled 'Manuscript'.

If you would like to make changes to your financial disclosure, competing interests statement, or data availability statement, please make these updates within the submission form at the time of resubmission. Guidelines for resubmitting your figure files are available below the reviewer comments at the end of this letter

We look forward to receiving your revised manuscript.

Kind regards,

Marc Birtwistle

Section Editor

PLOS Computational Biology

**Additional Editor Comments:**

The reviewers communicate excitement for the work but also bring up substantial points to improve the manuscript that should be thoroughly addressed in a revision. Rev 1 particularly notes some potential improvement relative to the biological focus and findings.

**Journal Requirements:**

If the reviewer comments include a recommendation to cite specific previously published works, please review and evaluate these publications to determine whether they are relevant and should be cited. There is no requirement to cite these works unless the editor has indicated otherwise.

1) Please ensure that the CRediT author contributions listed for every co-author are completed accurately and in full.

At this stage, the following Authors/Authors require contributions: Abner T. Apsley, Emmy Earnest, Julia Mahamid, Marie-Christin Spindler, Zane Thornburg, Tianyu Wu, and Zaida Luthey-Schulten. Please ensure that the full contributions of each author are acknowledged in the "Add/Edit/Remove Authors" section of our submission form.

The list of CRediT author contributions may be found here: https://journals.plos.org/ploscompbiol/s/authorship#loc-author-contributions

2) We ask that a manuscript source file is provided at Revision. Please upload your manuscript file as a .doc, .docx, .rtf or .tex. If you are providing a .tex file, please upload it under the item type u2018LaTeX Source Fileu2019 and leave your .pdf version as the item type u2018Manuscriptu2019.

3) Please upload all main figures as separate Figure files in .tif or .eps format. For more information about how to convert and format your figure files please see our guidelines:

https://journals.plos.org/ploscompbiol/s/figures

4) We have noticed that you have uploaded Supporting Information files, but you have not included a list of legends. Please add a full list of legends for your Supporting Information files after the references list.

5) Please note that your Data Availability Statement is currently missing the DOI/accession number of each dataset OR a direct link to access each dataset. If your manuscript is accepted for publication, you will be asked to provide these details on a very short timeline. We therefore suggest that you provide this information now, though we will not hold up the peer review process if you are unable.

6) Please amend your detailed Financial Disclosure statement. This is published with the article. It must therefore be completed in full sentences and contain the exact wording you wish to be published.

1) State the initials, alongside each funding source, of each author to receive each grant. For example: "This work was supported by the National Institutes of Health (####### to AM; ###### to CJ) and the National Science Foundation (###### to AM)."

2) State what role the funders took in the study. If the funders had no role in your study, please state: "The funders had no role in study design, data collection and analysis, decision to publish, or preparation of the manuscript."

3) If any authors received a salary from any of your funders, please state which authors and which funders..

If you did not receive any funding for this study, please simply state: u201cThe authors received no specific funding for this work.u201d

7) Please ensure that the funders and grant numbers match between the Financial Disclosure field and the Funding Information tab in your submission form. Note that the funders must be provided in the same order in both places as well.

8) Kindly amend your competing statement in the online submission form to align with the journal's style guidelines: 'The authors declare that there are no competing interests.'

**Reviewers' comments:**

Reviewer's Responses to Questions

**Comments to the Authors:**

**Please note here if the review is uploaded as an attachment.**

Reviewer #1: In this manuscript, Wu and colleagues present a computational model of yeast which focuses on galactose regulatory network but incorporates many spatial features and characteristics. It’s exciting to see the latest work from the Schulten Lab, and I appreciate the effort that has gone into this study. I see it as a strong candidate for PLOS CB, but also have some suggestions, mostly about ways in which they could perform further perturbations on their simulations to test some of their hypotheses, and also going deeper with regard to biological insight and relevance to the yeast community.

In Result 3.1, it is shown that there is a difference between the well-stirred model and the model which incorporates spatial aspects, with the conclusion that such incorporation can lead to shifts in the timing. I would have loved to see more depth on what specifically is causing the models to have different results. With regard to the latter, an explanation is given but it would be great to use model perturbation to illustrate this more clearly.

Result 3.2 focuses on adding a detailed pre-determined physical chromosome to the simulations. I would have thought that this would lead to a substantial difference in the simulation outcomes, due to the specific localization of the genes involved as well as the diffusion obstacles they mention. However, they don’t observe a difference, which is attributed to the concentrations of activators and repressors in the nucleus. It therefore seems straightforward to perform simulations in which these concentrations are increased, to try and find concentrations in which adding the physical aspects of the chromosome would matter.

Result 3.5.1, we now see which model is now closest to experimentally-measured behaviors, but we definitely need some more detail here. For example, the impression one gets from Result 3.1 is that the comparisons between CME-ODE and RDME-ODE results and experiments should be similar. Then of course, there is the finding that even the Ramsey ODE model outperforms everything when it comes to Gal80p and that the most integrated model performs among the worst with regard to Gal3p. These need significantly more discussion. What could be done to get the most integrated model to match more correctly? Some treatment of this topic is given in Result 3.5.2, with mention of adding a inhibitory link between Gal1p and Gal80p. It seems like this would be an addition to the model that would add value to this specific study, and I can’t envision a future paper in which only this is added, so I would recommend performing those simulations and giving the results and analysis here.

Another perturbation that could be simulated is suggested by a conclusion in 4.2.1: “We therefore expect gene positioning to become more influential in regimes with slower nucleoplasmic diffusion or stronger crowding.” Again, I recommend testing this hypothesis in simulation.

Finally, overall it feels like the manuscript should be more focused on the biological relevance and findings that were or could be generated from this work. The conclusions and discussion are more focused on the modeling side. What are the takeaways for a yeast biologist? Should they be using this model, even though some of the comparisons to experiment are not as close as others? If so, what are the main justifications for doing so?

Minor

Lines 518-523: confusing, I would rewrite and probably use multiple sentences

Line 576: should be “Next, the converted”

Legend of Fig. 9: two periods at the end

Reviewer #2: The manuscript “Spatially resolved reaction diffusion modeling reveals effects of intracellular spatial heterogeneity on yeast galactose network dynamics” by Wu et al. presents a spatially resolved model of the galactose switch in budding yeast. The hybrid model employs a reaction–diffusion master equation for the description of gene regulation and an ODE model for the description of metabolic and transport processes. The results of this advanced simulation model are qualitatively compared to the results of models assuming spatial homogeneity.

The model proposed by the authors is impressive, and there are few models which reach the same depth. It allows for the analysis of the impact of spatial structure on the time evolution of its state, and might be extended to study perturbations. Assuming that the code is fully available, this could be an interesting basis for further research. Yet, that said, I think that the description of the comparison between different models, in particular spatially resolved and homogenous) could be improved. Furthermore, the need / benefit of the parallelisation does not become completely clear, and a sensitivity analysis would be very beneficial.

Major:

- Some parts of the methods section read like an Introduction rather than a Materials & Methods section, e.g. Section 2.1.2. In my opinion, a Methods section should describe what has been used, not what could be used / is available. I would recommend integrating the respective section with the actual description of the work.

- I was surprised to see that a stochastic spatial model was coupled with a deterministic model assuming spatial homogeneity. It would be great to know why an ODE was used instead of a PDE for the high-copy number species.

- L. 192-203: In my opinion, it would be important to discuss the differences / implications beyond the acceleration. I assume that a key aspect is that GAI is no longer consumed by the reaction (which might be negligible). Yet, beyond this, it is unclear why/how the reformulation should be more efficient. Did the authors compare the simulation results? As the authors developed MGPUMpdRdmeSolver themselves, it would be great to get more insights.

- Figure 4: The scaling behavior appears rather suboptimal, e.g. from 2 to 4 H100 GPUs, the runtime is only reduced by ~25%. Given the fact that one has to perform multiple runs anyway, I am wondering whether a parallelization-free code would not have been effectively more computationally efficient. This should be discussed.

- L. 502 ff: I assume that these results depend massively on the model parameterization. In this case, it would be critical to know how the CME-ODE was parameterized in comparison to the RDME-ODE. As collision rates are volume dependent, the conversion of the parameter does not appear trivial. The geometry effect could simply be due to a suboptimal parameter “conversion”.

Overall, the comparison between spatially resolved and homogeneous models remains largely qualitative.

- Given the complexity of the model, a sensitivity analysis would have been valuable.

- Section 4: In parts, this is not really a discussion but presents new results or at least discusses new aspects, e.g. Section 4.1.2 - 4.1.5. I would recommend that the authors consider a redistribution of the content.

Minor:

- L. 40-42: “Although analogous regulatory switches exist in bacteria [19–21], the larger size and compartmental complexity of eukaryotic cells create spatial environments in which diffusion, organelle proximity, and intracellular trafficking can significantly influence the kinetics of regulatory interactions.” - It would be beneficial if the authors provide some scale information to support the statement. Furthermore, it is unclear whether the second part of the statement refers to an observation (which is not mentioned) or is a hypothesis. This should also be clarified.

- L. 44-45: “These differences make the galactose switch a powerful test-bed for assessing how spatial context governs regulatory outcomes.” - It is unclear if this is a hypothesis or whether this has been shown. Please clarify, e.g. by providing a reference or by restating.

- L. 122 ff: I think it would be beneficial to also mention the diffusion part in the description. Indeed, I would be very interested in the strategy which the authors used to handle discretization effects (see e.g. doi: 10.1103/PhysRevE.91.023312).

- L. 236-238: It should be clarified in which relation the current publication stands to Earnest et al. [15]. If the geometries were simply taken from a publication, this should be stated more clearly, and the section might need to be reduced.

- Section 3.5: The results suggest that spatial heterogeneity affects switching behaviour. Is this effect primarily due to rare events (e.g. first-passage times to activation) or does it also alter the steady-state distribution of active/inactive states? This distinction would be important for biological interpretation.

- Figure 4 is, in my opinion, misleading, as it appears as if subdomains are exclusive. Yet, the text refers to “overlapping regions” (l. 455).

Indeed, I was wondering how the simulation is performed precisely, as events in the overlapping regions need to be synchronized between GPUs.

- Section 4.2.1 and some others: I find it a bit strange to have the figure indexed in the subsection heading.

- please check again for typos (l. 157: “from from”)

Reviewer #3: This is an interesting, thorough, and potentially foundational study of

yeast galactose signaling, with the development of a spatial model for

genetic control and protein synthesis. It promises to be useful for

future high-resolution spatial and structural models of yeast genetic

and metabolic signaling.

My comments are from this point of view: How can one strengthen the

confidence in this spatially detailed and numerically challenging simulation?

How can one provide an easy, stepwise progression from well-stirred models

to the fully elaborated model of the authors, and what does one gain from

each level of detail? I do not address the completeness of the chemical

and cell biological model.

Major comments.

1. The account of the simulation methods is clear and well organized into

successive stages of detail. Table 2 provides an excellent stepwise comparison.

For each stage, can the authors provide ground-

truth tests against analytic cases, or alternatively against limiting numerical

cases where the solution is well-defined? This would strengthen the confidence

in these methods and approximations.

2. It would be interesting for the authors to compare their approach with any

of three other major simulators that also look at spatial stochastic events:

MCell, Smoldyn, and STEPS. While it will be a huge exercise to replicate their

entire model in another system, it would help to identify key numerical

stages in their approach which could be compared. Clearly the single-partical

reaction-diffusion calculations will run much slower, but a cross-test of

a short portion of the calculation would be reassuring.

3. Similarly, when the authors break the problem up over multiple GPUs, can they

illustrate a validation step to show that the result matches single-GPU or

even single-thread outcomes?

Minor comments.

4. While I acknowledge that it is difficult to encode all the model

details into SBML, it would help to have an SBML specification of their

reaction systems, ideally with the compartmental subdivisions. This provides

readers with access to several tools at least for the reaction topology

and the CME and well-mixed versions of the model.

5. The account of parameterization is painstaking, for which I compliment them,

but the number of items makes it difficult to follow. Can the

authors provide a summary table of what they did for each parameter,

to help the reader?

6. Section 4.1: Point ii. I'm puzzled at this comment. One normally takes bulk

rates as a staring point to estimate propensities, in this process the voxel

volumes are explicitly accounted for, and in the limit of many small voxels

comprising a single large volume the two converge. Can the authors clarify?

It is true that there are higher-order effects due to different local

propensities, but this does not seem to be what the authors refer to.

**********

**Have the authors made all data and (if applicable) computational code underlying the findings in their manuscript fully available?**

The PLOS Data policy requires authors to make all data and code underlying the findings described in their manuscript fully available without restriction, with rare exception (please refer to the Data Availability Statement in the manuscript PDF file). The data and code should be provided as part of the manuscript or its supporting information, or deposited to a public repository. For example, in addition to summary statistics, the data points behind means, medians and variance measures should be available. If there are restrictions on publicly sharing data or code —e.g. participant privacy or use of data from a third party—those must be specified.

Reviewer #1: Yes

Reviewer #2: Yes

Reviewer #3: Yes

**********

PLOS authors have the option to publish the peer review history of their article (what does this mean?). If published, this will include your full peer review and any attached files.

If you choose “no”, your identity will remain anonymous but your review may still be made public.

**Do you want your identity to be public for this peer review?**  For information about this choice, including consent withdrawal, please see our Privacy Policy.

Reviewer #1: No

Reviewer #2: No

Reviewer #3: No

**Figure resubmission:**

**Reproducibility:**

-->?>

# Author comment: Author response to Decision Letter 1

**DOI:** 10.1371/journal.pcbi.1014811.r002

2 Jun 2026

Attachment Submitted filename: Reply_Final_afterZLS.docx

# Peer review: Decision Letter 1

**DOI:** 10.1371/journal.pcbi.1014811.r003

**Authors:** Marc R Birtwistle

19 Jun 2026

Spatially Resolved Reaction–Diffusion Modeling Reveals Effects of Intracellular Spatial Heterogeneity on Yeast Galactose Network Dynamics

PLOS Computational Biology

Dear Dr. Luthey-Schulten,

Thank you for submitting your manuscript to PLOS Computational Biology. After careful consideration, we feel that it has merit but does not fully meet PLOS Computational Biology's publication criteria as it currently stands. Therefore, we invite you to submit a revised version of the manuscript that addresses the points raised during the review process.

Please submit your revised manuscript by Aug 19 2026 11:59PM. If you will need more time than this to complete your revisions, please reply to this message or contact the journal office at ploscompbiol@plos.org. When you're ready to submit your revision, log on to https://www.editorialmanager.com/pcompbiol/ and select the 'Submissions Needing Revision' folder to locate your manuscript file.

Please include the following items when submitting your revised manuscript:

* A letter that responds to each point raised by the editor and reviewer(s). You should upload this letter as a separate file labeled 'Response to Reviewers'. This file does not need to include responses to formatting updates and technical items listed in the 'Journal Requirements' section below.

* A marked-up copy of your manuscript that highlights changes made to the original version. You should upload this as a separate file labeled 'Revised Manuscript with Track Changes'.

* An unmarked version of your revised paper without tracked changes. You should upload this as a separate file labeled 'Manuscript'.

Kind regards,

Marc Birtwistle

Section Editor

PLOS Computational Biology

**Additional Editor Comments (if provided):**

Hello, thank you for the revision. One reviewer still has some remaining concerns regarding the simulation methods, and so an additional round of review is requested to address them. Specifically, some additional validation that the developed simulator is working as intended would be quite important for rigor and reproduciblity of the work. If the manuscript already presents such evidence or if it is elsewhere in the literature please do expand upon that. A major revision decision is given to allow for more time, in case it is needed. Please do let us know if you would need more time to complete the revision.

**Reviewers' comments:**

Reviewer's Responses to Questions

**Comments to the Authors:**

**Please note here if the review is uploaded as an attachment.**

Reviewer #1: I was happy to see that the authors performed a number of new and illuminating simulations, which greatly improved the manuscript in my view. They have resolved my concerns, and I congratulate them on their fine work!

Reviewer #3: Major point 1: Ground truth tests.

I understand that it is difficult to find ground-truth experiments.

By this comment I meant a simulation of an analytically well-defined

reaction-diffusion system, not a new experiment. I make a similar suggestion

below for validation against other simulators.

Major point 2: Reimplementation.

I appreciate the author's preparation of an SBML representation. That

very substantially improves the accessibility and reusability of their model.

I understand it is hard to reimplement the entire thing (though with AI,

who knows!).

But coming to the point below about the RDME, might they do some tests of

a simple 'toy' reaction-diffusion system to compare against other simulators,

specially particle-based onies? There are also extant RDME simulators,

mentioned below.

Point 3: GPU tests.

OK.

Minor Point: SBML encoding:

OK, this is very helpful

Minor Point: Table

OK, this is helpful

Point: RDME

This turns out to be a complex issue at the heart of their RDME method.

-> I'll start with pointing out previous work which addresses this:

The sections 4.1.2 and 4.1.3 look to me like a restatement of the

work on the problem of failure of RDME methods in the microscopic limit,

which is discussed in

Isaacson and Isaacson 2009 (Phys Rev E),

Isaacson 2013 (J Chem Phys), and

Gillespie, Petzold and Seitaridou 2014 (J Chem Phys).

The authors have mentioned the work of Hellander et al (2015) which follows

up from these. So it isn't clear to me what new insights these two sections

(4.1.2 and 4.1.3) bring. In any event, can the authors open these sections by

placing them clearly in context of the previous work?

-> I should also mention that the lattice diffusion model seems reminiscent of

NeuroRD, which uses a similar approach by Oliveira et al 2010, PMID: 20661441

There is also the Spatial Gillespie method (Next Subvolume Method 2014) and

MesoRD (Fange et al 2012, Bioinformatics).

Can the authors comment on how the logic of these existing tools for

handling propensity differs from their implementation of the RDME approach?

-> I worked through the logic of Supplementary section 5.

It is all straightfoward till the second last line, where the

author asserts (without clarity) that SB,j/SB ~ 1/300.

I would have gone with the logic that

* there is abundance SB in the cell

* The cell is subdivided into N subvoxels.

-> Therefore mean abundance of B (integrated over time) in one subvoxel =

SB,j = SB/N.

I do not understand how the authors get SB,j/SB ~ 1/300, which seems to

be the basis for the assertion that the propensity differs between CME and

RDME. My reading is that this may be a patch for a case where RDME fails to

work well in the microscopic limit. Can the authors comment?

**********

**Have the authors made all data and (if applicable) computational code underlying the findings in their manuscript fully available?**

The PLOS Data policy requires authors to make all data and code underlying the findings described in their manuscript fully available without restriction, with rare exception (please refer to the Data Availability Statement in the manuscript PDF file). The data and code should be provided as part of the manuscript or its supporting information, or deposited to a public repository. For example, in addition to summary statistics, the data points behind means, medians and variance measures should be available. If there are restrictions on publicly sharing data or code —e.g. participant privacy or use of data from a third party—those must be specified.

Reviewer #1: None

Reviewer #3: Yes

**********

PLOS authors have the option to publish the peer review history of their article (what does this mean?). If published, this will include your full peer review and any attached files.

If you choose “no”, your identity will remain anonymous but your review may still be made public.

**Do you want your identity to be public for this peer review?**  For information about this choice, including consent withdrawal, please see our Privacy Policy.

Reviewer #1: No

Reviewer #3: No

[NOTE: If reviewer comments were submitted as an attachment file, they will be attached to this email and accessible via the submission site. Please log into your account, locate the manuscript record, and check for the action link "View Attachments". If this link does not appear, there are no attachment files.]

**Figure resubmission:**

**Reproducibility:**

?>

# Author comment: Author response to Decision Letter 2

**DOI:** 10.1371/journal.pcbi.1014811.r004

17 Aug 2026

Attachment Submitted filename: Yeast_Reply2_ZLS_08142026TW.docx

# Peer review: Decision Letter 2

**DOI:** 10.1371/journal.pcbi.1014811.r005

**Authors:** Marc R Birtwistle

9 Sep 2026

Dear Dr. Luthey-Schulten,

We are pleased to inform you that your manuscript 'Spatially Resolved Reaction–Diffusion Modeling Reveals Effects of Intracellular Spatial Heterogeneity on Yeast Galactose Network Dynamics' has been provisionally accepted for publication in PLOS Computational Biology.

Before your manuscript can be formally accepted you will need to complete some formatting changes, which you will receive in a follow up email. A member of our team will be in touch with a set of requests.

Please note that your manuscript will not be scheduled for publication until you have made the required changes, so a swift response is appreciated.

IMPORTANT: The editorial review process is now complete. PLOS will only permit corrections to spelling, formatting or significant scientific errors from this point onwards. Requests for major changes, or any which affect the scientific understanding of your work, will cause delays to the publication date of your manuscript.

Should you, your institution's press office or the journal office choose to press release your paper, you will automatically be opted out of early publication. We ask that you notify us now if you or your institution is planning to press release the article. All press must be co-ordinated with PLOS.

Thank you again for supporting Open Access publishing; we are looking forward to publishing your work in PLOS Computational Biology.

Best regards,

Marc Birtwistle

Section Editor

PLOS Computational Biology

***********************************************************

Reviewer's Responses to Questions

**Comments to the Authors:**

**Please note here if the review is uploaded as an attachment.**

Reviewer #3: The authors have well addressed my concerns in this revision.

I appreciate the presentation of the prior work to show ground-truth

accuracy, and their deferring of a full performance analysis to an upcoming

paper.

The comparison with previous work is improved.

I note that they have thoroughly cleared up the ambiguity about the

estimate for SB,j/SB

**********

**Have the authors made all data and (if applicable) computational code underlying the findings in their manuscript fully available?**

The PLOS Data policy requires authors to make all data and code underlying the findings described in their manuscript fully available without restriction, with rare exception (please refer to the Data Availability Statement in the manuscript PDF file). The data and code should be provided as part of the manuscript or its supporting information, or deposited to a public repository. For example, in addition to summary statistics, the data points behind means, medians and variance measures should be available. If there are restrictions on publicly sharing data or code —e.g. participant privacy or use of data from a third party—those must be specified.

Reviewer #3: Yes

**********

PLOS authors have the option to publish the peer review history of their article (what does this mean?). If published, this will include your full peer review and any attached files.

If you choose “no”, your identity will remain anonymous but your review may still be made public.

**Do you want your identity to be public for this peer review?**  For information about this choice, including consent withdrawal, please see our Privacy Policy.

Reviewer #3: No

# Editor report: Acceptance letter

**DOI:** 10.1371/journal.pcbi.1014811.r006

**Authors:** Marc R Birtwistle

PCOMPBIOL-D-26-00367R2

Spatially Resolved Reaction–Diffusion Modeling Reveals Effects of Intracellular Spatial Heterogeneity on Yeast Galactose Network Dynamics

Dear Dr Luthey-Schulten,

I am pleased to inform you that your manuscript has been formally accepted for publication in PLOS Computational Biology. Your manuscript is now with our production department and you will be notified of the publication date in due course.

The corresponding author will soon be receiving a typeset proof for review, to ensure errors have not been introduced during production. Please review the PDF proof of your manuscript carefully, as this is the last chance to correct any errors. Please note that major changes, or those which affect the scientific understanding of the work, will likely cause delays to the publication date of your manuscript.

Soon after your final files are uploaded, unless you have opted out, the early version of your manuscript will be published online. The date of the early version will be your article's publication date. The final article will be published to the same URL, and all versions of the paper will be accessible to readers.

For Research, Software, and Methods articles, you will receive an invoice from PLOS for your publication fee after your manuscript has reached the completed accept phase. If you receive an email requesting payment before acceptance or for any other service, this may be a phishing scheme. Learn how to identify phishing emails and protect your accounts at https://explore.plos.org/phishing.

Thank you again for supporting PLOS Computational Biology and open-access publishing. We are looking forward to publishing your work!

With kind regards,

Janani Seenivasan

PLOS Computational Biology | Carlyle House, Carlyle Road, Cambridge CB4 3DN | United Kingdom ploscompbiol@plos.org | Phone +44 (0) 1223-442824 | ploscompbiol.org | @PLOSCompBiol
